# Advancing climate-resilient livestock systems: Next-generation emission mitigation strategies and integrated technological innovations

**DOI:** 10.1016/j.vas.2026.100588

**Published:** 2026-01-30

**Authors:** Navid Ghavi Hossein-Zadeh

**Affiliations:** Department of Animal Science, Faculty of Agricultural Sciences, University of Guilan, Rasht 41635-1314, Iran

**Keywords:** Climate-smart agriculture, Emission reduction technologies, Greenhouse gas mitigation, Livestock sustainability, Precision livestock farming

## Abstract

Livestock production significantly contributes to global greenhouse gas (GHG) emissions, particularly methane (CH₄), nitrous oxide (N₂O), and carbon dioxide (CO₂), posing challenges to climate change mitigation and environmental sustainability. This review explores advanced, system-wide approaches to reduce emissions from livestock systems while enhancing productivity, resilience, and resource efficiency. It covers short-term mitigation strategies such as dietary interventions—including methane inhibitors, microbial modulators, and natural compounds—that target enteric fermentation. Long-term solutions involve genetic and breeding innovations, such as microbiome-genome interaction analyses, CRISPR-based editing, and low-methane phenotyping, supported by genomic selection and precision phenotyping tools. The review also assesses advanced manure management technologies like anaerobic digesters and nutrient recovery systems, and examines precision livestock farming tools, including real-time sensors, machine learning models, UAVs, and IoT-based monitoring systems. Emerging digital tools, blockchain, augmented reality, and AI-assisted diagnostics are highlighted for enhancing traceability and decision-making. The potential of integrated energy systems, such as microbial fuel cells, hydrogen electrolysis, algae-based bioenergy, and thermal gasification, is discussed alongside traditional renewables, enabling livestock farms to become clean energy hubs. Circularity is emphasized through silvopasture, algal bioremediation, insect bioconversion, and integrated crop-livestock systems. Environmental assessment tools and the socio-political dimensions of technology adoption, including policy, education, and farmer behavior, are also considered. Future research directions, such as atmospheric methane oxidation, 4D-printed feed additives, and quantum modeling, are proposed. Overall, the review calls for a transdisciplinary, integrated approach to transform livestock systems into climate-smart, low-emission food production networks.

## Highlights

• Advances in manure management boost nutrient recovery and methane reduction.
• Precision livestock farming lowers emissions and improves animal welfare.
• Renewable energy integration transforms farms into net energy producers.
• IoT, AI, and drones optimize feeding, grazing, and emission monitoring.
• Policy insights reveal pathways to scalable, sustainable livestock systems.

## Graphical abstract

**Figure:**
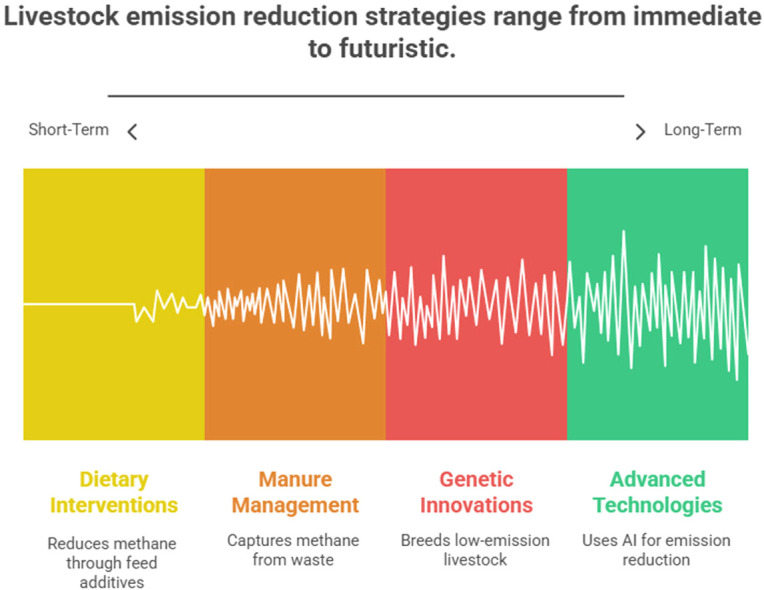

## 1 Introduction

**Fig. 1:**
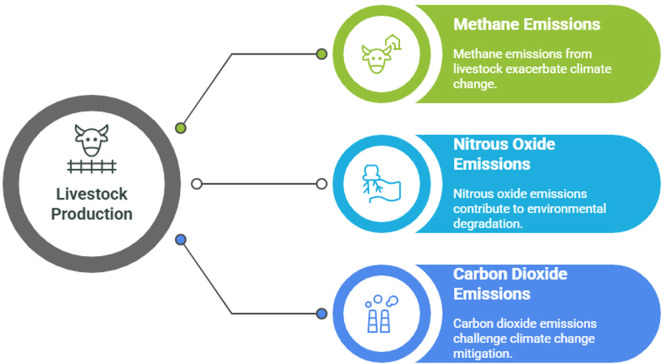
Major sources of GHG emissions in livestock production.

Livestock production systems play a dual and globally significant role in food security, livelihoods, and environmental sustainability. In addition to supplying essential animal-source foods that contribute to human nutrition and income generation for more than one billion people worldwide, particularly in low- and middle-income countries, livestock systems are also recognized as major contributors to atmospheric warming through their significant emissions of CH₄ and N₂O (Hörtenhuber et al., 2022). According to recent analyses from the FAO's Global Livestock Environmental Assessment Model (GLEAM), about 6.2 gigatonnes of CO₂e emissions, or around 12% of all anthropogenic greenhouse gas emissions, were caused by livestock agrifood systems worldwide in 2015, which include cattle, buffaloes, sheep, goats, pigs, and chickens (Fig. 1). Cattle farming, which includes the production of both meat and milk, was responsible for about 62% of these livestock emissions, or 3.8 gigatonnes CO₂e annually (FAO, 2023). Enteric fermentation, a ruminant's natural digestive process that turns fibrous feeds into strong greenhouse gases, is the main cause of this methane burden (Russo, 2024; Mrutu et al., 2025). Modern manure storage methods exacerbate this problem by providing the perfect environment for bacteria that produce N₂O and CH₄-producing archaea, which together produce a hazardous atmospheric cocktail (Zahra et al., 2024; Symeon et al., 2025). The urgency of mitigation is underscored by evidence that current livestock-related emissions could consume approximately 15% of the remaining global carbon budget compatible with the 1.5°C target, emphasizing the need for near-term and long-term action (Arndt et al., 2022; FAO, 2023). When regional differences are taken into account, the situation becomes more complicated. For example, expanding pastoral systems in sub-Saharan Africa contribute to emissions from land-use change, while industrial-scale operations in North America and Europe create concentrated emission hotspots (Adlan et al., 2024; Leitner et al., 2024). According to new atmospheric modelling techniques, agricultural CH₄ emissions from major livestock grazing areas are now responsible for some of the observed warming of the Arctic (Wittig et al., 2023). These emissions interact with climate feedback mechanisms in a worrying way: warmer temperatures accelerate methane releases from melting organic grassland and increase heat stress on livestock (Lan & Dlugokencky, 2024). Excess nitrogen from manure volatilization contributes to dead zones in coastal ecosystems and ozone layer depletion, causing interrelated problems that defy easy fixes. These multi-gas and cross-scale interactions highlight the need for mitigation strategies that address both short-lived climate pollutants and long-term nitrogen cycle disruptions (Lan & Dlugokencky, 2024; Lee et al., 2024; Parmentier et al., 2024).

Current analyses highlight the significant economic and environmental effects of greenhouse gas emissions from livestock systems, underscoring their relevance not only to climate policy but also to global food-system sustainability and rural development objectives. Animal agriculture's geographic expansion and intensification have increased attention to the industry's climate accounting, making it a crucial component of international mitigation plans. The emission intensities of various production systems, such as intensive operations and extensive grazing, differ greatly because of variations in land-use practices, feed composition, and manure management. The magnitude and type of emissions across the livestock sector are influenced by wider structural and technological differences, which are reflected in these regional and system-level variations (Gingrich et al., 2024; Peng et al., 2024a; Recktenwald & Ehrhardt, 2024).

Enteric methane emissions, which are mostly produced by ruminants, continue to be a major source of GHG emissions in agriculture because of their strong warming potential and brief atmospheric lifetime. This dual characteristic presents both an immediate climate risk and a strategic opportunity for near-term mitigation, making methane reduction a central lever in climate action. The supply chains' use of fossil fuels, emissions from manure handling systems, and changes in land use for feed production all contribute to the sector's increased environmental impact. Particularly in areas where production is disconnected from ecological capacity or where technological and regulatory interventions are still scarce, the concentration of livestock in a given area intensifies these effects (Holmes, 2024; Place, 2024).

While technological solutions such as feed additives, anaerobic digestion, and improved manure management are available and have demonstrated strong mitigation potential, their real-world implementation remains uneven and context-dependent. Many existing strategies are constrained by scalability, cost, regulatory uncertainty, or system-specific effectiveness, limiting their impact beyond experimental or highly controlled settings. These limitations highlight the insufficiency of fragmented, single-intervention approaches and underscore the need for coordinated system-level solutions. Current policy frameworks often fall short of incentivizing widespread implementation, despite the sector's comparative cost-effectiveness in delivering emission reductions relative to other major industries. Furthermore, the sector’s contribution to land-use change, particularly deforestation and degradation linked to feed production, continues to undermine climate mitigation gains. These challenges are compounded by the growth of livestock populations in response to increasing global demand for animal-source foods, particularly in low- and middle-income countries (Faizan, 2024; Holmes, 2024; Yan et al., 2024).

However, there are also special chances for revolutionary climate action in livestock systems. The basis for coordinating livestock production with climate and sustainability objectives is provided by developments in precision farming, the integration of renewable energy, and circular bioeconomy models. Such advances represent emerging technological opportunities capable of transforming livestock systems if deployed in integrated and context-sensitive ways. Realizing the sector's strategic importance to national and international emissions targets, new financial mechanisms and international commitments have started to direct investments toward the mitigation of livestock-related issues. Nonetheless, there is still a significant disconnect between technical potential and actual impact, indicating the urgent need for next-generation, integrated innovation pathways that move beyond isolated interventions (Beck et al., 2024; Godara et al., 2024). The livestock industry is at a critical juncture in this regard. Continued emissions growth could seriously impede the achievement of global climate targets if decisive action is not taken. However, livestock systems have the potential to change from being a significant source of emissions to a crucial contributor to climate stabilization with focused policy support, scientific advancement, and cross-sectoral coordination (Choudhary & Palsaniya, 2024; Holmes, 2024).

In this context, the ability of livestock systems to anticipate, absorb, adapt to, and recover from climate-related shocks while preserving productivity, environmental integrity, and socio-economic viability is known as climate resilience (Pankaj et al., 2024). The interplay between biological, environmental, and socioeconomic systems, especially in areas where development pressures and climatic variability converge, makes the complexity of GHG emissions associated with livestock more apparent. The tripartite architecture of livestock systems, which includes feed production, manure management, and enteric fermentation, results in emissions that are closely related to climate feedback mechanisms. These emissions are influenced by larger agroecological and socioeconomic contexts that affect both mitigation potential and climate change vulnerability, in addition to animal physiology and management practices (Gingrich et al., 2024; Symeon et al., 2025).

Methanogenic archaea in the rumen, which break down hydrogen and carbon dioxide generated during fermentation, are the main source of enteric methane emissions. Dietary composition affects these processes; feedstocks high in lignin and fiber tend to digest more slowly and produce more methane. On the other hand, some feeding techniques may worsen fermentation pathways driven by acetate, which would result in an even higher production of methane. Methane-inhibiting feed additives, which target enzymatic pathways unique to methanogenesis, are still not widely used across regions and production systems, particularly in places with limited resources or no access to supplements. This uneven adoption underscores socio-economic disparities and raises important questions regarding equity and climate justice in global mitigation efforts (del Prado et al., 2024; Faizan, 2024; Xie et al., 2025).

Another level of complexity arises with manure management, especially in areas with inadequate waste treatment infrastructure. Smaller or more conventional systems frequently rely on open-pit or unprocessed storage techniques, even though industrial operations may use closed-loop systems, like anaerobic digesters, to capture biogas (Souza et al., 2025). These circumstances may cause emissions to fluctuate, especially during severe weather events that affect nitrogen cycling and microbial activity (Badzmierowski et al., 2025). In these situations, weather conditions, like high temperatures or more precipitation, can accelerate the microbial conversions of nitrogenous compounds in manure, increasing the production of methane and nitrous oxide. These feedbacks between environmental factors and microbial activity in waste management systems are probably going to get stronger as the climate system continues to change (Bele et al., 2024; Bumharter, 2024; Symeon et al., 2025).

The manufacturing of animal feed also directly and indirectly adds to the emissions profile of the industry. Because they can use existing biomass streams and lessen the pressure on land use, agricultural by-products and unconventional feed sources have been investigated as mitigation strategies. These substitutes, however, frequently have trade-offs with regard to scalability, nutritional sufficiency, or unforeseen environmental effects like nutrient runoff or modifications to soil chemistry. Additional variability is introduced by the climate sensitivity of feed supply chains. Crop availability is impacted by climate disruptions, and livestock systems' dietary practices may change as a result, potentially increasing emissions if digestibility and fermentation profiles are adversely affected (Wang et al., 2024a; Sabia et al., 2025).

It is becoming more and more clear that there are feedback loops between the production of livestock and the larger climate system. New emission dynamics are produced by the interaction of microbial, chemical, and behavioral processes in livestock systems with changes in seasonal temperature and precipitation regimes, global trade patterns, and feedstock inputs. The temporal and spatial signatures of these emissions are being revealed by recent developments in atmospheric trace gas detection and remote sensing, which have revealed patterns more closely associated with human management practices than with natural biogeochemical cycles. These findings demonstrate the increasing human influence on agricultural emissions and the pressing need for integrated innovation, defined here as the coordinated application of biological, technological, digital, and management-based solutions across multiple system components, to address emissions effectively (Choudhary & Palsaniya, 2024; Gingrich et al., 2024; Symeon et al., 2025).

The need to balance several, sometimes conflicting, priorities is influencing the technological transformation of livestock systems more and more. These include social justice, ecological restoration, climate resilience, and emissions reduction, especially in areas where agricultural practices are strongly ingrained in environmental, gendered, and cultural contexts. Emerging strategies increasingly demand inclusive frameworks that combine state-of-the-art technologies with regional knowledge systems and culturally grounded practices, as opposed to exclusively depending on top-down innovation (Eswaran et al., 2024; Holmes, 2024).

Many communities that depend on livestock, particularly those in climate-sensitive areas, are co-developing technological solutions in ways that respect gender roles, traditional land stewardship, and current agroecological practices. Community-led projects, for instance, have demonstrated that combining solar-powered technology, drought-tolerant forage systems, and decentralized feed production can improve labor equity and food system sovereignty in addition to reducing emissions. Women play a particularly important role in the management and care of livestock in these situations, and gender-responsive innovations are helping to achieve both empowerment and mitigation goals (Adhikary & Pant, 2024; Morgan et al., 2024).

Livestock systems based on agroforestry also show how ecological restoration and emission reduction can work together. A prime example of how hybrid management models, which are based on both tradition and science, can improve forage quality, sequester carbon, and support biodiversity is the thoughtful placement of nitrogen-fixing trees into grazing landscapes (Angerer et al., 2021; Sabia et al., 2025). These trees are managed using locally informed techniques and monitored using emerging sensing technologies. The growing importance of indigenous ecological knowledge in climate-smart livestock practices is reflected in these integrative approaches (Goracci & Camilli, 2024; VijayKumar et al., 2024).

Improved data mining and data analysis are also being developed to take account of cultural taxonomies and behavioral insights specific to pastoralist and smallholder systems. With the integration of traditional classifications of animal behavior or pasture quality in machine learning algorithms, emission and productivity prediction models become more accurate, more contextually aware, and more acceptable to end-users. Such a convergence of digital tools with ancestral knowledge reinforces the view that technology adoption will be most effective if it respects and builds on existing local frameworks (Himu & Raihan, 2024; Vlaicu et al., 2024). Nonetheless, there are still significant differences in access to mitigation technologies between and within regions. Although high-tech solutions like virtual fencing or automated methane scrubbing systems may have a lot of potential for mitigation, their deployment in lower-income or community-based systems is frequently constrained by their cost, infrastructure, and cultural fit. As a result, grassroots ideas have surfaced that reinterpret emission reductions as a co-benefit of more general sustainability and livelihood objectives (Bergougui et al., 2024; Jiao & Tan, 2024).

These developments underscore the importance of approaching livestock innovation not as a quest for universal technological fixes but as a process of co-evolution between systems, societies, and environments. As socioecological scaffolds, climate-smart technologies must be flexible instruments that integrate into cultural contexts, address gender dynamics, and correspond with locally established success metrics. In this way, they can help foster equitable transitions to low-emission livestock systems that are both resilient and regenerative (Pankaj et al., 2024; Awoke et al., 2025).

Against this backdrop, the present review systematically examines progress in the mitigation of GHG emissions from livestock production (Table 1) to advance a system-level, integrative perspective that bridges biological mechanisms, technological innovations, and socio-economic considerations. Unlike many existing reviews that focus on individual mitigation measures, this review explicitly synthesizes next-generation biological, technological, and socio-economic innovations within a unified framework. While previous reviews have often focused on individual mitigation options, this work addresses three interlinked gaps in the literature. First, although methodological innovation in the field has grown exponentially, especially in the areas of biochar-augmented anaerobic digestion systems and CRISPR-engineered methanogen suppression, there is still a critical lack of agreement regarding the best feedstock ratios for biochar in terms of maximizing CH₄ capture versus its long-term effects on the retention of nutrients in manure. Second, rigorous evaluation of the regional scalability of closed-loop technologies, such as drone-based pasture management systems and high-rate biogas reactors with integrated biochar filters, has not kept pace with the practical deployment of these technologies, especially when it comes to the durability of IoT sensors in harsh climates and the cost of producing biochar in low-resource settings. Lastly, new connections between low-tech solutions (e.g., farm-scale kilns for biochar) and high-precision instruments (e.g., AI-powered methane prediction models) necessitate hybrid frameworks that strike a balance between short-term affordability and long-term effectiveness. These gaps were addressed by synthesizing recent findings, evaluating system-level mitigation pathways, and highlighting unexplored applications such as nanoparticle-enhanced biochar composites for simultaneous N₂O suppression and phosphorus recovery. The remainder of this review is organized to guide the reader from biological mitigation mechanisms through technological innovations and integrated system-level strategies, concluding with policy implications and future research directions, thereby aligning livestock production with climate resilience and sustainable development goals.

**Table 1:** Comparison of mitigation technologies for reducing GHG emissions in global livestock systems.

Technology	GHG reduction potential (%)^1^	Scalability^2^	Cost (US$/ton CO₂e)^3^	Current adoption (%)^4^	Primary GHG targeted	Recent references
3-NOP (3-nitrooxypropanol)	25–45	Medium–High	20–100	<2	CH₄	Amancio et al. (2025) van de Gucht et al. (2025)
Asparagopsis spp. (red seaweed)	30–80	Low–Medium	50–200	<1	CH₄	Angellotti et al. (2025) Thorsteinsson et al. (2025)
Nitrate supplementation	10–30	Medium	20–60	10–15	CH₄	Bharanidharan et al. (2025) Bae et al. (2025)
Probiotics / Direct-fed microbials	5–15	High	10–30	20–30	CH₄, N₂O	Jeong et al. (2024) Olorunlowu et al. (2025)
Methanogen vaccines	20–30 (projected)	Low (R&D phase)	TBD	<1 (experimental)	CH₄	Malyugina et al. (2025) Muntari et al. (2025)
Anaerobic digestion (biogas systems)	40–70	Medium–High	0–80	10–20	CH₄ from manure	Mishra et al. (2025) Subramanian and Suresh (2024)
Biochar addition to manure	10–25	Medium	20–80	<10	CH₄, N₂O	Luo et al. (2024) Tiong et al. (2024)
Electrochemical ammonia recovery	20–40	Low–Medium	50–150	<5	N₂O	Wang et al. (2024c) Huang et al. (2024b)
Precision feeding (automated systems)	5–15	High in developed regions	10–40	15–25	CH₄, CO₂	Ghavi Hossein-Zadeh (2025) Kamau et al. (2025)
AI-driven methane prediction	5–20	Medium	30–70	<10	CH₄	Altshuler et al. (2025b) Alemu et al. (2025)
Low-emission livestock breeding	10–30 (long-term)	High	5–30 (opportunity cost)	20–40	CH₄	Ryan et al. (2025) Worku (2024)
CRISPR microbiome engineering	30–50 (projected)	Low	TBD	<1 (early stage)	CH₄	Mrutu et al. (2025) Malyugina et al. (2025)
Solar-powered barn technologies	5–10	Medium	40–100	10–20	CO₂ (energy-related)	Pal et al. (2025) Murali et al. (2024)
Biogas-to-energy conversion systems	40–70	Medium	20–60	15–30	CH₄, CO₂	N’guessan et al. (2025) Akash et al. (2025)
Insect-based feed substitution	10–20	Medium	30–90	<10	Indirect (feed emissions)	Kichamu et al. (2025) Jafir et al. (2024)
Silvopastoral and rotational grazing	15–40	Medium–High	5–30	20–35	CO₂ (land use), CH₄	Brunetti et al. (2025) Gonzalez Quintero et al. (2024)

1 Estimates of GHG reduction potential vary based on species, management systems, and regional contexts.

2 Scalability is based on infrastructure requirements, market maturity, and regulatory factors.

3 Cost presents the estimated mitigation cost per metric ton of CO₂e avoided.

4 The estimated global adoption rate may differ greatly depending on the nation or production system.

## 2 Methodological approach and literature search strategy

The manuscript was formulated as a narrative and integrative review aimed at synthesizing, critically reviewing, and conceptually integrating the current and emerging studies on greenhouse gas (GHG) emission mitigation and technological advancements in livestock systems. Since the field is interdisciplinary and aims to introduce innovative methods and nutrition, as well as explore aspects of rumen microbiology, genetics, engineering, digital agriculture, environmental evaluation, and socio-policy, a systematic review or meta-analysis was deemed methodologically unsuitable. Rather, a systematic narrative structure was adopted whereby the heterogeneous evidence, emerging technologies, and prospective ideas that cannot be quantitatively synthesized could be incorporated.

The literature search was conducted between January 2025 and July 2025, and included peer-reviewed articles published from January 2000 onwards, with a priority given to those published in 2020 and later. The search was performed using Web of Science, Scopus, PubMed, ScienceDirect, and Google Scholar. Moreover, the selected reports of international bodies (e.g., FAO and IPCC) and policy-relevant technical documents were used as the background to shed some light on the situation and offer some policy-specific information.

The search terms were constructed in an iterative way, and the combination of search terms with the help of Boolean operators implied the multidimensional nature of the review. The keywords were the combinations of livestock greenhouse gas emissions, mitigation of enteric methane, rumen microbiome manipulation, feed additives, 3-nitrooxypropanol, seaweed-based inhibitors, precision livestock farming, artificial intelligence, Internet of Things, manure management technologies, anaerobic digestion, biochar, circular bioeconomy, genomic selection, CRISPR-based interventions, and climate-smart livestock systems. The key publications' reference lists were also screened manually to determine other relevant studies.

The selection of literature was done by the use of relevance- and quality-focused predetermined inclusion criteria. The studies were considered to include those that: (i) they dealt with mitigation of enteric or manure-related GHG emissions in livestock systems; (ii) they provided mechanistic, experimental, modeling, or systems-level information about mitigation strategies; (iii) they evaluated enabling technologies, including tools of precision livestock farming, digital monitoring systems, or integrated energy and circular bioeconomy systems; or (iv) they provided understandings about climate-resistant livestock production, which were either environmental, economic, or policy-relevant.

It was focused on peer-reviewed publications that proved the methodological transparency, reproducibility, and applicability of the studies to the modern or novel mitigation pathways. The selection of review articles and policy reports was selective based on the fact that they were authoritative syntheses or contextual grounding. The exclusion criteria were that the studies had to concentrate on non-livestock agricultural systems, had to be data-lacking in scientific foundation, anecdotal evidence with no analytical backing, or that they were outdated and had little information to be useful in mitigation issues nowadays. No limits were placed on these factors, such as livestock species, system of production, or geographical area.

This manuscript is a narrative and integrative review, and no risk-of-bias scoring or quantitative bias assessment was conducted at the study level, as these methods are mainly aimed at systematic reviews and meta-analyses. However, the possible biases were also clearly taken into account by utilizing qualitative critical analysis of particular studies. Such an appraisal was as follows: study design (for example, *in vivo* or *in vitro* experiment, field or laboratory study), sufficiency of sample size, transparency of methods, reproducibility of results, and consistency with known biological, environmental, or engineering laws. Special consideration was given to possible publication bias in support of positive mitigation effects, short-term effects of experiments, or contextual results that could be extended with little success. Where divergent or conflicting results were reported, they were explicitly debated, and mitigating claims were explained carefully based on the methodological constraints of the system, system constraints, and trade-offs between the environment. This qualitative factor of bias allows for a balanced interpretation, and it is yet adequate in a conceptual synthesis.

Although this review is not a systematic review or a meta-analysis, the most important principles of the Preferred Reporting Items for Systematic Reviews and Meta-Analyses (PRISMA) guidelines were used in a modified form, which increases the level of transparency and clarity (Moher et al., 2009). These guidelines worked out the clear reporting of literature, search strategies, inclusion criteria, and logic of synthesis. In accordance with the existing methodological advice on narrative reviews, a formal PRISMA flow diagram, statistical synthesis, or risk-of-bias scoring tool was not present. A qualitative, thematic, and systems-oriented approach to the synthesis of the selected literature was used. Research works were classified according to significant thematic areas, seven of which are: dietary interventions, microbial and genetic solutions, manure management technologies, precision livestock farming instruments, integrated energy systems, and circular bioeconomy models.

The evidence within and across these domains was compared to determine similar mechanisms, synergies, trade-offs, scalability limits, as well as gaps in knowledge. Cross-domain integration was then used to intersect the biological, technological, environmental, and socio-economic viewpoints and allow a coherent system-level interpretation.

The presented integrative synthesis method enables identifying new trends and directions of innovations and recognizing uncertainty and heterogeneity among studies, which is a solid and progressive basis to improve climate-resilient low-emission livestock systems.

## 3 Feed additives and dietary interventions

### 3.1 Methane inhibitors

Methane inhibitors targeting the archaeal methanogens that convert metabolic hydrogen to CH₄ in the fermentation process are a key strategy to reduce emissions from enteric fermentation. These substances act via a variety of biochemical pathways and can be broadly classified into three groups: metabolic hydrogen re-routers, electron transport inhibitors, and direct enzyme inhibitors. Each of these groups has unique trade-offs in terms of translational feasibility, specificity, and effectiveness (Ni et al., 2025; Xie et al., 2025). Chemical inhibitors such as analogues of halogenated methane (e.g., bromochloromethane) and substances that contain nitrooxy (e.g., by attaching to methyl coenzyme M reductase (MCR) or essential cofactors, 3-NOP) directly inhibits methanogenesis, whereas biological agents, like vaccines that target methanogen surface proteins, trigger host immune responses to lower archaeal populations (Dinh & Allen, 2024; Ni et al., 2025). Tannins and essential oils are examples of plant-derived inhibitors that have broader antimicrobial effects, but frequently at the expense of compromising the digestion of fiber. The key challenge is to balance methane suppression with the functionality of the rumen, and successful inhibitors must target methanogens selectively without destabilizing the microbial community that is necessary for the formation of volatile fatty acids (Abd El Tawab et al., 2024; Susanto et al., 2024). By precisely targeting conserved methanogen pathways, recent developments in metagenomics and CRISPR-based microbial engineering have overcome previous constraints of microbial adaptation and resistance (Du et al., 2024a). The transition to mechanism-driven design is best illustrated by 3-NOP's irreversible MCR inhibition mechanism, which achieves consistent methane reductions in dairy systems (Costigan et al., 2024). The effectiveness of inhibitors is still context-dependent, though, and is influenced by ruminant species, diet composition, and dosage guidelines. For instance, high-forage diets increase methanogen resistance to specific substances because they prolong rumen retention periods. Adoption of inhibitors is also influenced by safety and regulatory factors, such as environmental persistence and residues in animal products (e.g., halogenated compounds), which, in spite of their strength, restrict some options (Lambo et al., 2024; Ni et al., 2025; Xie et al., 2025). Hybrid strategies now include inhibitors with hydrogenotrophic probiotics or soil amendments that oxidize methane, transforming inhibition approaches from stand-alone solutions to synergistic components of circular farming systems. Methane inhibitors are positioned by this changing paradigm as catalysts for reengineering rumen metabolism toward climate-resilient livestock production, rather than just as instruments for reducing emissions (Abd El Tawab et al., 2024; Holmes, 2024; Patel & Pata, 2024).

Although methane inhibitors have shown significant potential to decrease enteric methane emissions, their overall implications on the quality of products of animal origin and the outcomes of food systems deserve deep attention. Rumen mitigation approaches that are able to modify rumen fermentation pathways are able to control nutrient partitioning, energy use, and lipid metabolism, thus affecting milk and meat properties. Emissions must be reduced without jeopardizing the quality of the products, safety, and consumer acceptance of the products to ensure sustainable adoption of the technologies (Malyugina et al., 2025).

Methane inhibitors have been demonstrated to cause inconsistent effects on the yields and composition of milk, especially in the fat and protein levels produced in dairy production systems (Pepeta et al., 2024). A change in ruminal hydrogen balance and volatile fatty acid balance can either decrease de novo fatty acid production in the mammary gland or alter the relative ratios of short- and long-chain fatty acids in milk fat. These modifications may affect the functional characteristics of milk, i.e., coagulation behavior, curd firmness, and ripening dynamics, which are of special interest to cheese and fermented dairy products. Changes in the fatty acid composition of milk can also influence nutritional characteristics, some of which are linked to higher levels of unsaturated or polyunsaturated fatty acids, and responses to each type of inhibitor are extremely dependent on the type of inhibitor, diet composition, and lactation stage (Altshuler et al., 2025a; Ryu et al., 2025).

Methane inhibitors have been found to affect growth performance, feed conversion efficiency, and carcass traits in meat-producing systems and indicate the effects on energy efficiency and metabolic allocation. The alterations in muscle lipid structure can influence the most important quality characteristics of tenderness, juiciness, and oxidative stability, which can have implications for the shelf life and sensory attributes. The effects of the altered content of fatty acids could be either improving the nutritional value of meat products or making them more vulnerable to lipid oxidation, leading to processing and storage difficulties. These results indicate the need to consider methane inhibitors based on their capacity to reduce the intensity of emissions and their impact on the quality of carcasses and meat functionality (Fouts et al., 2022; Yanza et al., 2022; Pedrini et al., 2023).

Another dimension that is important in the analysis of methane inhibitors is food safety issues. The possibility of additive residues or metabolic by-products in milk or meat provokes regulatory and toxicological issues that have to be measured in a stringent evaluation. Despite the large number of inhibitors that are constructed to degrade quickly or are transformed into inert molecules, it is crucial that products are devoid of residues to be approved by regulatory bodies and be trusted by people. In addition to safety, consumer perception and acceptance are determining factors in uptake on the market, especially when the additives are seen to be synthetic or new. Openness, proper communication, and compliance with consumer demands in terms of naturalness and sustainability are thus key elements of successful implementation of methane mitigation technologies (Eason & Fennessy, 2023; Thorsteinsson & Nielsen, 2025).

**Fig. 2:**
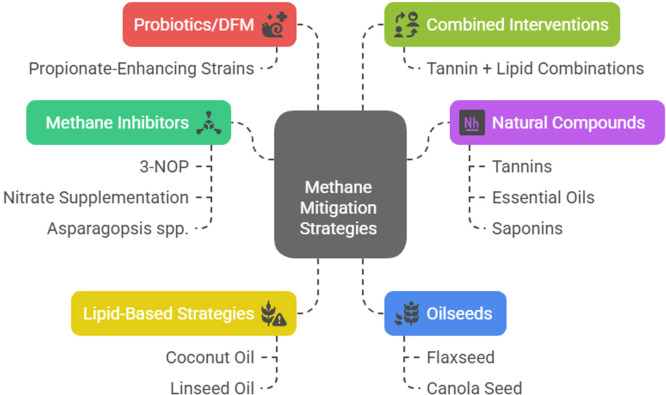
Feed additives and dietary interventions for methane mitigation in livestock.

In both dairy and beef systems, there have been discrepancies in the recorded product-level change, which underlines the situation-specificity of methane inhibitor reactions. The presence of variability in animal genetics, basal diet, management practices, inhibitor formulation, and length of supplementation are contributing factors to the lack of similarity in the results of studies. These discrepancies support the necessity of standardized experimental conditions, long-run assessments, and combined tests that will concomitantly address the parameters of emissions, animal performance, product quality, and safety (Cooke et al., 2024; de Ondarza et al., 2024; van Gastelen et al., 2024). An overview of feed additives and dietary interventions for methane mitigation in livestock is presented in Table 2 and Fig. 2.

**Table 2:** An overview of feed additives and dietary interventions for methane mitigation in livestock.

Category	Intervention	Mechanism	Efficacy (CH₄ reduction)	Advantages	Limitations	Recent references
Methane inhibitors	3-NOP (3-nitrooxypropanol)	Inhibits methyl-coenzyme M reductase in the methanogenesis pathway	25–45%	High efficacy, consistent results in cattle	Cost, regulatory approvals, and limited availability in some regions	Amancio et al. (2025) van de Gucht et al. (2025)
Methane inhibitors	Nitrate supplementation	Alternative hydrogen sink, reduces H₂ available to methanogens	10–25%	Inexpensive, improves nitrogen utilization	The risk of nitrate toxicity (methemoglobinemia) requires careful ration balancing	Bharanidharan et al. (2025) Bae et al. (2025)
Methane inhibitors	*Asparagopsis* spp. (Seaweed)	Bromoform disrupts methanogenic enzyme systems	30–80%	High efficacy at low inclusion levels	Bromoform concerns, scalability, supply chain limitations	Angellotti et al. (2025) Thorsteinsson et al. (2025)
Natural compounds	Tannins	Binds proteins and inhibits methanogens and protozoa	8–20%	Natural, enhances protein bypass, antimicrobial effects	Variability by source, potential anti-nutritional effects	Zhao et al. (2025) Carrazco et al. (2025)
Natural compounds	Essential oils	Disrupt microbial membranes and inhibit methanogens	5–12%	Natural, synergistic with other additives	Inconsistent results, palatability issues	Nasir et al. (2025) Benchaar and Hassanat (2025)
Natural compounds	Saponins	Suppress protozoa, indirectly reducing methanogens	4–10%	Natural, potential rumen modulation benefits	Dose sensitivity, inconsistent efficacy across diets	Carrazco et al. (2025) Zhang et al. (2025b)
Lipid-based strategies	Coconut oil and linseed oil	Reduces protozoa, biohydrogenation, and shifts rumen fermentation	10–20%	Readily available, dual function (energy + mitigation)	May reduce fiber digestibility at high doses	Roskam et al. (2025) Van Mullem et al. (2025)
Oilseeds	Flaxseed and canola seed	Similar to oils, they provide unsaturated fats and co-benefits	8–15%	Nutritional value, easy to incorporate into rations	High costs. They require inclusion limits	Akter et al. (2025) Vinyard et al. (2025)
Probiotics/DFM	Propionate-enhancing strains	Modify fermentation pathways toward propionate rather than methane	3–8%	Safe, generally recognized as safe (GRAS) status	Limited methane-specific strains, low efficacy	Jeong et al. (2024) Olorunlowu et al. (2025)
Combined interventions	Tannin and lipid combinations	Multiple targeted inhibition of methanogenesis and fermentation	20–35%	Synergistic effects. They may improve animal performance	Complex interaction. They need balancing	Foggi et al. (2024) Rossi et al. (2022)

#### 3.1.1 3-NOP (3-nitrooxypropanol) mechanisms

A synthetic substance called 3-nitrooxypropanol (3-NOP) was created as a specific inhibitor of ruminant enteric methane production. The biochemical inhibition of methyl-coenzyme M reductase (MCR), a crucial enzyme in the last stage of methanogenesis, is at the heart of its mode of action. In terms of structure, 3-NOP has a nitrooxy functional group that resembles MCR's natural substrate and allows it to bind to the enzyme's active site competitively. 3-NOP efficiently prevents the terminal electron transfer necessary for methane synthesis in methanogenic archaea by obstructing the catalytic conversion of methyl-coenzyme M to methane (Mshary et al., 2024a; Ni et al., 2025).

Beyond direct enzymatic inhibition, 3-NOP also disrupts the methanogen energy metabolism by interrupting the membrane-associated electron transport chain. This dual action impairs ATP synthesis and disrupts proton gradients across archaeal membranes, reducing the overall metabolic activity of methanogens. This mechanism is notably more specific than earlier inhibitors, which often affected a broader range of rumen microbes and risked compromising fiber digestion and nutrient absorption (van Lingen et al., 2021; Xuan et al., 2024).

The selectivity of 3-NOP is a crucial benefit. 3-NOP is made to exclusively target methanogenic pathways, as opposed to non-specific antimicrobials, reducing the likelihood of side effects on beneficial rumen microbiota like fiber-digesting bacteria and protozoa. Therefore, even in situations where methane output is decreased, animal performance metrics like milk fat yield and fiber digestibility are usually maintained (Martínez et al., 2024).

After consumption, 3-NOP is quickly broken down in the rumen into inert metabolites that the body can safely get rid of through regular physiological functions. By lowering worries about residue buildup in animal products, this metabolic breakdown helps to create a positive safety profile. However, rumen retention time and diet composition affect how effective it is. Diets high in fermentable carbohydrates, for instance, may improve 3-NOP solubility and retention, which would result in a more noticeable suppression of methane. Due to faster ruminal passage rates, high-fiber diets, on the other hand, may lessen their effectiveness. For this reason, microencapsulated delivery systems with improved bioavailability and sustained release under grazing conditions have been developed (Yu et al., 2021; Lahart et al., 2024; Xuan et al., 2024).

A short-term buildup of hydrogen in the rumen is one of the metabolic effects of inhibited methanogenesis, which may be diverted to other fermentation pathways. Non-methanogenic bacteria that use hydrogen to create propionate, a host-beneficial volatile fatty acid that produces energy, may become more active as a result of this hydrogen redirection. This microbial shift improves the overall efficiency of feed energy utilization in addition to promoting productive fermentation (Jeong et al., 2024; Ungerfeld & Pitta, 2024).

The goal of recent developments in delivery technologies has been to maximize the release and dosage of 3-NOP. To adapt delivery to changing rumen conditions, encapsulation techniques employing pH-sensitive polymers and real-time rumen monitoring systems have been investigated. Despite these advancements, 3-NOP's activity is mostly limited to the rumen, and it is ineffective at reducing methane emissions from post-excretion manure. In order to accomplish thorough emission reductions across the livestock production cycle, integrated mitigation strategies, combining 3-NOP with downstream interventions like biochar-amended manure management, are being researched (Costigan et al., 2024; Hassen et al., 2024).

Accessibility for smallholder systems, production costs, and regulatory frameworks are some of the factors that continue to impact the wider adoption of 3-NOP. Formulations that combine 3-NOP with nutrient supplements for convenience of use in resource-constrained environments are among the ongoing research efforts to increase affordability and scalability. Furthermore, the potential of hybrid formulations that combine 3-NOP with other methane inhibitors, like those made from red seaweed species, to act on complementary biochemical targets within the methanogenesis pathway is being assessed. These pairings have the potential to maintain or enhance animal health and productivity while synergistically reducing methane emissions (Hassen et al., 2024; Pedrini et al., 2024).

#### 3.1.2 Seaweed derivatives (*Asparagopsis* spp.)

One of the most promising biological feed additives for lowering ruminant enteric methane emissions is seaweed derivatives derived from *Asparagopsis* species. The main reason for their effectiveness is the existence of halogenated substances, like bromoform and bromochloromethane, which interfere with several biochemical processes essential to methanogenesis. These substances work in different ways, such as interfering with the MCR's activity and the cobalamin-dependent enzymatic pathways in methanogenic archaea (Wanapat et al., 2024; Angellotti et al., 2025).

Bromoform acts as a structural analog of methylated substrates involved in methane synthesis. It binds to the cobalt core of vitamin B₁₂, disrupting methyl group transfer by competing with endogenous cofactors. This inhibits the corrinoid iron-sulfur protein complex essential for the early stages of methanogenesis (Eason & Fennessy, 2025). In addition, bromoform directly targets the MCR enzyme by irreversibly binding to its nickel-based active site. This interaction results in a conformational blockade that prevents the final catalytic conversion of methyl-coenzyme M into methane. Unlike electron transport inhibitors that interrupt later metabolic stages, the halogenated compounds in *Asparagopsis* exert their effects at both early and terminal stages of the methanogenic pathway (Ungerfeld & Pitta, 2024; Eason & Fennessy, 2025).

Methane emissions in dairy and beef production systems are significantly reduced by the use of *Asparagopsis* as a feed additive. Still, there are obstacles in the way of attaining steady effectiveness in real-world scenarios. Because of microbial activity and environmental factors like UV exposure, the active compounds in the rumen break down quickly, making encapsulation strategies necessary to improve bioavailability. With pH-responsive or sustained release made possible by encapsulation using lipid-based or biopolymer systems, extended activity in the anaerobic rumen environment is guaranteed (Hodge et al., 2024; Angellotti et al., 2025).

The ability of *Asparagopsis* species to suppress methane is also influenced by biological variability. The concentration and stability of bioactive compounds are influenced by a number of factors, including the growing environment, seasonal variations, and genetic variations among strains. Research has concentrated on creating high-yielding cultivars and refining cultivation methods using regulated aquaculture systems in order to address this. When incorporated into multitrophic aquaculture networks, these systems not only promote biomass production but also aid in the provision of coastal ecosystem services like nitrogen uptake and eutrophication control (Cheong et al., 2024; Resetarits et al., 2024).

*Asparagopsis* has a high potential for mitigation, but its use must also take environmental sustainability, animal health, and food safety into consideration. Careful observation and dietary balancing are necessary due to residual halogenated compounds in animal products, iodine buildup, and potential effects on animal physiology, such as changes in volatile fatty acid profiles. Methane suppression, for instance, can change the way that fermentation proceeds, lowering the production of acetate and affecting the composition of milk. To preserve the quality of the final product, this may require additional nutritional changes (Goswami et al., 2024; Indugu et al., 2024).

*Asparagopsis* supplementation has been linked to changes in rumen microbial populations at the microbiome level, favoring hydrogen-utilizing taxa that use excess hydrogen to produce propionate instead of methane. Better feed energy utilization is supported by this redirection. The necessity for long-term stewardship is highlighted by new worries regarding microbial adaptation, such as the emergence of resistance pathways through gene transfer or the enzymatic breakdown of bromoform. To avoid resistance and improve mitigation synergy through orthogonal modes of action, tactics like alternating inhibitors or combining seaweed with other methane suppressants, like 3-NOP, are being researched (Indugu et al., 2024; Liu et al., 2024).

Additionally, customized dosing plans are being made possible by developments in precision feeding and herd-specific microbial profiling technologies. By reducing hazards like excessive iodine consumption or microbial disruption, these instruments aid in optimizing methane suppression. Post-harvest processing innovations like selective biofiltration and low-energy drying methods are enhancing the scalability and environmental impact of additives derived from *Asparagopsis* (del Prado et al., 2024).

*Asparagopsis* species provide a strong, biologically active way to reduce methane emissions when incorporated into all-encompassing management and nutrition plans. However, a thorough grasp of host nutrition, compound stability, rumen microbiology, and production system dynamics is necessary for their efficacy. To reach their full potential in climate-smart livestock production systems, further development in formulation, delivery, and regulatory harmonization will be necessary (Eason & Fennessy, 2023; Wanapat et al., 2024).

#### 3.1.3 Nitrate supplementation

As a competitive electron sink in rumen microbial metabolism, nitrate supplementation offers a promising approach to mitigating enteric methane. In order to successfully divert hydrogen away from methanogenic archaea, nitrate-reducing bacteria (NRB) in the rumen use nitrate as a terminal electron acceptor in dissimilatory nitrate reduction pathways. Through outcompeting methanogens for available reductants, this redirection prevents carbon dioxide from being reduced to methane. The process is carried out by specialized microbial taxa using enzymes that contain molybdenum and cytochrome. It starts with the conversion of nitrate to nitrite and then to ammonia (Bharanidharan et al., 2024; Tanaka et al., 2024).

Numerous variables, such as the physiological traits of the host species, the structure of the rumen microbiome, and the makeup of the basal diet, affect how well nitrate inhibits methane. While starch-rich diets may lessen the effects of nitrate by favoring fermentation pathways that produce less free hydrogen, high-fiber diets typically increase its effectiveness due to prolonged rumen retention and increased hydrogen generation. Crucially, by favoring nitrate-reducing populations, nitrate supplementation affects microbial ecology and can change rumen fermentation toward more energetically advantageous end products like propionate, improving feed efficiency (Ni et al., 2025; Pepeta et al., 2024).

Despite its potential for mitigation, nitrate has a known toxicity risk because it can form nitrite, which can be absorbed into the bloodstream and cause methemoglobinemia in ruminants if it is not quickly reduced. Due to this difficulty, ruminal microbial communities must be gradually conditioned to increase their capacity for nitrite reduction throughout a carefully monitored adaptation period. In order to postpone nitrate availability to posterior rumen regions, where detoxifying microbes are more active, encapsulation technologies and controlled-release delivery systems have been developed. By guaranteeing that nitrite is effectively metabolized prior to systemic absorption, these techniques increase safety (Miranda et al., 2024; Rendón-Correa et al., 2024).

Co-supplements like sulfur or trace minerals are used in further refinements because they improve nitrite detoxification and alter enzyme kinetics without materially impairing methane mitigation. However, overall hydrogen redirection efficiency may be decreased by interactions between competing microbial pathways, especially those involving bacteria that reduce sulfate or methanogens that use formate. The long-term sustainability of suppression is limited when methanogens adjust to nitrate-supplemented environments by establishing syntrophic associations with nitrate reducers and recycling hydrogen through intermediary compounds like formate (Giangeri et al., 2024; Zuo et al., 2024).

Environmental factors need to be taken into account in addition to the rumen. Nitrous oxide emissions from manure and nitrate seeping into groundwater are two examples of downstream nitrogen losses that can be caused by residual nitrate and its transformation products. If manure management techniques are not used concurrently, these indirect effects could cancel out the desired climate benefits. By enhancing nutrient retention and lowering secondary emissions, additives like biochar and integration with anaerobic digestion systems provide workable solutions for minimizing these trade-offs (Haq et al., 2024; Symeon et al., 2025).

More accurate and tailor-made nitrate supplementation regimens are possible thanks to the development of microbial genomics, metagenomic biomarkers, and biosensing technologies. These tools make it easier to optimize dosing plans based on microbial profiles, environmental factors, diet composition, and host genetics. Adaptive management of nitrate supplementation is now possible to maintain efficacy while guaranteeing animal health and environmental compliance, thanks to decision-support platforms that integrate real-time rumen analytics, weather data, and nutritional inputs (Guan, 2024; Yang et al., 2024a).

Novel strategies are also investigating how nitrate and other methane inhibitors work in concert. Nitrate can improve the effectiveness of combination therapies by scavenging leftover hydrogen that evades the action of inhibitors that target different points in the methanogenesis pathway. These strategies, however, need to integrate with dynamic dosing technologies and reliable safety monitoring systems in order to balance increased methane suppression with increased risks of nitrite accumulation (Tanaka et al., 2024; Zuo et al., 2024).

To close nitrogen loops in livestock systems, circular nutrient strategies are being investigated in addition to dietary interventions. These include using electrochemical and bioelectrochemical systems to recover nitrate from manure effluents, which not only lessens environmental losses but also offers a sustainable source of nitrate for future use. Their role in the shift to climate-smart, resource-efficient livestock production is further supported by the integration of nitrate mitigation techniques into larger sustainability frameworks, such as carbon credit systems and nitrogen offset programs (González et al., 2024; Moeller et al., 2024; Vingerhoets, 2024).

In conclusion, nitrate supplementation provides a flexible, biologically based method of mitigating methane that can be successfully incorporated into a variety of livestock systems. Its success hinges on the careful handling of dietary interactions, environmental trade-offs, toxicity risks, and microbiome dynamics—all of which are being addressed more and more through interdisciplinary research and innovation in microbial engineering, delivery systems, and decision-support tools (Bharanidharan et al., 2024; Patel & Pata, 2024).

### 3.2 Microbial modulators

The metabolic interactions between bacteria, archaea, protozoa, and fungi that make up the complex and dynamic rumen microbiome are essential to both ruminant productivity and greenhouse gas emissions. Methanogenic archaea are essential to the synthesis of enteric methane because they act as hydrogen sinks during anaerobic fermentation, among other functions. By rerouting hydrogen flux away from methanogenesis and toward alternate electron acceptors or syntrophic microbial interactions, microbial modulators seek to change this basic pathway. By doing this, these tactics aim to rewire the underlying microbial architecture in favor of lower-emission phenotypes rather than just suppress methane output (Pepeta et al., 2024; McAllister et al., 2025).

Microbial modulation uses biological specificity to create long-lasting changes in rumen ecology, in contrast to broad-spectrum chemical inhibitors. These interventions reduce collateral disruption to non-target microbial populations by focusing on specific biochemical pathways and methanogen-specific structural elements, such as coenzyme-mediated methyl transfers and non-peptidoglycan cell wall features. Vaccination against the surface proteins of important methanogenic taxa is one strategy being developed; it stimulates host immune responses that suppress target organisms without upsetting beneficial commensals. Probiotic-based tactics are also being researched, where specific bacterial strains are added to outcompete or directly oppose methanogens through metabolic pathways that consume hydrogen or metabolites that disrupt membranes (Chen et al., 2024; Jeong et al., 2024).

Interactions between the host and microbiota affect the effectiveness of microbial modulators. It has been demonstrated that variations in the host's genetic makeup, including immune-regulatory and salivary characteristics, are associated with variations in the composition of the microbial community and methane yield. The development of integrated breeding and microbial management strategies, in which targeted microbiome interventions are complemented by selection for desirable host genotypes, is supported by these findings. To prevent unforeseen consequences, caution must be exercised. Animal performance and feed conversion efficiency may be impacted by excessive hydrogen accumulation brought on by indiscriminate methanogen suppression, which may also inhibit cellulolytic bacteria and hinder fiber digestion (Marcos et al., 2024; Waters et al., 2024).

Emerging methods emphasize accuracy and flexibility to reduce such risks. While synthetic microbial consortia and engineered strains are being developed to control hydrogen utilization, bacteriophage-based methods provide species-specific targeting of methanogens through selective lysis. To preserve fermentative balance and maximize energy recovery, these systems may express different hydrogenase enzymes or divert reducing equivalents toward the synthesis of short-chain fatty acids. Additionally, synthetic biology makes it possible to create designer microorganisms that can modify metabolite profiles to favor less emissive fermentation pathways or produce anti-methanogenic peptides (Ramasamy & Pakshirajan, 2024; Tanaka et al., 2024).

Natural variation in rumen microbial communities across breeds, diets, and geographic contexts complicates the scalability and robustness of these interventions. Significant variations in methanogen prevalence and susceptibility have been found in global surveys of rumen microbiota, indicating that no one approach is always successful. In order to accommodate different microbial ecologies, rumen-retentive delivery systems, feed-grade probiotics, and slow-release formulations, combinatorial and locally tailored approaches are becoming more and more popular (Ramasamy & Pakshirajan, 2024; Subramanian, 2024; Tanaka et al., 2024).

Probiotic classification schemes and new additive approval processes are two examples of how the regulatory environment is progressively changing to support microbiome-based methane mitigation technologies. However, overcoming microbial adaptation and resilience remains crucial for long-term field effectiveness. In order to guide the improvement of dosage, delivery, and microbial strain selection, as well as to predict microbiome responses to intervention, tools like machine learning and metatranscriptomic profiling are now being used (D’Urso & Broccolo, 2024; Sabaria, 2024).

Integrating microbial modulators with wider animal husbandry systems is a milestone in climate-smart livestock management. Precision agriculture technologies such as automatic dosage units and *in vitro* bio-sensing allow real-time monitoring and adaptive control of microbial interventions. This makes it easier to manage methane dynamically, adapting to individual animals, environmental conditions, and production objectives. With advances in research, strategic management of the rumen microbiome - guided by multi-omics, host genetic profiling, and digital platforms - will be increasingly central in efforts to reduce emissions and maintain productivity (Ni et al., 2025; O’Connor et al., 2024; Zhao et al., 2024).

The successful application of microbial modulators will require both technological progress and an enabling socio-economic environment. These consist of regulatory protections to guarantee the efficacy and safety of microbial products, incentive systems like carbon credits, and education and outreach initiatives. As the field of rumen microbiome engineering advances, interdisciplinary cooperation and a dedication to ethical innovation in the interest of sustainable livestock systems must drive its application.

#### 3.2.1 Probiotics and direct-fed microbials

Direct-fed microbials (DFMs) and probiotics are two well-known microbial modulation techniques that selectively alter rumen fermentation pathways to reduce enteric methane emissions. Comprising live or inactivated microorganisms, these microbial additives work using metabolic redirection, niche displacement, and competitive exclusion. Their application stems from the ecological modulation principle, which aims to improve rumen health and fermentation efficiency by influencing the rumen microbial community toward compositions and functions that are less favorable to methanogenesis (Abd El Tawab et al., 2024; Silva et al., 2024).

Lactic acid-producing bacteria, especially those belonging to the *Lactobacillus* genus, are present in many DFMs. Lactate and short-chain fatty acids like acetate and propionate are produced when these organisms ferment water-soluble carbohydrates, mostly through the phosphoketolase and acrylate pathways. In addition to diverting metabolic hydrogen away from methane formation and toward the production of alternative end products like propionate, this fermentation pattern lowers ruminal pH to levels that are less than ideal for methanogenic archaea. This hydrogen redirection helps the host produce energy-producing volatile fatty acids while reducing net methane emissions (Ghiotto et al., 2024; Narasimha et al., 2024).

DFMs based on yeast, particularly those containing strains of *Saccharomyces cerevisiae*, have complementary functions. These include scavenging leftover oxygen, promoting the growth of fibrolytic bacteria that break down fiber, and stabilizing anaerobic conditions in the rumen. Yeasts can also alter the ratio of acetate to propionate, which further lowers the amount of hydrogen available to methanogens and encourages more effective fermentation. Adding yeast cultures has also been associated with better rumen stability and nutrient digestibility, especially during times of dietary stress or transition (Chen et al., 2024; Guo et al., 2024).

The application of antimicrobial metabolites generated by particular probiotic strains is a noteworthy advancement in this area. For example, reuterin, a substance secreted by some species of *Limosilactobacillus*, targets surface-layer proteins and interferes with vital coenzyme systems involved in methane synthesis, compromising the integrity of the methanogen cell membrane. Similarly, through interactions with cell wall components specific to archaea, bacteriocin-producing bacteria, such as modified strains of *Bacillus subtilis*, have demonstrated species-specific inhibitory effects against methanogens (Lathief & Manjusha, 2024; Park et al., 2025).

The efficacy of DFMs and probiotics in commercial livestock operations is still debatable, despite encouraging findings in controlled trials. Environmental conditions, animal species, the composition of the basal diet, and the structure of the current microbial community all affect efficacy. For example, if high-starch diets are not sufficiently buffered, they may make animals more susceptible to lactate buildup and rumen acidosis. Innovations like cryoprotectant-assisted lyophilization and microencapsulation employing biopolymers are being developed to protect probiotic viability through the gastrointestinal tract and guarantee retention in the reticulorumen in order to improve microbial survival and targeted delivery (Ghimire et al., 2024; Wu et al., 2024a).

The creation and acceptance of DFMs are also influenced by regulatory factors. Tough safety assessments, such as toxicological evaluations and environmental risk analyses, are part of the approval procedures for novel or genetically modified microbial strains. Because of this, commercialization schedules may be prolonged, and market access may be limited, especially for strains that have been modified to express enzymes or antimicrobial peptides that specifically target methanogenic pathways (Shams et al., 2024).

Inconsistent results, which are frequently ascribed to differences in host genetics and microbial responsiveness, continue to restrict the on-farm adoption of DFMs. Precision livestock management developments, however, are starting to address this issue. Metagenomic profiling and biomarker-based algorithms are examples of emerging tools that can be used to customize DFM formulations and dosage to the unique microbial ecology of each herd. By preserving animal health and production efficiency, these precision methods seek to maximize methane mitigation (del Prado et al., 2024; Zhao et al., 2024). Additionally, by specifically stimulating hydrogen-consuming microbial populations, probiotic-prebiotic synergistic strategies, like fermentable oligosaccharides, have shown increased efficacy. These combinations may result in additive or even synergistic decreases in methane emission when used with other methane inhibitors, such as 3-NOP (Hassen et al., 2024; Ni et al., 2025).

Future advancements in DFM development are likely to involve synthetic biology and phage-based technologies. Novel approaches to microbial intervention include phage-DFM conjugates intended to inhibit methanogen resistance mechanisms and engineered probiotic strains with improved fiber-degrading and hydrogen uptake efficiency. Probiotics and DFMs are positioned to play a key role in sustainable livestock systems as these technologies advance because they are multipurpose tools that promote animal health, nutrient efficiency, and environmental stewardship in addition to acting as emission mitigants (Kim et al., 2024; Nazir et al., 2024).

#### 3.2.2 Vaccines targeting methanogens

Methanogenic archaea-targeting vaccines stimulate the host animal's immune system to produce particular antibodies that inhibit methanogen activity in the rumen, providing a promising immunological strategy to lower enteric methane emissions. Finding conserved, methanogen-specific antigens that are essential for methane biosynthesis or cellular upkeep is the foundation of this tactic. Adhesion factors, structural surface-layer proteins (SLPs), and important enzymatic elements of the methanogenesis pathway, such as heterodisulfide reductase (Hdr) and methyl-coenzyme M reductase (Mcr), are examples of candidate antigens. These molecules' functional significance and taxonomic specificity make them immunogenic targets (Garcia-Ascolani et al., 2024; Muntari et al., 2025). These antigens have been identified across dominant methanogen genera thanks to advancements in metagenomics and structural proteomics. Subunit vaccine formulations are intended to stimulate both mucosal and systemic immune responses; they frequently include adjuvants and recombinantly expressed protein fragments. Saliva is thought to be the route by which systemically produced or mucosally secreted antibodies enter the rumen. There, they attach to specific targets on the surfaces of methanogen cells or enzymatic structures, either blocking their function or causing immune-mediated clearance (Burlet et al., 2024; Kocabiyik et al., 2024).

One significant development has been the use of methanogen-specific epitopes, such as those obtained from cell walls containing pseudomurein, which enable the selective targeting of methanogens without negatively impacting non-archaea members of the rumen microbiota. However, the effectiveness of vaccines against various ruminant species and geographical areas is hampered by the antigenic diversity among methanogen strains. Chimeric antigen constructs that combine conserved regions from several methanogen clades have been created in order to get around this. The goal of these multivalent antigens is to decrease the possibility of immune evasion and increase immune coverage (Lupo et al., 2024; Xie, 2024).

For methanogen-targeted vaccines to be effective, adjuvant selection is essential. Novel delivery systems, like platforms based on nanoparticles, have either replaced or supplemented traditional adjuvants. These systems improve mucosal immune activation and antigen presentation. In order to target methanogens at mucosal surfaces, these formulations are intended to raise antibody titers, especially secretory IgA (Rashidi et al., 2024; Valiveti et al., 2024).

One of the most important practical factors in field deployment is still the delivery method. Regarding immunogenicity, labor requirements, and consistency of rumen antibody concentrations, subcutaneous, intramuscular, and intranasal administration routes each have unique benefits and drawbacks. Furthermore, developments in oral vaccine technology, such as transgenic forage crops that express methanogen antigens, are being investigated as a scalable and affordable delivery method, especially for application in large-scale grazing systems (Bouazzaoui & Abdellatif, 2024; Zhong et al., 2024). Vaccine responsiveness is also influenced by host genetic variation. Enhanced vaccine efficacy has been linked to specific immune-related gene variants, especially those related to antigen presentation and antibody isotype expression. This creates opportunities for integrated breeding approaches that favor animals that respond better to vaccines that target methanogens (Cohen et al., 2024). There are still difficulties in spite of these developments. Methanogens may develop defenses against immune detection, like changing the way their surface proteins are glycosylated. Antigenic updates may therefore be necessary regularly, akin to methods employed in human influenza vaccination. Computational tools, such as machine learning algorithms, are being developed to predict epitope drift and direct vaccine design in order to account for such variation (Shi et al., 2024a).

Additionally, efforts are being made to create global methanogen antigen data repositories, which can facilitate logical vaccine design and encourage uniformity among research projects. As vaccine technologies advance, focus is shifting to controlled-release delivery methods that can sustain effective antibody levels for prolonged periods of time, like rumen-retentive capsules. Methanogen vaccines are positioned as a promising and potentially scalable solution for methane mitigation within climate-smart livestock systems, thanks to these developments as well as advancements in genomics, immunology, and delivery science (Zhao et al., 2024; Xu, 2024).

#### 3.2.3 Phage therapy applications

A new biological approach in livestock systems is phage therapy, which uses bacteriophages—viruses that infect and lyse particular bacterial hosts—to provide a targeted method of pathogen control. In order to cause cell lysis and population suppression, these viral agents bind to bacterial surface receptors, inject their genetic material, and take over the host's replication machinery. Phage therapy, in contrast to broad-spectrum antibiotics, is intrinsically specific, enabling the selective eradication of harmful bacteria while maintaining commensal microbiota and reducing the ecological disturbance frequently linked to the use of traditional antibiotics (Choi et al., 2024; Culqui Molina et al., 2024).

Growing antimicrobial resistance and consumer demand for residue-free, environmentally friendly products have sparked interest in the use of phage therapy in livestock production. Phages are being tested for their ability to lower bacterial burdens in a variety of species and can be applied topically, orally, or through environmental exposure through feed additives or water. Because of its limited host range, phage therapy has a significant advantage in that it has little collateral effect on beneficial microbial communities, maintaining gastrointestinal homeostasis and promoting host health (Alsayed & Permana, 2024; Choi et al., 2024).

However, issues with phage delivery, host-pathogen dynamics, and phage survivability make phage therapy difficult to apply. Protective delivery systems are necessary to maintain phage viability through the upper gastrointestinal tract, especially in acidic environments. To improve gastrointestinal stability and guarantee phage release at target sites, methods like microencapsulation in lipid or alginate matrices have been developed. Furthermore, the quick co-evolution of bacterial defenses, such as receptor mutations and CRISPR-Cas immune systems, is intimately linked to phage efficacy. Phage cocktails need to be updated frequently to combat these adaptations, and ongoing efficacy requires real-time pathogen surveillance (Cui et al., 2024; Kou et al., 2024).

Phage-based methods are becoming more useful thanks to creative tactics. For example, to break up biofilms and lessen pathogen persistence, phage-derived enzymes such as endolysins and depolymerases are being added to feed or housing environments. Furthermore, phages that can deliver functional genes that deactivate antibiotic resistance mechanisms can be engineered through synthetic biology, providing a dual function in pathogen control and resistance reversal. These developments reinforce the function of phage therapy in larger antimicrobial stewardship programs as well as in the prevention of disease (Grygiel et al., 2024; Peng et al., 2024b).

Phage therapy has shown promise in working in tandem with probiotic and prebiotic supplementation in integrated health and production systems. It has been demonstrated that combinatorial methods improve growth performance metrics, alter microbial ecology, and strengthen gut mucosal immunity. When animals are most vulnerable to enteric infections, such as during weaning or dietary transitions, such multifaceted interventions may be especially helpful (Jiang et al., 2024a).

Scalability is still an issue despite encouraging advancements. To maintain viral infectivity, phage production necessitates specialized infrastructure for cold-chain distribution, sterile fermentation, and quality control. Because phages are classified differently in different jurisdictions as biologics, feed additives, or therapeutic agents, regulatory harmonization presents additional challenges that result in inconsistent approval and trade compliance. To close these gaps, industry stakeholders, academic institutions, and regulatory bodies must work together to standardize safety evaluations and open up international markets (Fowoyo, 2024; Kaistha et al., 2025).

Phage therapy's accuracy is being further improved by technological advancements. In order to enable proactive and flexible disease management, machine learning models are being used to predict bacterial outbreaks and optimize phage dosing schedules. The development of regionally specific phage libraries that represent local pathogen ecologies is made easier by the speed at which novel phages are being discovered and characterized from environmental reservoirs like soil, manure, and rumen fluid, thanks to advancements in metagenomic sequencing (Shanmugha Mary et al., 2023; Rivera-Orellana et al., 2024).

Phage therapy provides a versatile and genetically programmable solution in line with the One Health tenets, as climate change modifies pathogen distribution and disease dynamics. Phage-based interventions facilitate the shift to more resilient and sustainable livestock systems that lessen dependency on antibiotics and lower the risks of emerging infectious diseases by combining environmental, animal, and human health viewpoints (Siyanbola et al., 2024).

### 3.3 Natural compounds

Natural compounds have become a compelling way to reduce GHG emissions in the pursuit of sustainable livestock production, especially methane produced by enteric fermentation in ruminants. These substances offer a biologically active and environmentally friendly way to modify rumen fermentation without the negative effects sometimes associated with synthetic additives. They include a wide range of bioactive substances, including essential oils, polyphenols, saponins, plant secondary metabolites, and unsaturated fatty acids. Natural compounds are frequently acknowledged as safe and appropriate for use in livestock feed, especially in organic and natural production systems, in contrast to conventional inhibitors that might jeopardize animal health or necessitate stringent regulatory approval (Alabi et al., 2024; Lambo et al., 2024; Matabane et al., 2024).

Targeting important methanogenic pathways and microbial populations in the rumen is one of the main reasons for the increased interest in natural feed additives. Many substances derived from plants, for example, have specific antimicrobial properties that inhibit methanogens and protozoa, which are essential for the production of methane, without interfering with the fermentation process or the digestion of fiber. Furthermore, natural substances can divert ruminal hydrogen from methanogenesis to other metabolic sinks, like the synthesis of propionate, which helps the host animal use energy more efficiently while also lowering emissions. Reduced methane output and enhanced feed conversion are two advantages that have important ramifications for production economics and environmental sustainability (Abd El Tawab et al., 2024; Bature et al., 2024).

Furthermore, the use of natural compounds is consistent with the larger trends in consumer demand and policy focus on climate-smart, residue-free, and clean-label livestock products. Regulations prohibiting synthetic additives and antibiotic growth promoters have given natural alternatives a chance to gain traction in many areas, especially in North America and Europe. Simultaneously, producers are being encouraged to lower emissions through sustainability-driven value chains, carbon markets, and eco-certification initiatives, all of which acknowledge the potential contribution of feed-based mitigation techniques (Fonseca et al., 2024; Suganthi, 2024).

Notwithstanding these benefits, using natural compounds is still fraught with difficulties and complications. Depending on the botanical source, extraction technique, concentration, animal species, diet composition, and environmental factors, these additives' effectiveness can vary significantly. Furthermore, palatability and effective dosage levels are frequently traded off, which can affect feed intake and animal performance. The consistent implementation of these interventions across various production systems is further constrained by the absence of commercial-scale studies and standardized formulations. However, the accuracy and predictability of natural compound applications are steadily increasing due to developments in analytical chemistry, feed technology, and rumen microbiome research (Alem, 2024; Lambo et al., 2024).

In this regard, a crucial nexus between environmental science, sustainable agriculture, and animal nutrition is represented by the use of natural compounds as methane mitigation tools. The subsequent sections will examine distinct categories of natural additives, including tannins, essential oils, saponins, and lipid-rich feed ingredients, emphasizing their modes of action, species-specific effectiveness, and usefulness for inclusion in animal diets. These substances have enormous potential for supporting more general objectives in animal health, productivity, and sustainable intensification in addition to serving as a component of an integrated emissions reduction strategy (Abd El Tawab et al., 2024; Matabane et al., 2024).

#### 3.3.1 Tannins, essential oils, and saponins

In ruminant livestock systems, tannins, essential oils, and saponins have become important natural substances with a great deal of promise to reduce enteric methane emissions. These phytochemicals have strong biological activity in the rumen ecosystem and target methanogenesis through a number of microbial and biochemical pathways. They are primarily derived from a broad range of forage plants, shrubs, spices, and medicinal herbs. Tannins have arguably been the subject of the most research in this area. Tannins can be broadly divided into two groups: condensed tannins (CTs), which are polymers of flavan-3-ols, and hydrolyzable tannins (HTs), which are made of gallic or ellagic acid esters of glucose (Kadigi et al., 2024; Matabane et al., 2024). CTs, which are present in plants like the Acacia species. *Calliandra calothyrsus* and *Lotus corniculatus* are generally thought to be more effective at reducing methane because they are more stable in the rumen. Tannins suppress methane in a number of ways, including direct inhibition of methanogenic bacteria and protozoa, reduction of hydrogen availability through protein binding, reduction of the concentration of ruminal ammonia, and reduction of the total fermentation rate (Matabane et al., 2024). Some studies showed that dietary CT scanners could reduce methane production by 10–25%, although this is very dependent on the dose. However, their negative nutritional effects, such as reduced digestibility of fiber and protein at high concentrations, are a limitation. Therefore, to balance the mitigation benefits with the performance of the animals, an optimal rate of incorporation should be determined. The efficacy of tannins may also be affected by ruminant species, the composition of the basic diet, and the source and chemical composition of tannins (Foggi et al., 2024; Hassen et al., 2024).

An additional promising class of phytochemicals that reduce methane is essential oils (EOs). Thymol, carvacrol, eugenol, and allicin are among the active molecules found in these aromatic, volatile compounds, which are derived from plants like oregano, thyme, garlic, cinnamon, and clove. By interfering with rumen microbes' enzymatic systems, selectively inhibiting methanogens and protozoa, and disrupting the integrity of microbial membranes, EOs reduce the production of methane (Lambo et al., 2024; Matabane et al., 2024). Essential oils can change fermentation patterns to produce propionate instead of acetate and methane by modifying the rumen microbiome. Moreover, EOs may lessen protein deterioration and ammonia levels, which would further cut off the hydrogen supply to methanogens. However, because of things like volatility, rumen degradation, and animal adaptation, the effects of EOs are inconsistent and vary in *in vivo* settings. Excessive dosages may cause microbial resistance and reduce feed intake. Recent advancements in EO encapsulation, like emulsification or microencapsulation, are enhancing their targeted delivery and stability, expanding their suitability for use in agricultural settings. Despite their promise, the cost, regulatory approval, and standardization continue to be barriers to their broad commercial adoption (Foggy et al., 2024; Pangesti et al., 2024).

Plants like *Yucca schidigera, Quillaja saponaria*, and *Trigonella foenum-graecum* (fenugreek) are common sources of saponins, a third class of secondary metabolites with anti-methanogenic potential. Saponins have detergent-like qualities and can lyse protozoal membranes because of their structural makeup, which consists of a hydrophobic aglycone backbone joined to hydrophilic sugar chains (Mshary et al., 2024b). The main way that saponins reduce methane is by inhibiting rumen protozoa, which are important methanogen symbionts. As a result, there are fewer methanogens present and less hydrogen transfer. Additionally, saponins change microbial populations to favor cellulolytic bacteria and propionate producers, which encourages fermentation pathways that produce less methane. Their effectiveness is dose-sensitive, though, just like tannins and EOs. Effects might be negligible at low doses, but detrimental effects on microbial activity and nutrient digestibility could happen at high doses. Furthermore, saponins' biological activity and capacity to suppress methane are impacted by the significant variations in their chemical structures among plant sources. Microbial adaptation is another issue; over time, rumen microbes may become resistant to saponins, reducing their long-term potency (Lambo et al., 2024; Matabane et al., 2024; Mshary et al., 2024b).

When properly prepared and used, tannins, essential oils, and saponins provide practical and physiologically effective solutions for lowering methane emissions, despite certain drawbacks. Because of their multipurpose advantages and natural origin, they are especially well-suited for incorporation into organic and sustainable livestock systems. In addition to lowering methane, these substances frequently enhance nitrogen uptake, regulate immunological responses, and promote rumen health in general. Nevertheless, their implementation necessitates a more thorough comprehension of species-specific reactions, interactions with food ingredients, and long-term impacts on microbial ecology and productivity (Hassen et al., 2024; Matabane et al., 2024; Mshary et al., 2024b). The most effective plant sources, ideal inclusion rates, better delivery systems, and the assessment of additive or synergistic effects when combined are among the top research priorities. The role of these natural compounds in low-emission livestock systems is anticipated to grow considerably with advancements in rumen microbiome analysis, metabolomics, and feed processing technologies, making a significant contribution to the global goals for reducing greenhouse gas emissions (Bature et al., 2024).

#### 3.3.2 Lipid supplementation and oilseeds

Because of their biochemical characteristics and effects on rumen microbial dynamics, lipid supplementation and the use of oilseeds have become more studied and useful methods for reducing enteric methane emissions from ruminant livestock. Reduced methanogenesis in the rumen has been repeatedly linked to the consumption of dietary fats, especially unsaturated fatty acids. This reduction is mostly accomplished by a number of interconnected processes, such as the suppression of protozoa that produce hydrogen, the inhibition of methanogenic archaea, and the biohydrogenation process, which reroutes metabolic hydrogen away from methane formation pathways. Furthermore, rumen fermentation patterns frequently change as a result of lipid supplementation, favoring the production of propionate over acetate, which reduces the amount of hydrogen available for methanogens (Abd El Tawab et al., 2024; Giagnoni et al., 2024; Pepeta et al., 2024).

The kind of lipid has a significant impact on how well it reduces methane. Because of their greater affinity for hydrogen in the rumen, unsaturated fatty acids, particularly polyunsaturated fatty acids, or PUFAs, are more efficient than saturated fats. These include the well-researched omega-3 and omega-6 fatty acids present in oilseeds such as flaxseed, sunflower seed, cottonseed, and canola. In the rumen, these fatty acids go through a process called biohydrogenation, where they function as hydrogen sinks and reduce the amount of free hydrogen needed to produce methane. By rupturing the cell membranes of methanogens and protozoa, medium-chain fatty acids (MCFAs), like those found in coconut and palm kernel oils, directly harm these microorganisms and reduce the number of microbial populations that produce methane. Additionally, these fatty acids suppress cellulolytic bacteria, which has an indirect effect on the digestion of fiber and the subsequent generation of hydrogen. Despite their effectiveness, MCFA use needs to be closely monitored because it can lower feed intake and overall digestibility, particularly in diets high in fiber (de Ondarza et al., 2024; Llonch et al., 2024; Yang et al., 2024b).

As a more rumen-friendly substitute for free oils, whole oilseeds are frequently utilized. They provide a more gradual release of lipids, reducing sudden disturbances to rumen microbial populations and largely maintaining the digestion of fiber. Over time, the rumen microbiota can adapt, and a more gradual fermentation is made possible by the fiber and protein matrix of the seed coat, which may have long-lasting mitigating effects. Additionally, oilseed meals, which are byproducts of oil extraction, contain leftover fats that help suppress methane while also offering ruminant diets valuable protein (Ahmed et al., 2024a; Kokić et al., 2024).

The inclusion rate is a crucial factor to take into account when using lipid supplementation. When added as free oils, diets with more than 6–7% total fat (on a dry matter basis) run the risk of impairing fiber intake and digestion. If not carefully balanced with forage quality and overall diet formulation, this can hurt production parameters. Supplements with protected fat (e.g., calcium salts of fatty acids) have been created to deliver lipids post-ruminally, avoiding rumen fermentation. This minimizes adverse interactions in the rumen while maintaining the benefits of energy supply and methane mitigation. These are more expensive, though, and might not be available in every production system (Hussein et al., 2024; Shpirer et al., 2024).

Beyond lowering methane, lipid supplementation has shown other advantages from the standpoint of animal production. Increased dietary fat content in dairy cows improves the diet's energy density, which is especially beneficial in the early stages of lactation when the cows' energy needs are high. Furthermore, by raising the concentrations of advantageous unsaturated fatty acids like conjugated linoleic acid (CLA) and omega-3 fatty acids, lipid supplementation can positively change the fatty acid profile of milk and meat, enhancing the nutritional value of animal products. These enhancements not only appeal to consumers who are health-conscious, but they also create opportunities for the marketing of livestock products with added value that have positive effects on the environment and human health (Giagnoni et al., 2024; Lashkari et al., 2024; Xiao et al., 2024).

Precision lipid feeding, in which lipid supplementation is customized to animal-specific responses, production stage, and microbial ecology, has been the focus of recent research efforts. By shedding light on how microorganisms adapt to long-term lipid feeding, techniques like metagenomics and rumen transcriptomics have helped to improve supplementation strategies that maintain methane reduction without causing negative side effects. In an attempt to produce synergistic effects, lipids are also being combined with other methane inhibitors or rumen modifiers, such as tannins, 3-NOP, or essential oils. By lowering the inclusion rates of each additive, these integrated strategies may maximize mitigation potential while minimizing any potential negative side effects (de Ondarza et al., 2024; Qin et al., 2024).

The broad use of lipid supplements necessitates a fair evaluation of the trade-offs between the economy and the environment from the perspective of sustainability. Even though oilseeds can be grown domestically in many places, their use needs to be weighed against the environmental impact of oilseed production, land use, and competition from food and feed. Sustainability and circularity may be improved by using oilseed processing byproducts or by incorporating oilseed crops into rotational systems with cereals and legumes. Furthermore, to ascertain the cost-effectiveness and return on investment for farmers implementing lipid-based mitigation strategies, region-specific economic analyses are crucial (Arru et al., 2024; Murphy, 2024).

Lipid supplementation and the thoughtful application of oilseeds provide a nutritionally sound, scientifically supported, and reasonably feasible way to reduce enteric methane emissions in ruminants. Dietary lipids can drastically change rumen fermentation in favor of reduced methane production through a variety of processes, from hydrogen redirection to microbial inhibition. However, careful diet formulation, consideration of animal productivity and health, economic viability, and alignment with more general sustainability goals are necessary for their successful implementation. The effectiveness and applicability of this mitigation strategy in international livestock systems will be further improved by ongoing research and innovation in this field, especially in the areas of lipid encapsulation technologies, precision feeding instruments, and microbiome-lipid interaction modeling.

## 4 Genetic and breeding approaches

By combining developments in genomics, bioinformatics, and reproductive biotechnology, genetic and breeding methods offer a revolutionary axis in the quest for sustainable livestock production, improving animal health, productivity, and environmental resilience. Breeders can now choose or engineer livestock with better performance, lowering dependency on resource-intensive inputs and pharmaceutical interventions, thanks to the clarification of the genetic foundations governing complex traits like disease resistance, feed efficiency, thermal tolerance, and methane emission profiles (Arya et al., 2024; Shoyombo et al., 2024).

With the combination of whole-genome sequencing and marker-assisted selection, traditional selection techniques, once constrained by phenotypic assessment and long generational intervals, have changed. With the use of these tools, important alleles and genomic regions linked to production and adaptive traits in a variety of livestock species can be identified. This change has been expedited by the use of gene-editing platforms like CRISPR-Cas systems, which allow for precise alterations that can improve reproductive performance, improve carcass composition, or confer resistance to infectious diseases. Simultaneously, research in developmental programming shows how maternal nutrition and prenatal environmental factors can epigenetically affect offspring gene expression, giving breeding strategies an extra level of complexity and potential (Doultani et al., 2024; Ghavi Hossein-Zadeh, 2024).

Predictive modeling of polygenic traits is becoming possible with the rise of computational genomics, specifically the application of machine learning algorithms to high-resolution genotypic datasets. This advancement makes it easier to identify people who possess advantageous genetic architectures for productivity, welfare, and health, even when those traits are regulated by multiple loci with negligible effects. The functions of non-coding regulatory regions in regulating host immune responses and environmental adaptability are becoming clearer thanks to extensive collaborative efforts, such as those aimed at annotating functional aspects of animal genomes (El-Attar & Elmashad, 2024; Ghavi Hossein-Zadeh, 2025; Zhang et al., 2025a).

To counteract the loss of biodiversity in commercial breeds that are subjected to rigorous selection, breeding programs are increasingly integrating genetic conservation initiatives. Stakeholders use assisted reproductive techniques, somatic cell repositories, and cryopreservation technologies to preserve and restore rare or heritage genotypes that may have adaptive value for future challenges. These conservation genomics efforts also align with the broader objectives of maintaining ecological balance and ensuring the long-term genetic resilience of livestock populations (Gao et al., 2024; Purdy, 2024).

Research is moving closer to creating artificial chromosomes that carry modular genetic constructs for stacked traits, like enhanced nutrient utilization and resistance to multiple diseases, as synthetic biology gains popularity. Although these technologies have the potential to completely transform livestock breeding, they also bring up issues with traceability, public opinion, and fair access, especially in areas with weak infrastructure or inconsistent regulations. Concerns about germline modification and its ecological effects, among other ethical issues related to gene editing, continue to influence international policy discussions and the rate of implementation (Fu & Shen, 2024; Ilyas et al., 2024). Additionally, efforts are underway to improve lifetime efficiency metrics about emissions and resource use, optimize harvest timing, and refine management decisions using epigenetic biomarkers, such as those based on DNA methylation signatures. Multidisciplinary research initiatives are starting to measure how genetic advancements result in system-level advantages such as decreased use of antibiotics, increased nutrient efficiency, and better contributions to food and nutritional security (Ahtisham & Obaid, 2025).

Regardless of their great potential, genetic and breeding technologies for methane reduction need to be viewed through a long-term time context since significant population-level decreases in the levels of methane emissions are usually obtained over many generations, not in brief implementation cycles. The intervention of low-methane phenotypes is furthermore better suited to decadal mitigation targets and should not be a substitute for short-term interventions (Ghavi Hossein-Zadeh, 2022; Assan, 2025). In addition, the reduction of emissions can be achieved at the expense of other main production and functional characteristics, such as fertility, health resilience, growth performance, and product quality. Thin selection goals may unintentionally result in reduced system productivity or robustness of animals without caution in constructing the index and maximizing the traits of multiple traits (Ghavi Hossein-Zadeh, 2022). Genetic diversity is of great importance because it is possible that the aggressive selection pressure of the traits related to emissions can lead to a decrease in adaptation capacity and leave organisms more vulnerable to new diseases or environmental challenges. These limitations emphasize the need to have balanced breeding goals that would incorporate emission reduction and productivity with the welfare and resilience characteristics (Shi et al., 2024b; Wrzecińska et al., 2024; Džermeikaitė et al., 2025).

Economic and infrastructural barriers have a significant impact on the practical aspect of genetic mitigation measures. The selection of low-emission phenotypes by genomics will necessitate a substantial investment in phenotyping systems, genotyping technologies, and data management systems, which can be easily available in highly industrialized systems but otherwise prohibitive in the context of smallholders or resource-constrained environments. These techniques can be made effective by having large and carefully curated datasets relating methane phenotypes and genomic data from a variety of environments, breeds, and management systems (Zhao et al., 2024; Assan, 2025). Recording systems in areas with fragmented or no recording systems can be adopted, causing further inequalities in production systems. These factors highlight why a scalable and cost-efficient solution to phenotyping is needed, and why international frameworks of data-sharing should be implemented to ensure that more people can access the benefits of genetic mitigation programs and that these benefits are distributed equally (Klingström et al., 2024; Navajas et al., 2025).

Genetic and breeding strategies are not useful as standalone solutions to the problems, but as part of an integrated mitigation strategy (Ghavi Hossein-Zadeh, 2022, 2023). The results of mitigation can be multiplied by synergies with dietary interventions, complementary management, new technologies, and reduced timelines can be achieved. As an example, genetic selection towards desirable rumen characteristics when used alongside specific nutritional interventions or decision-support tools in real time can be used to optimize emission reduction and production efficiency (Assan et al., 2025; Malyugina et al., 2025). Genomic technologies, reproductive biotechnologies, and predictive modeling (genome-enabled selection), improved reproductive dissemination techniques, and microbiome-informed breeding can all be promising approaches to hasten genetic gain without reducing diversity. Placing genetic solutions in an integrated systems framework helps to affirm their position as the enabling tools of sustainable, long-term mitigation, which will help to direct future research focus on integrated approaches that balance the environmental impact and production sustainability with economic viability (Alem & Aklilu, 2025; Džermeikaitė et al., 2025).

**Fig. 3:**
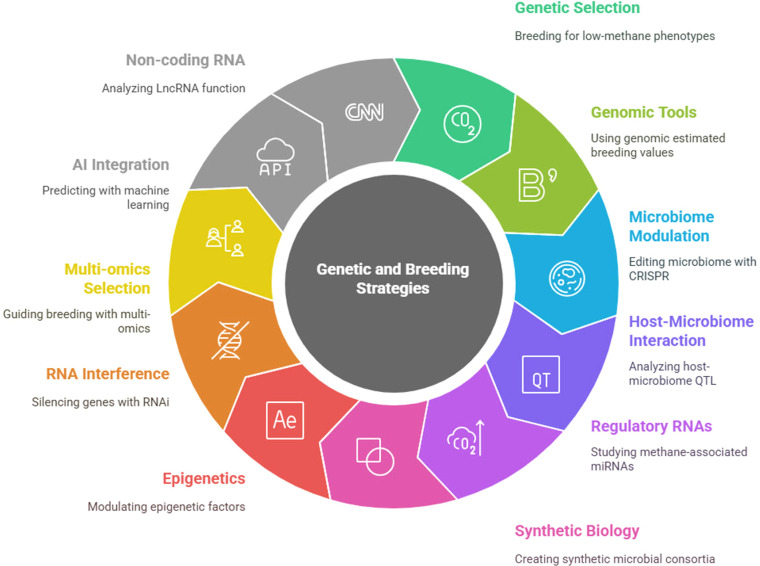
Genetic and breeding strategies for GHG mitigation in livestock.

In general, advancements in genetics and breeding are redefining the capacities of livestock systems to satisfy the rising demand for food derived from animals while also supporting the objectives of environmental sustainability and animal welfare. These technologies provide a route toward climate-smart animal agriculture that is both socially and scientifically responsible when combined with complementary tactics and backed by inclusive governance. An overview of genetic and breeding strategies for mitigating GHG emissions in livestock is shown in Table 3 and Fig. 3.

**Table 3:** An overview of genetic and breeding strategies for mitigating GHG emissions in livestock.

Category	Intervention	Mechanism	Efficacy (CH₄ reduction)	Advantages	Limitations	Recent references
Genetic selection	Low-methane phenotype breeding	Selecting animals with lower residual methane emissions	10–25%	Permanent, accumulative, compatible with other strategies	Requires phenotyping, low heritability	Ryan et al. (2025) Worku (2024)
Genomic tools	Genomic estimated breeding values	Genomic prediction for methane traits	10–30%	Early selection is possible. They are more precise	Data-intensive, cost of genotyping	Fresco et al. (2025) Alemu et al. (2025)
Microbiome modulation	CRISPR-based microbiome editing	Genome editing of rumen microbes to reduce methane	30–50% (projected)	Targeted intervention, long-term modulation	Regulatory, ethical concerns, rumen complexity	Mrutu et al. (2025) Malyugina et al. (2025)
Host-microbiome interaction	Host-microbiome QTL	Identifying genomic loci interacting with the rumen microbial community	10–20%	Novel trait insight. It supports selection	Complex data integration, low repeatability	Cardoso et al. (2025) Wang et al. (2024b)
Regulatory RNAs	Methane-associated miRNAs	Regulatory control over methane-linked gene networks	5–15% (projected)	Gene expression control, non-invasive biomarker potential	Limited knowledge, validation needed	Ricci et al. (2022) Cardoso et al. (2025)
Synthetic biology	Synthetic microbial consortia	Engineered microbial communities to suppress methanogens	30–60% (lab-scale)	Designed function, high specificity	Stability in rumen, regulatory hurdles	Tseten et al. (2025) Ni et al. (2025)
Epigenetics	Epigenetic modulation	Heritable expression changes via methylation, histone modification	10–20% (projected)	Rapid phenotypic shifts, reversible	Environmentally labile, poorly characterized	Hu et al. (2023) Morris and Bohannan (2024)
RNA interference	RNAi-based gene silencing	Targeted silencing of methanogenesis genes in microbes	20–40% (experimental)	Target specificity, transient expression	Delivery method challenges, off-target risks	Khan et al. (2024b) Gurgan (2025)
Multi-omics selection	Multi-omics-guided breeding	Integration of genomics, transcriptomics, metabolomics, and microbiome data	15–35% (synergistic)	Holistic trait understanding	High cost, bioinformatics demand	Guo et al. (2025) Zhao et al. (2024)
AI integration	Machine learning-assisted genomic prediction	AI-based prediction of methane traits using complex data	15–30%	Integrates big data, higher precision	Requires large, curated datasets	Altshuler et al. (2025b) Alemu et al. (2025)
Non-coding RNA	LncRNA function analysis	Regulation of methane traits via long non-coding RNAs	Under investigation	New regulatory targets, potential biomarkers	Poor annotation, exploratory phase	Hu et al. (2022)

### 4.1 Low-methane emission livestock breeding

A crucial element of climate-smart agriculture is breeding animals for reduced methane emissions, especially when it comes to addressing the environmental impact of ruminant enteric fermentation. Targeted interventions to lower greenhouse gas emissions without sacrificing productivity are now possible thanks to developments in genomics, phenotyping, and computational modeling that enable the identification and selection of animals with a genetic predisposition toward lower methane output (Holmes, 2024; Zhao et al., 2024).

This approach is based on the finding that ruminant methane production varies heritably, which can be measured using genomic selection techniques. Breeders can determine breeding values that represent an animal's propensity for methane production by comparing genetic markers with phenotypic data gathered from methane measurement technologies, such as respiration chambers, automated emission sensors, or milk-based spectroscopy. Multi-trait selection indices, which balance emission intensity with other economically significant traits like milk yield, growth rate, or reproductive efficiency, are increasingly incorporating these genomic estimated breeding values (EBVs) (Ghavi Hossein-Zadeh, 2022, 2023; Dressler et al., 2024).

Methane-related characteristics, such as those affecting feed efficiency, rumen microbial composition, and fermentation profiles, have been linked to a number of genomic regions. These loci present viable targets for selection initiatives that seek to change rumen fermentation in favor of pathways that decrease methanogens' access to hydrogen, thereby reducing methane production. Crucially, some genes have pleiotropic effects, meaning that selection for traits with higher production also unintentionally modifies metabolic processes in ways that inhibit the production of methane. The inclusion of methane traits in mainstream breeding objectives is supported by these two advantages (Domínguez-Castaño, 2024; Zhao et al., 2024).

Low-emission breeding programs are now even more scalable thanks to advanced phenotyping technologies. For example, non-invasive techniques that use biological sample infrared spectroscopy to estimate methane emissions offer a useful way to gather sizable datasets from a variety of herds. When paired with extensive genomic data, these phenotypic proxies improve selection model accuracy and hasten genetic advancement (Worku, 2024).

The impact of the host genome on microbial ecology introduces another level of complexity to ruminant systems, where microbial fermentation in the rumen is intimately related to methane production. There is evidence to support the idea that host genetic variation influences methanogenic archaea abundance and composition, providing yet another indirect selection mechanism. In order to gain a more thorough understanding of host-microbe interactions and their consequences for methane emissions, some breeding programs are starting to incorporate metagenomic data from rumen samples into genomic assessments (Marcos et al., 2024; Zhao et al., 2024).

Future interventions may also be possible with emerging gene-editing technologies. The potential of genome editing platforms, like CRISPR-Cas, to directly alter host genes that affect microbial substrate availability or rumen physiology and change the rumen environment in favor of lower-emission fermentation is being studied. Nonetheless, these methods bring up significant moral, legal, and public acceptance issues that differ greatly between jurisdictions (Agarwal et al., 2024).

The importance of low-emission genetics is starting to be reflected in economic incentives. Animals with proven methane efficiency are fetching higher prices in some market environments, especially those with carbon credit programs or sustainability certifications. The role of genetics in more comprehensive mitigation strategies is further supported by life cycle assessment models, which consistently demonstrate that genetically enhanced herds result in quantifiable decreases in greenhouse gas emissions per unit of food derived from animals (Shi et al., 2024b; Worku, 2024).

Harmonizing regulations is still difficult, particularly when it comes to aligning global standards for breeding value accreditation and standardizing methane measurement procedures. However, methane traits are increasingly being included by breeding organizations in composite sustainability indices, which is in line with international methane reduction pledges and other global policy commitments. This change reflects an increasing understanding of the necessity of designing livestock genomes for environmental resilience and global climate goals in addition to production efficiency (Esiri et al., 2024).

To sum up, breeding livestock with low methane emissions offers a financially sound and scientifically sound way to lessen the environmental effects of animal husbandry. These programs support a new paradigm in which genetic improvement is used to address productivity and planetary health by combining genomic tools, phenotypic innovations, and systems-level sustainability metrics.

### 4.2 CRISPR-based microbiome engineering

With the ability to precisely modify gut microbial communities for the purposes of improving nutrient utilization, inhibiting pathogenic organisms, and reducing methane emissions, CRISPR-based microbiome engineering is becoming a game-changer in livestock management. This technology uses programmable nucleases like CRISPR-Cas9 and Cas12a to edit the genomes of microorganisms with high specificity, changing metabolic processes and interspecies interactions in the intestinal or rumen environment (Chavan et al., 2024; Nazir et al., 2024).

CRISPR tools are used in methane mitigation strategies to interfere with important enzymatic pathways in methanogenic archaea. For instance, by directly interfering with the metabolic processes of dominant archaeal genera, silencing genes necessary for methanogenesis, such as those encoding the methyl-coenzyme M reductase, has demonstrated promise in lowering methane production. In order to efficiently transfer genes within the anaerobic confines of the gastrointestinal tract, these edits are frequently administered using engineered vectors like lipid-based nanoparticles or bacteriophages (Du et al., 2024a; Gropp et al., 2024).

CRISPR-mediated gene insertion methods have been investigated to improve the functional ability of fermentative and fibrolytic bacteria in addition to emission control. It may be possible to increase feed conversion efficiency while preserving rumen stability by introducing genes encoding enzymes such as beta-glucanases into fiber-degrading microbes. Without requiring extra dietary inputs or jeopardizing gut health, these methods can increase livestock productivity (Benites-Pariente et al., 2024).

Another crucial area for CRISPR-based microbiome engineering is pathogen control. Phage-delivered CRISPR systems have been created to reduce the prevalence of harmful bacterial strains without interfering with the beneficial microbiota by specifically targeting genes linked to virulence or antibiotic resistance. Antimicrobial resistance in livestock systems may be addressed by such interventions, especially if they are combined with stewardship guidelines and surveillance programs (Bogut et al., 2025).

Even with these developments, a number of technical issues still exist. A sophisticated knowledge of microbial networks and their host interactions is necessary to control unintended ecological effects, such as off-target gene edits or cascading changes in microbial consortia. These risks can be decreased by using conditionally active CRISPR systems, such as those that rely on specific cofactors or environmental cues, and synthetic biology tools that restrict activity to particular microbial taxa (Lopes & Prasad, 2024).

The implementation environment is also shaped by ethical and regulatory factors. Different international guidelines have an impact on how CRISPR-modified organisms are categorized, approved, and tracked, especially in intricate ecosystems like the rumen. Thorough risk assessments and open stakeholder engagement are required due to concerns about environmental containment, traceability, and long-term effects (Liberty et al., 2024).

Practically speaking, the stability and functionality of gene-editing components within livestock gastrointestinal systems depend heavily on developments in delivery platforms, such as anaerobe-compatible vectors and encapsulation techniques. At the same time, there are chances to completely reorganize the rumen microbiome through the creation of synthetic microbial consortia, which are groups of modified microorganisms designed for coordinated metabolic activity. Early introduction of these consortia can promote ideal rumen development, enhance immunological response, and lower emissions during critical physiological phases (Nazir et al., 2024).

Going forward, the next generation of sustainable livestock practices stands to benefit from CRISPR-based microbiome engineering. In addition to overcoming technical obstacles, its successful implementation will also depend on building social trust, adhering to ethical standards, and coordinating with more general health and climate objectives. It is anticipated to be crucial in changing how livestock systems strike a balance between resilience, productivity, and environmental responsibility as funding and interdisciplinary cooperation in this area increase.

### 4.3 Host-genome microbiome interactions

A key factor influencing livestock health, productivity, and environmental performance is the dynamic interaction between host genomic architecture and microbial community composition. Host genetic variation has a discernible impact on the composition and metabolic activity of the gastrointestinal microbiome through processes like nutrient provisioning, epithelial barrier function, and mucosal immune regulation. Emerging breeding and genomic selection techniques that optimize host-microbe symbioses for sustainable livestock production are based on these interactions (Guan, 2024; Rawal et al., 2024).

Numerous host loci that correlate with microbial community traits in different livestock species have been found through genome-wide association studies. Both commensal and pathogenic microorganisms' colonization dynamics have been demonstrated to be influenced by genes involved in immune recognition, such as those encoding pattern recognition receptors or major histocompatibility complex components. Similarly, changes in microbial diversity and abundance are linked to loci controlling mucin glycosylation, epithelial secretions, and antimicrobial peptides, which modulate the microbial niche. Beyond taxonomic shifts, these genetic influences also affect the microbial production of metabolites like short-chain fatty acids, which are critical for immune regulation and host energy metabolism (Khan et al., 2024a; Qadri et al., 2024).

Insights into the genomic regulation of microbial traits are being gained through integrative methods that integrate microbiome sequencing and host genotyping, commonly known as microbial quantitative trait loci (mbQTL) mapping. These analyses show that a significant amount of the variation in important microbial ratios, such as those related to methane production or fiber degradation efficiency, can be explained by host single-nucleotide polymorphisms (SNPs). Breeders can now target traits that simultaneously improve feed utilization and lower environmental emissions thanks to the introduction of microbiome-informed genomic estimated breeding values (EBVs) into livestock selection programs (Boggio et al., 2024; Martínez-Álvaro et al., 2024).

While the heritability of the composition of the microbial community is complex, it is now being studied across generations using advanced reproductive technologies such as embryo transfer. The study helps untangle the effects of vertical microbial transmission from direct genetic influences on the host. The interactions between host and microbes are further shaped by epigenetic mechanisms, including DNA methylation and histone modification, especially in the context of developmental planning and adaptation to environmental stress (Benga et al., 2024; Morris & Bohannan, 2024; Week et al., 2024).

Modifying host genes that regulate the composition and function of the microbiome is being investigated using emerging biotechnologies like CRISPR-based gene activation (CRISPRa). For example, increasing mucin-associated glycosyltransferases or antimicrobial lectins may encourage the colonization of beneficial bacteria or prevent enteric pathogens from adhering. By providing a new level of microbiome control, these host-directed tactics allow for a transition from passive microbial selection to active ecological engineering (Nazir et al., 2024; Zhong et al., 2025).

However, putting these realizations into action necessitates overcoming ethical, legal, and technical obstacles. It is still difficult to distinguish between direct genetic influences on microbial traits and indirect effects that are mediated by management, housing, or diet. Additionally, different regulatory frameworks classify genetic interventions that modify the microbiome differently; some treat them as standard breeding outcomes, while others demand distinct risk assessments for modified microbiomes (Fahmy, 2024; Ugwu et al., 2024).

Host-microbiome interactions are becoming more and more popular as strategic assets in livestock improvement initiatives from an economic standpoint. Producers can improve animal performance, lessen their dependency on antibiotics, and reduce greenhouse gas emissions by choosing genotypes that promote metabolically efficient and pathogen-resilient microbiota. Furthermore, ownership and access rights over patented microbial biomarkers and host-microbe association data are being addressed by evolving intellectual property frameworks (Guan, 2024; Okpara & Ozuo, 2024).

Modern research is now focusing on integrated strategies that administer synthetic microbial consortia and edit the host genome. In order to produce holobionts that are optimized for productivity, health, and environmental stewardship, these engineered ecosystems are made to work in concert with particular host genotypes. Precision breeding for symbiotic functionality is evolving from a theoretical concept to an operational reality as estimates of microbiome heritability across livestock species become more reliable (Fedorec, 2024; Nazir et al., 2024).

In conclusion, host-genome microbiome interactions offer a way forward for more robust, effective, and sustainable production systems, and they constitute a frontier in livestock genomics. Livestock are no longer bred as individual organisms but rather as integrated ecological units that co-evolve with their microbiota, thanks to this holistic viewpoint that has its roots in systems biology.

### 4.4 Precision phenotyping and genomic selection tools

Livestock breeding programs that aim to reduce GHG emissions are being revolutionized by the convergence of genomic selection tools and precision phenotyping. Through more focused and informed selection, this integrative approach not only improves the measurement of intricate, environmentally important traits but also speeds up genetic gains. The ability to collect comprehensive, high-throughput, and frequently real-time data on phenotypic traits, such as those about methane emissions, feed efficiency, and metabolic efficiency, is the foundation of precision phenotyping. Enteric methane emissions can now be directly measured in controlled and semi-commercial environments thanks to technologies like GreenFeed systems, portable accumulation chambers, and open-circuit respiration chambers. Additionally, new methods like sniffers and laser methane detectors provide non-invasive, affordable emissions monitoring, though their accuracy is still being improved (Dressler et al., 2024; Leishman et al., 2024; Ryan et al., 2024; Zhao et al., 2024).

These systems improve the temporal resolution and granularity of phenotypic data when combined with developments in automated data acquisition platforms, such as 3D imaging, infrared thermography, and machine vision. Crucially, advancements in omics-based proxy indicators (such as fecal volatile profiles or milk MIR spectra for dairy cattle) make it possible to estimate emission traits indirectly, which makes large-scale selection more feasible. To generate reliable phenotypic datasets for genetic analysis, it is crucial to ensure repeatability and standardization across various environments and production systems. This presents a challenge (Agiomavriti et al., 2024; Jiang & Zhu, 2024; Magro et al., 2024).

Genomic selection predicts an individual's breeding value for particular traits by utilizing the statistical correlation between phenotypes and genome-wide genetic markers. Methane yield (g CH₄/kg DMI), residual methane production, and feed conversion ratio are among the traits that are currently being incorporated into multi-trait selection indices for GHG mitigation. Even before emissions are measured, animals can be chosen based on their potential to emit less methane by using genomic estimated breeding values, or GEBVs. In species with lengthy generation intervals, like dairy cattle, where early selection greatly reduces breeding cycles, this is especially revolutionary (Domínguez-Castaño, 2024; Ryan et al., 2024; Worku, 2024).

The creation of sizable reference populations, which connect genotypic information to phenotypic records, improves genomic assessments. The foundation for integrating low-emission traits into standard genetic assessments has been established by national initiatives like New Zealand's Low Methane Sheep Breeding Program and EU-funded initiatives like RuminOmics. When creating high-accuracy genomic prediction models with techniques like GBLUP, BayesCπ, or deep learning frameworks, these reference populations are essential. Furthermore, functional genomic tools have been developed to improve the specificity of selection as a result of the identification of QTLs and important candidate genes related to rumen function, microbial interactions, and metabolic pathways (Domínguez-Castaño, 2024; Husien et al., 2024; Worku, 2024).

Integrating rumen microbiome profiles with host genome data to identify host-microbiome-genotype interactions is a particularly exciting area of research. These connections are essential to the production of methane because fermentation pathways are directly impacted by the composition and activity of the rumen microbial community. Rumen fermentation may be shifted away from methanogenesis through selective breeding for hosts that support a microbial profile with lower hydrogen availability or decreased populations of archaea. The use of microbiome heritability analyses and metagenome-wide association studies (MWAS) to comprehend and control these intricate relationships is growing (Marcos et al., 2024; Wang et al., 2024b).

Precision phenotyping also has connections to digital livestock farming, where breeding decisions can be dynamically adjusted thanks to continuous behavioral and physiological monitoring provided by sensor technologies and machine learning algorithms. Wearable technology, RFID-enabled systems, and automated feeding stations all provide real-time data streams that support trait monitoring of feed intake, activity patterns, and thermoregulation, all of which are indirectly related to emission efficiency. These data improve the accuracy of trait prediction, enable modeling of genotype-environment interactions, and aid in the production of animals that are environmentally resilient when incorporated into breeding programs (Arshad et al., 2024; Shoyombo et al., 2024; Ghavi Hossein-Zadeh, 2025).

Even with these technological developments, a number of problems still exist. The infrastructure needed for large-scale data storage and analysis, the expense of sophisticated phenotyping equipment, and the requirement for multi-stakeholder cooperation in data sharing and standardization continue to be major obstacles. Furthermore, the intricacy of striking a balance between productivity, health, welfare traits, and low-emission traits necessitates the creation of multi-objective breeding objectives backed by frameworks for economic weighting. To guarantee fair and responsible use, ethical issues surrounding the use of genomic data, especially in developing nations, must also be taken into account (Contaxis, 2024; Holden, 2024).

Looking ahead, the use of multi-omics integrative models that integrate genomic, transcriptomic, proteomic, metabolomic, and microbiomic data into unified selection tools is key to the future of precision breeding for emission reduction. With the aid of AI-powered analytics, this comprehensive framework will open the door to more individualized breeding tactics and a more nuanced understanding of the biological foundations of methane production. The development of dynamic, predictive, and self-improving breeding systems will become more practical as data science advances, helping to make the livestock sector more climate-resilient and sustainable (Zhao et al., 2024).

## 5 Manure management technologies

Previously considered an environmental liability, manure management technologies have become an essential part of sustainable livestock systems, transforming manure into a source of renewable energy, nutrient recovery, and soil regeneration. Effective manure management is crucial for reducing greenhouse gas emissions, improving resource efficiency, and promoting circular bioeconomy models as the volume and intensity of livestock production rise worldwide (Symeon et al., 2025).

Numerous technologies have been developed to capture and repurpose the nutrient loads and emissions related to the application and storage of manure. Anaerobic digestion systems, which use microbial consortia to transform organic matter into biogas, a renewable energy source mainly made up of carbon dioxide and methane, are among the most well-known. The captured gas can be used in on-farm combined heat and power systems or injected into natural gas grids thanks to developments in biogas upgrading technologies like membrane separation and pressure swing adsorption (Bumharter, 2024; Shah et al., 2024). Another advantage of these systems is the recovery of nutrients. By extracting important nutrients like nitrogen and phosphorus, methods like struvite crystallization and ammonia stripping lower the risk of eutrophication and make it possible to produce concentrated fertilizers. These techniques help to lessen dependency on synthetic fertilizers with large carbon footprints while simultaneously improving the agronomic value of products made from manure (Park et al., 2024; Sniatala et al., 2024).

Digital technologies are increasingly being incorporated into manure processing systems. The dynamic optimization of digester performance, feedstock composition, and hydraulic retention times is made possible by the integration of Internet of Things (IoT) sensors, machine learning algorithms, and real-time monitoring tools. Biogas yields and system reliability are improved by such precise management, especially in operations that deal with different manure characteristics (Premalatha et al., 2024; Swami et al., 2024).

Technologies for thermochemical conversion are also becoming more popular. Manure is transformed into biochar, hydrochar, or bio-oils by processes like hydrothermal carbonization and pyrolysis. These products can be used as soil amendments or renewable fuels. These procedures improve the safety and environmental acceptability of manure reuse by lowering pathogen load, odor emissions, and heavy metal mobility, among other advantages (Mei et al., 2024).

The adoption and expansion of manure management technologies are significantly influenced by policy and regulatory frameworks. Investment choices and the growth of integrated waste-to-resource infrastructures are being influenced by environmental regulations and incentive programs, such as carbon credit programs, feed-in tariffs for renewable energy, and nutrient trading platforms. Farms are being pushed to install advanced methane mitigation from manure storage facilities in some areas due to compliance requirements under emissions directives (Méité et al., 2024; Symeon et al., 2025).

Multifunctionality and integration are key components of emerging systems. Co-digestion techniques that mix manure with food processing byproducts or agricultural residues improve energy recovery and microbial activity while lowering input variability. At the same time, odor control innovations like biofiltration and sophisticated plasma-assisted scrubbers are resolving community concerns and bolstering intensive livestock operations' social license (Adnane et al., 2024; Kadam et al., 2024).

Livestock operations are being redefined as nodes of bioresource generation from a systems perspective by next-generation manure management technologies. Farms can become hubs that provide carbon-negative fertilizers, biologically active soil amendments, and renewable energy by integrating these technologies into larger circular economy frameworks. This multifaceted role is increasingly seen as a strategic asset in the creation of sustainable protein supply chains, rather than merely a compliance requirement (Bumharter, 2024; Pata & Patel, 2024).

The technologies for managing manure offer significant potential in reducing greenhouse gas emissions, but their usefulness is very context-dependent. Depending on the climatic conditions, manure properties, and the size of farms, the effectiveness of mitigation strategies such as anaerobic digestion, composting, and solid-liquid separation varies greatly. Temperature and moisture conditions have a strong impact on microbial activity and gas generation, and variability in manure structure between species and production systems influences the results of treatment. Thus, a technology that works best in temperate systems at scale can have significantly different outcomes in a tropical or large system, and it is essential to consider that the reports of mitigation efficiencies have distinct implications for a particular environment and operation of a system (Yan et al., 2024; Badzmierowski et al., 2025; Symeon et al., 2025).

In addition to technical performance, the economic viability and functionality of manure management technologies are very important factors of adoption. Higher systems may entail a lot of capital input, technical skills, and continuous maintenance, making them unavailable to smallholder or resource-constrained farms. Although the emission reduction potential is high, there might be obstacles to long-term implementation based on labor requirements, system stability, competency, and the skills and expertise to operate the system. These limitations emphasize the need to scale manure management solutions to the size of the farm and available resources and come up with modular and affordable designs to increase adoption (Orner et al., 2021; Macena et al., 2025).

The technologies of treating manure can create significant trade-offs in terms of greenhouse gas reduction and efficiency of nutrient recycling. Although some of the treatments are successful in curbing the emissions of methane, they can create more nitrogen loss through volatilization or result in a different chemical form and bioavailability of nutrients like phosphorus. The alterations in the nutrient retention may impact the downstream nutrient cycling, the potential of substituting fertilizers, and the sustainability of the entire system. To assess manure management options, it is thus necessary to evaluate these two aspects of a combined evaluation, which weighs the benefits of reduction in emissions against the effects on nutrient recovery and circularity (Badzmierowski et al., 2025; Wodrich et al., 2026).

Manure management has a wider impact than emission control, not only on the soil fertility and crop productivity but also on the products of animals, which have a secondary effect. The difference between treated manure and raw manure can be quite significant, depending on the nutrient composition, the organic matter content, and the microbial activity, which also results in soil structure, nutrient availability, and long-term soil health. These alterations can also affect forage quality and crop yields, and there could be feedback on the systems producing milk and meat. It is thus essential to include downstream agronomic and productivity effects in the manure management assessment to have a comprehensive review of the sustainability outputs (Sant’Anna et al., 2024; Symeon et al., 2025).

Maintenance requirements, energy inputs, and possible environmental side effects also determine whether the manure management technologies will be operated sustainably. The systems can cause secondary environmental hazards, such as the formation of leachates, the release of odors, or unwanted runoff of nutrients if they are not handled properly. Attributes of energy needs within the operation, transport, or processing may also be additional factors in the net environmental gains, especially when there is minimal integration of renewable energy. All these aspects should be put into consideration to determine the overall sustainability and resilience of manure treatment systems (Jabłoński et al., 2024; Kočetkovs & Zvirbule, 2025).

Relative analysis of traditional and modern technologies of manure management is capable of locating the best practices and solutions relevant to the situation. Advanced technologies might have a better mitigation potential, but in some environments, simpler methods might offer more strength and be less expensive. New trends in automation, real-time analytics, and automated farm management systems present new opportunities to enhance the level of efficiency, extract nutrients more efficiently, and ease the load of the work. This adds to the stability of the processes and helps them to adapt to any production setting by integrating sensors and data-driven control systems (Yan et al., 2024; Symeon et al., 2025).

The focus of managing manure needs to be presented as an ancillary part of combined mitigation efforts as opposed to a solitary measure. It works best when combined with dietary interventions, genetic advancement, and accurate livestock administration practices. Local climate, infrastructure, and socio-economic factors should be considered to adapt manure technologies in the region to guarantee the wide applicability and long-term consequences. By integrating the manure management control inside the systematic farm-level approaches, the manure management contributes to enhancing the process of creating the climate-resilient livestock management with resource-saving functions (Singh et al., 2025; Symeon et al., 2025).

**Fig. 4:**
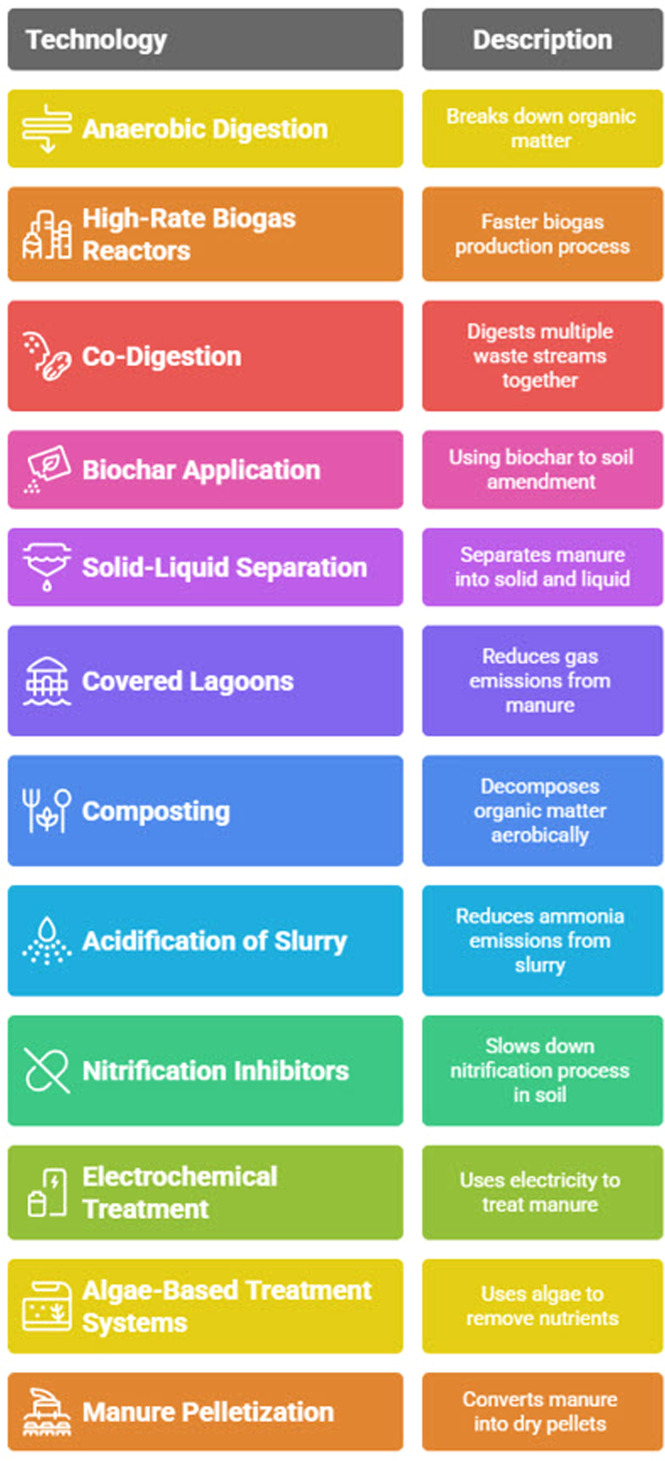
Advanced manure management technologies for GHG mitigation in livestock.

Manure management is now a proactive sustainability strategy rather than a reactive environmental control measure. It now offers a triple value proposition, lowering emissions, improving nutrient cycles, and boosting on-farm resilience, thanks to technological advancement and system integration. The development and uptake of manure management technologies will be crucial to creating livestock production systems that are more socially acceptable, economically feasible, and climate resilient as resource and climate pressures increase. Advanced manure management technologies for GHG mitigation in livestock are shown in Table 4 and Fig. 4.

**Table 4:** Advanced manure management technologies for GHG mitigation in livestock.

Technology	Mechanism	GHG reduction (CO₂eq/ton)	Advantages	Disadvantages/Challenges	Recent references
Anaerobic digestion	Converts organic matter into biogas through microbial processes	500–1200	Produces renewable energy, reduces methane emissions	High initial cost, requires skilled operation	Mishra et al. (2025) Subramanian and Suresh (2024)
High-rate biogas reactors	Accelerated biogas production via enhanced microbial efficiency	1000–1500	Compact, efficient, increased biogas yield	Sensitive to feedstock quality and hydraulic load	Dalkılıç& Uğurlu (2024) Kaur et al. (2024)
Co-digestion	Combines manure with other organic wastes to optimize biogas yield	800–1400	Maximizes resource use, higher energy recovery	Logistic complexity, feedstock availability	Ikpe et al. (2024) Oghoghorie et al. (2024)
Biochar application	Stabilizes carbon in soil, reduces volatilization, and nitrous oxide	200–600	Enhances soil fertility, long-term carbon storage	Variable efficacy, sourcing, and transport issues	Luo et al. (2024) Tiong et al. (2024)
Solid-liquid separation	Reduces methane by separating volatile solids from liquid manure	100–300	Reduces storage emissions, improves nutrient management	Disposal of solids, limited effect alone	Liao et al. (2025) Støckler et al. (2025)
Covered lagoons	Methane capture via impermeable covers on manure lagoons	400–1000	Simple design, reduces odor and methane	Requires maintenance, climate-sensitive	Chiodini et al. (2023) Rahic et al. (2025)
Composting	Aerobic treatment reduces methane and nitrous oxide	150–500	Produces usable compost, low-tech	Still emits some GHGs, odor management	Valverde-Orozco et al. (2024) Du et al. (2024b)
Acidification of slurry	Reduces ammonia and nitrous oxide emissions via pH control	200–600	Enhances nutrient retention, reduces odor	Corrosive materials, potential soil acidification	Finzi et al. (2024) Langley-Randall et al. (2024)
Nitrification inhibitors	Chemical inhibitors reduce N₂O emissions from manure	100–400	Effective with minimal equipment	Potential environmental risks, variable efficacy	Wodrich et al. (2026) Toda et al. (2026)
Electrochemical treatment	Converts ammonia to N gas, reducing emissions and recovering nutrients	500–900	High nutrient recovery, minimal emissions	Expensive, still under development	Wang et al. (2024c) Huang et al. (2024b)
Algae-based treatment systems	Algae cultivation absorbs nutrients and stabilizes emissions	150–400	Co-products possible (biofuel, feed), environmentally friendly	Limited scalability, light, and nutrient dependency	Yang and Lu (2025) Munir et al. (2025)
Manure pelletization	Drying and compressing manure reduces microbial decomposition	300–700	Easy transport and storage, marketable product	Energy-intensive, the cost of processing	Singh et al. (2024b) Alege et al. (2023)

### 5.1 Anaerobic digestion advances

#### 5.1.1 High-rate biogas reactors

By using sophisticated hydrodynamic designs and engineered microbial ecologies, high-rate biogas reactors offer a notable increase in volumetric methane productivity, marking a revolutionary development in anaerobic digestion technologies. These systems, in contrast to traditional continuously stirred tank reactors, are made to maximize the physical and biological aspects of digestion, leading to improved stability, throughput, and energy yield (Dalkılıç & Uğurlu, 2024; Kaur et al., 2024).

The formation of dense microbial consortia, which are frequently organized in granular sludge or biofilm matrices and facilitate syntrophic interactions between fermentative bacteria and hydrogenotrophic methanogens, is the fundamental component of these systems. By facilitating direct interspecies electron transfer (DIET), these consortia increase metabolic efficiency by avoiding the restrictions of hydrogen diffusion. High-surface-area polymer matrices and other materials act as scaffolds for microbial attachment, improving biomass density and retention while encouraging effective gas-liquid-solid separation (Dai, 2024; Tang et al., 2024).

These systems also use computational optimization to fine-tune fluid dynamics in order to improve flow distribution and minimize dead zones. As a result, the reactor environment becomes more uniform, supporting uniform microbial activity and lowering the risk of localized acidification. To meet the various physiological needs of various microbial groups, hydrolysis and acidogenesis can be thermally or spatially isolated from acetogenesis and methanogenesis in multi-phase configurations. This enhances the breakdown of lignocellulosic materials, especially in substrates high in fiber, like swine or cattle manure (Jujjavarapu et al., 2024; Wongsirichot, 2024).

Tools for real-time monitoring are being used more and more to preserve system performance. Continuous data on important process indicators like volatile fatty acids, ammonia concentrations, and chemical oxygen demand are provided by spectroscopic and sensor-based platforms. Machine learning algorithms use this data to dynamically modify organic loading rates and retention periods, guaranteeing process stability even in the face of changing feedstock conditions (Mittu et al., 2024; Zhu, 2024).

Side-stream technologies are commonly used to incorporate nutrient recovery into high-rate digestion systems. Nitrogen can be captured and converted into fertilizers with added value using methods like gas-permeable membranes, struvite precipitation, and ammonia stripping. This closes nutrient loops within the farming system and lowers the risk of methanogen inhibition due to elevated total ammoniacal nitrogen levels (Nagarajan et al., 2024; Proskynitopoulou et al., 2024).

The methane content of the biogas is further enhanced by novel configurations that also include bioelectrochemical systems, in which leftover organics are oxidized at bioanodes to produce hydrogen. These hybrid methods make digestate more suitable for agricultural reuse by enhancing energy recovery, reducing pathogens, and sanitizing effluent (Callegari et al., 2025).

Reactor design structural advancements, like modular construction with cutting-edge materials, have improved scalability and decreased capital costs. Automated foam control systems and intelligent feedstock blending algorithms are examples of operational improvements that have decreased downtime and improved co-digestion performance across a variety of manure types and carbon sources (Huang, 2024).

Notwithstanding these advancements, there are still difficulties, especially when it comes to growing and maintaining granular sludge in a variety of agricultural settings. The goal of ongoing advancements in microbial seeding techniques and inoculum pretreatment is to increase reactor start-up speed and process perturbation resilience (Cao et al., 2024a).

The implementation of high-rate digesters is still influenced by sustainability guidelines and policy frameworks, particularly in areas with goals for reducing emissions and producing renewable energy. According to life cycle assessments, these systems have a number of advantages, such as lowering greenhouse gas emissions, increasing farm energy independence, and improving soil quality by using stabilized, carbon-rich digestate (Greene et al., 2024; Zhang et al., 2024).

To sum up, high-rate biogas reactors are a prime example of how engineering, microbiology, and data science are combined in contemporary manure management. They serve as a foundation for livestock operations that are both economically feasible and climate resilient by optimizing energy conversion efficiency and permitting nutrient recovery in a small, modular format.

#### 5.1.2 Co-digestion with agricultural waste

Co-digestion with agricultural waste has become a key tactic to improve anaerobic digestion systems' performance and environmental efficiency. Co-digestion reduces the problems caused by the unbalanced carbon-to-nitrogen (C/N) ratios of manure mono-digestion by mixing livestock manure with complementary organic substrates. By promoting synergistic microbial interactions, these feedstock combinations improve the stability and metabolic efficiency of the digestion process and greatly boost the yield of biogas (Ikpe et al., 2024; Oghoghorie et al., 2024).

The key to co-digestion's success is its capacity to maintain a variety of microbial consortia by balancing biochemical composition and nutrient availability. Cellulolytic bacteria and methanogenic archaea are encouraged to cross-feed when lignocellulosic agricultural residues are mixed with nutrient-rich manures. This speeds up the breakdown of complex fibers and increases methane production without going over the inhibitory thresholds for volatile fatty acids or ammonia. Advanced pretreatment techniques, like steam explosion followed by enzymatic hydrolysis, increase fibrous co-substrates' biodegradability and release previously resistant carbon fractions (Erkuş & Tuncay, 2024; Ko et al., 2024).

To optimize co-digestion systems, technological integration is essential. Incoming feedstocks are now being characterized in real time using biochemical monitoring instruments and hyperspectral imaging sensors. These systems make it possible to dynamically modify substrate ratios in order to avoid process imbalances and preserve steady operating conditions. Methanogenic activity is consistently monitored, and feeding strategies are guided by indicators like the ratio of volatile fatty acids to alkalinity (FOS/TAC) (Rusanowska et al., 2025).

Co-digestion improves waste valorization and nutrient recycling in addition to improving energy recovery. The procedure supports closed-loop nutrient management by making it easier to recover nitrogen and phosphorus in stabilized forms that can be used again in agriculture. In areas where water is scarce, technologies like gas-permeable membrane contactors are used to recover ammonia as liquid fertilizers, lowering digestate salinity and improving the effluent's suitability for land application (Rao et al., 2024; Rusanowska et al., 2025).

By using agro-industrial by-products like fruit pomace, winery residues, or sugar beet pulp, co-digestion also offers chances for integrated waste management. When appropriately handled, these substrates not only increase the digester's energy capacity but also divert large amounts of organic waste away from open burning and landfills. To prevent microbial inhibition, specific pretreatment procedures, such as delinting or activated carbon filtration, are necessary when certain agricultural residues contain inhibitory compounds, such as gossypol in cotton waste or limonene in citrus peel (Bhattacharya et al., 2024).

Several jurisdictions have simplified the classification and approval process for co-substrates, which promotes the growth of farm-scale digesters that incorporate a variety of biomass sources from a regulatory standpoint. Incentive structures in waste management and renewable energy frameworks, such as eligibility for energy credits and feedstock subsidies linked to waste diversion, further encourage co-digestion (Ahuja et al., 2024; Kadam et al., 2024). Additionally, bioaugmentation, the introduction of particular microbial consortia to improve resilience and substrate conversion, is being used to improve the microbial dynamics of co-digestion, especially in systems processing lipid-rich or proteinaceous waste. This keeps inhibitory intermediates from building up and maintains reactor performance when organic loading is high (An et al., 2024).

In the future, co-digestion procedures should be further improved by combining robotic automation and artificial intelligence. To improve substrate blending and expedite feedstock preparation, smart sorters with real-time waste classification and contamination removal capabilities are being developed. These developments are in line with livestock agriculture's larger shift to digitally managed, circular bioenergy systems (Rastogi et al., 2025).

To sum up, co-digestion with agricultural waste is a multi-benefit, scalable way to improve anaerobic digestion. It makes a significant contribution to the decarbonization and sustainability of livestock production systems by fusing environmental preservation with energy generation and nutrient recycling.

#### 5.1.3 Biochar integration in digesters

An inventive fusion of materials science and microbial ecology is the use of biochar in anaerobic digestion systems. Biochar is increasingly being designed to function as a microbial habitat and a biochemical catalyst through surface functionalization techniques like doping and sulfonation, which greatly improve digestion kinetics, methane yield, and system resilience (Ma et al., 2024a; Ngo et al., 2024).

The ability of biochar to promote DIET in digesters is one of its main benefits, especially in situations with suboptimal thermal conditions where traditional routes are less efficient. Biochar improves syntrophic cooperation between methanogenic archaea and fermentative bacteria by acting as a redox-active scaffold. Even in unheated digesters running in mesophilic to psychrophilic conditions, sustained methanogenesis is supported by engineered biochars with specific surface characteristics, such as enhanced electron shuttle capacities and functional group density (Luo et al., 2024; Tiong et al., 2024).

Biochar exhibits a high adsorption affinity for a variety of chemical contaminants in addition to its electrochemical advantages. Functionalized biochar variations have been demonstrated to sequester antibiotic residues and other emerging pollutants in manure-based digesters, lowering microbial inhibition and protecting core methanogenic populations in the process. By enabling the catalytic conversion of hydrogen sulfide into elemental sulfur, certain modifications, like the addition of metal oxides, have removed the need for post-digestion biogas scrubbing and aided in resource recovery efforts (Ganguly & Chakraborty, 2024; Jeyaraj et al., 2024).

From the standpoint of the circular economy, digestate enhanced with biochar can be used for purposes other than soil amendment. Biochar enhances microbial colonization, nutrient accessibility, and moisture regulation when utilized as a substrate for insect bioconversion platforms, such as the cultivation of black soldier fly larvae. This speeds up the production of biomass. By turning organic waste into high-protein feed and biofertilizers and sequestering carbon, these systems exemplify the concepts of waste valorization and nutrient loop closure (Ganesan et al., 2024; Sánchez‐Hernández & Megharaj, 2024).

Additionally, *in situ* activation of biochar within digesters has been made possible by emerging technologies. Biochar's internal carbon matrices are restructured by microwave-assisted pyrolysis and other thermal processes, increasing its porosity and surface reactivity. When combined with anaerobic digestion systems, this increases microbial colonization and speeds up methanogenesis. Because of its potential for long-term carbon sequestration, biochar has been formally included in carbon certification frameworks, provided that it exhibits low environmental leakage and carbon stability (Ngo et al., 2024; Zbair et al., 2024).

Biochar acts as a habitat modifier at the microbial level, affecting the metabolic activity and abundance of important functional guilds. Propionate-oxidizing and hydrogen-scavenging taxa are more abundant in digesters treated with biochar, according to metagenomic research, which increases process stability and speeds up intermediate turnover. Biochar acts as a charge buffer when combined with electrochemical regulation, temporarily storing and releasing redox equivalents in response to changes in organic loading. This evens out methane production variations and synchronizes biogas generation with changing energy grid demands (Ding et al., 2024; Pei et al., 2024).

Although biochar has potential, there are obstacles to its widespread use in anaerobic digesters. To avoid unintentional microbial inhibition, material consistency needs to be carefully controlled, especially concerning ash content and trace element composition. Furthermore, improvements like binder integration and nanostructural reinforcement are required to maintain performance over time due to the mechanical durability of biochar under constant agitation (Viaene, 2024; Zbair et al., 2024).

The holistic benefits of incorporating biochar into digestion systems are being highlighted more and more by life cycle analyses. These evaluations highlight the potential for carbon removal, improved nutrient recycling, and energy self-sufficiency, establishing biochar as a crucial component of regenerative livestock systems. Biochar-enabled digesters are pushing the larger goal of climate-smart agriculture by redefining the line between resource generation and waste management as technological and regulatory frameworks change (Adhikari et al., 2024; Silva et al., 2025).

### 5.2 Nutrient recovery systems

#### 5.2.1 Nitrogen capture technologies

By transforming reactive nitrogen species into products with added value, modern nitrogen capture technologies are revolutionizing manure management, reducing atmospheric emissions, and promoting circular nutrient use. Before nitrogen, especially in the form of ammonia, volatilizes into the environment or pollutes water, these technologies are intended to capture and stabilize it. In addition to lowering odor emissions and greenhouse gas precursors, this method makes it easier to recover concentrated nitrogen fertilizers that can be used again in agriculture (Patel, 2024; Tahmid et al., 2024).

Systems based on membranes have become very effective ammonia recovery tools. In order to create stable ammonium salts, these employ semi-permeable materials to allow gaseous ammonia from manure effluents to selectively diffuse into acid-based capture solutions. The effluent's pH and the membrane material's characteristics are precisely adjusted to maximize mass transfer effectiveness and reduce nitrogen loss. In parallel, nitrogen can be recovered as magnesium ammonium phosphate using struvite precipitation reactors that have been optimized. These systems are especially useful in places with stringent water quality regulations because they create granulated slow-release fertilizers that aid in phosphorus recovery through chemical dosing and controlled crystallization (Farghali et al., 2024; Wu et al., 2024b).

Ammonium ions are also being separated and recovered from liquid waste streams using electrochemical and bioelectrochemical techniques. By using electrical potential to promote selective ion transport across membranes, these technologies capitalize on the charge characteristics of ammonia. Microbial fuel cells are incorporated into some sophisticated systems, allowing for the simultaneous production of energy and nutrient recovery. A promising first step toward energy-neutral or even energy-positive nutrient recovery operations is this dual functionality (Mohamed et al., 2025).

Microwave-assisted stripping has drawn interest in thermal processes because it uses dielectric heating, which excites water molecules selectively, to volatilize ammonia. When compared to traditional thermal stripping, this method uses less energy and works well for decentralized applications. Similar advancements have been made in adsorption-based technologies, with new materials like metal-organic frameworks and functionalized biochars providing high selectivity and ammonium binding capacity. In accordance with the ideas of sustainable waste management, these sorbents can be recycled and used again (Ogunniyan, 2024).

Under varying manure compositions, hybrid systems that integrate chemical, physical, and biological processes provide increased flexibility and resilience. Modular units with membrane contactors and sophisticated oxidation techniques, for example, can treat leftover organics and capture nitrogen, enabling the reuse of effluent in non-potable applications like equipment cleaning. The efficiency of nitrogen transformation during the separation of liquid and solid manure fractions is further enhanced by advances in biological augmentation, especially the application of engineered nitrifying bacteria (Askari et al., 2024; Ejairu et al., 2024).

Large-scale initiatives are starting to show that incorporating nitrogen capture into more comprehensive waste-to-resource frameworks is feasible. Recovered ammonia can be transformed into high-purity chemical feedstocks or synthetic fuels using technologies like cryogenic condensation, vacuum stripping, and catalytic reforming, thereby connecting nutrient management with decarbonization tactics. The capacity of catalytic reactors and plasma-assisted processes to convert ammonia into hydrogen-rich syngas is also being tested as a means of producing renewable energy (La Corte et al., 2025).

Overall, the development of nitrogen capture technologies represents a paradigm shift in the management of livestock waste, one that embraces energy integration, nutrient circularity, and agricultural sustainability in addition to pollution control. The use of these systems is being accelerated by life cycle assessments, regulatory incentives, and precision agriculture tools, highlighting their significance in climate-smart livestock operations. Nitrogen recovery is positioned to become a key component of regenerative livestock systems as research into inhibitory substances in manure matrices and system efficiency continues.

#### 5.2.2 Phosphorus recycling methods

In light of the worldwide loss of high-grade phosphate rock and growing environmental regulations, recycling phosphorus from livestock manure has become an essential part of sustainable nutrient management. These days, sophisticated recovery techniques use a range of physicochemical and biological processes to extract phosphorus from waste streams and transform it into forms that are beneficial to agriculture while reducing the risk of eutrophication from runoff and leaching (Behjat et al., 2024; Nadagouda et al., 2024).

Systems based on crystallization are essential to contemporary phosphorus recovery. These technologies use continuous-flow reactors to precipitate phosphorus as struvite or hydroxyapatite, which depend on pH regulation and supersaturation conditions. Specifically, the production of slow-release fertilizer granules and effective phosphorus capture are made possible by the use of magnesium salts or waste-derived calcium sources. While ion exchange and electrochemical separation systems provide alternate routes by concentrating phosphate ions onto specific adsorbent surfaces, such as layered double hydroxides functionalized with organic ligands or rare-earth coated electrodes, reactor design innovations have improved process stability (Yao et al., 2024a; Chen et al., 2025a).

New techniques also include pyrolytic and hydrothermal treatments with microwave assistance, which convert manure into hydrochars and phosphorus-enriched biochars at the same time. These thermochemical methods align nutrient recovery with public health standards by degrading leftover medications and pathogens in addition to concentrating phosphorus into stable, plant-available forms. Engineered microbial strains are used in certain systems to hyperaccumulate intracellular polyphosphate, which enables biological phosphorus capture through the harvesting and processing of biomass later on. High specificity and compatibility with aerobic manure treatment processes are advantages of this biologically mediated method (Ahmed et al., 2024b; Elser et al., 2024).

High-purity phosphoric acid is another method of recovering phosphorus through hybrid extraction technologies that blend solvent-based separation with acid solubilization. These are especially important in regulatory settings where high rates of recovery from organic waste streams are required. Membrane-based systems that use nanofiltration or ligand-exchange technologies have also demonstrated promise in the selective extraction of phosphate ions from intricate liquid matrices, resulting in concentrated ammonium phosphate solutions that work well with fertigation systems (Huang et al., 2024a; Yao et al., 2024b).

Pre-treatment procedures employing alkaline hydrolysis or the use of selective sorbents, such as magnetic resins and functionalized nanomaterials that provide high phosphate selectivity and reusability, are being used to address operational challenges, such as silica interference from rice-husk-amended feedstocks. The scalability of these systems in both temperate and industrial regions is further supported by recent advancements in cryogenic concentration techniques, which use low-temperature separation to separate manure into phosphorus-rich fractions with lower energy requirements (Parrillo & Sańchez, 2024).

Integrated technologies that generate energy and recover phosphorus are also becoming more popular. For instance, microbial fuel cells enable phosphate electromigration and organic matter degradation to occur simultaneously, enabling the co-production of struvite-based fertilizers and renewable electricity. Advanced bioelectrochemical configurations maximize energy yields and recovery, demonstrating how resource-efficient wastewater treatment and circular economy principles can coexist (Abdalla et al., 2024; Pérez-Bernal et al., 2024).

Policy frameworks that limit phosphorus discharge into delicate aquatic ecosystems and provide incentives for nutrient recovery are increasingly supporting these innovations. The increasing use of phosphorus recovery systems on commercial livestock farms is indicative of their role in climate-smart agriculture, national fertilizer independence, and nutrient stewardship. These technologies are changing the value proposition of manure from waste to resource, as life-cycle assessments continuously show the economic and environmental benefits of recovered phosphorus over synthetic alternatives. By doing this, they are supporting agri-food systems that are more robust, circular, and regenerative (Behjat et al., 2024; González et al., 2024).

#### 5.2.3 Electrochemical ammonia recovery

With the ability to selectively separate and transform ammonium ions from livestock manure into useful nitrogen-based fertilizers, electrochemical ammonia recovery is becoming a game-changing technique in nutrient recycling systems. In order to help ammonium migrate across compartments and into acidic recovery phases, where it is stabilized and captured as ammonium salts or gaseous ammonia, these technologies make use of electrochemical gradients, ion-selective membranes, and sophisticated catalytic surfaces. This precision-driven mechanism offers a more energy-efficient substitute for traditional stripping and scrubbing procedures, supporting the objectives of on-farm nutrient circularity and environmental compliance (Patel, 2024; Yang & Qin, 2024).

Innovations in cell design and electrode materials are essential to these systems. Even in the presence of competing cations, ammonium ions can be selectively transported and adsorbed with high affinity using coated electrodes made of sophisticated conductive nanomaterials like metal hydroxides or layered carbides. These materials enable sustained ion separation during continuous operation due to their high surface charge density and enhanced selectivity. Simultaneously, integrated systems that combine microbial electrolysis cells and gas-diffusion cathodes oxidize organic matter in manure using bioelectrochemical processes, providing electrons for ammonium reduction and recovery. In addition to improving energy recovery, this coupling stabilizes nitrogen in forms that are suitable for the manufacturing of commercial fertilizer (Burns et al., 2024; Yang et al., 2025).

Recent advancements in pulsed electrochemical operation and lattice-mediated charge transfer have improved the conversion of ammonium to ammonia under mild pH conditions, reducing the need for chemical pretreatment and expanding operational applicability across diverse manure matrices. Ammonium is concentrated in liquid streams by hybrid systems that combine gas-permeable membranes and capacitive deionization before it is recovered by chemical absorption or vacuum-assisted membrane stripping. High-purity final products, such as concentrated ammonium nitrate solutions and anhydrous ammonia, are produced by these configurations and can be used directly in fertigation and other agricultural systems (Moeller et al., 2024; Truong et al., 2024).

The compatibility of electrochemical ammonia recovery with carbon mitigation programs improves its economic and environmental viability. These systems reduce emissions and qualify for carbon credits under various regulatory frameworks by replacing synthetic nitrogen fertilizers made through energy-intensive processes. Additionally, new methods that use biodegradable cell materials and light-driven photoelectrochemical cells are raising the sustainability profile of these systems by integrating renewable energy sources and minimizing their environmental impact (Safour, 2024; La Corte et al., 2025).

In order to prolong operational lifespan and sustain performance in the corrosive environments typical of manure treatment, surface modifications and protective coatings are being used to address issues like electrode fouling and system durability. Using real-time conductivity and flow rate data as input, adaptive control algorithms dynamically adjust system parameters to maximize recovery while preventing the production of unwanted byproducts like chlorinated compounds. In order to guarantee consistent performance under fluctuating on-farm conditions, advanced modeling and sensor integration are being used more and more (Bailey, 2024; Cairone et al., 2024).

Looking ahead, the integration of electrochemical ammonia recovery with energy systems, such as plasma-catalytic reformers capable of converting recovered ammonia into hydrogen-rich syngas, positions livestock operations as self-sustaining hubs for fertilizer and energy co-production. Novel materials such as molecularly imprinted polymers with high selectivity for ammonium ions, as well as magnetically responsive composites that enable rapid regeneration, are further pushing the boundaries of efficiency and scalability. These developments support the vision of decentralized, circular nitrogen management frameworks in agriculture, with pilot systems already demonstrating regional supply contributions in fertilizer markets. In this context, electrochemical ammonia recovery not only mitigates environmental impacts but also redefines the role of livestock manure as a feedstock for high-value resource generation (Moeller et al., 2024; Patel, 2024; Tort et al., 2024).

### 5.3 Novel and thermochemical manure valorization technologies

Recently, there has been a lot of interest in thermochemical and novel manure valorization technologies as revolutionary tools for enhancing sustainable waste management in livestock production systems. These technologies offer comprehensive solutions to several pressing issues, including the production of renewable energy, the recovery of vital nutrients, and the reduction of greenhouse gas emissions (Boakye-Yiadom et al., 2024). Traditional methods of managing manure, such as land application and lagoon storage, often lead to significant emissions of the potent greenhouse gases CH₄ and N₂O, in addition to issues with nutrient leaching and odor. On the other hand, thermochemical processes, which operate under controlled high-temperature conditions, can convert animal manure into end products that are both environmentally beneficial and energy-dense. These technologies offer a paradigm shift in the way that livestock waste is viewed, not as a burden but as a valuable feedstock for models of the circular bioeconomy, by significantly changing the chemical composition of manure (Pata et al., 2024; Riau et al., 2024; Symeon et al., 2025).

Pyrolysis, a process that thermally breaks down organic material without oxygen at temperatures usually between 300 and 700°C, is one of the most researched thermochemical techniques. Three primary products are produced by pyrolyzing livestock manure: syngas (gas), biochar (solid), and bio-oil (liquid) (Altikat et al., 2024; Tarafdar et al., 2025). Because of its many uses, biochar has attracted special attention. When added to soil, it can improve microbial activity, soil fertility, and water retention while also serving as a long-term carbon sink by stabilizing organic carbon for centuries. Biochar is a crucial instrument for mitigating climate change and promoting sustainable agriculture since it has also been demonstrated to lower soil N_2_O emissions and increase nutrient-use efficiency. On the other hand, the bio-oil and syngas fractions can be upgraded into higher-value chemicals or utilized as renewable fuels for heating and electricity production, allowing farms to become self-sufficient in energy. More control over product distribution and yield is now possible thanks to reactor design advancements like temperature-controlled units and continuous feed systems, which are crucial for commercialization (Keerthi, 2024; Majumder et al., 2024; Sharma & Chhabra, 2024).

Another thermochemical process (gasification) involves the introduction of a regulated amount of oxygen or steam to partially oxidize manure at temperatures usually in the range of 800–1000°C. The result is syngas, a combustible mixture consisting primarily of H₂, CO_2_, and CH₄, which can be used directly for electricity generation or, by Fischer-Tropsch synthesis, can be refined into a liquid. The gasification process is particularly suitable for treating manure with a low moisture content or when combined with co-substances such as crop residues, dust, or poultry litter to optimize the nutrient content. Considering the density and versatility of syngas, it is a promising intermediate for renewable energy (Chanthakett et al., 2024; Narnaware & Panwar, 2024). In addition, recent innovations in plasma gasification and catalytic tar reforming address common operational issues, such as the production of tar and ash, thus improving the reliability and durability of the system. Gasification has a strong potential for integration into large-scale, centralized treatment centers for livestock waste, given its higher throughput than bio-based treatments and the ability to process mixed feedstocks (Jayanarasimhan et al., 2024; Prasetiyo et al., 2024).

Hydrothermal carbonization (HTC) and hydrothermal liquefaction (HTL), two cutting-edge hydrothermal technologies, present attractive options for handling high-moisture manure, a waste stream that presents difficulties for drying-dependent procedures like gasification and pyrolysis. In the presence of water, HTC works at moderate temperatures (180–250°C) and high pressures, turning manure into hydrochar, a solid product rich in carbon, and an aqueous phase that is nutrient-rich. Hydrochar can be pelletized and used in power plants in conjunction with coal, or it can be used in agricultural soils like that of biochar. In contrast, HTL uses higher temperatures (250–350°C) and pressures (up to 20 MPa) to break down organic compounds into biocrude oil, simulating the geological formation of fossil fuels. It is possible to transform this biocrude into drop-in fuels that work with the current petroleum infrastructure. Crucially, both HTC and HTL naturally lower the levels of pathogens, medications, and odorants found in raw manure, improving the safety of the environment and the acceptability of manure reuse in society. These technologies enable waste treatment without requiring energy-intensive dewatering procedures, which is a major advancement for intensive livestock operations, particularly in areas with water scarcity (Devnath et al., 2024; Singh et al., 2024a; Vadlamudi et al., 2024).

Researchers are investigating integrated systems that combine thermal treatment with nutrient recovery, emission control, and by-product utilization in an effort to further improve the economic and environmental feasibility of thermochemical valorization. To close nutrient loops and stop eutrophication, syngas can be post-treated with struvite precipitation units or ammonia scrubbers to recover nitrogen and phosphorus for use as fertilizers. Manure-derived functionalized biochar that has been impregnated with metal oxides or minerals can be used as a pH buffer, slow-release fertilizer, or adsorbent for pollutants in soil and water systems. Furthermore, some pilot projects are investigating the use of chars made from manure as carriers for advantageous microorganisms in bioreactors or as filtration media in artificial wetlands. The role of thermochemical technologies in regenerative and circular agricultural frameworks is further reinforced by these multipurpose applications. Furthermore, real-time monitoring and adaptive optimization of operational parameters are made possible by the incorporation of digital tools like artificial intelligence and machine learning algorithms into process control systems (e.g., temperature, residence time, and feedstock input), enhancing reliability, cutting down on downtime, and lowering energy losses (Jabłoński et al., 2024; Pelaez-Samaniego et al., 2024; Zin et al., 2024).

The broad use of thermochemical manure valorization technologies is currently limited by a number of technological, financial, and legal obstacles, despite their revolutionary potential. High operating costs and capital expenditures continue to be major obstacles, especially for small-scale or resource-constrained farmers. Process standardization and end-product quality control are made more challenging by the variation in manure composition among species, diets, bedding materials, and regional practices. The regulatory classification of products derived from manure, like biochar or biocrude oil, is frequently unclear, which can restrict market access or impede their commercialization. Furthermore, strict environmental monitoring and pollution control procedures are required to address concerns about air emissions (such as particulate matter, NOx, and PAHs) from combustion or gasification processes (Bumharter, 2024; Shahzad et al., 2024; Tarafdar et al., 2025).

However, continuous advancements and cooperative research projects are making great strides in overcoming these constraints. For example, mobile and modular pyrolysis units are being developed for decentralized deployment on medium-sized farms, allowing for site-specific waste valorization and lowering transportation costs. In order to take advantage of economies of scale and shared infrastructure, demonstration projects involving public-private partnerships are testing the co-location of manure thermochemical conversion facilities with industrial symbiosis zones or renewable energy parks. In addition, global biochar quality standards (like IBI and EBC) are being standardized to boost consumer trust and regulatory acceptance. These advancements are expected to hasten the adoption of thermochemical technologies in conventional manure management, especially when paired with advantageous policy incentives like carbon credits, feed-in tariffs for renewable energy, and nutrient trading schemes (Assaye, 2024; Karanjikar et al., 2024; Shahzad et al., 2024).

New and thermochemical methods for valuing manure are a revolutionary development in sustainable livestock production. Through the transformation of a conventionally troublesome waste stream into a variety of value-added products, such as chemical feedstocks, soil amendments, and renewable energy, these technologies promote resource efficiency, economic resilience, and climate mitigation. However, a systems-level strategy that combines technological innovation with strong policy frameworks, stakeholder engagement, and customized solutions for various livestock production systems is necessary for their successful implementation. Thermochemical valorization is anticipated to become more and more important in creating future livestock systems that are low-emission and climate-smart as climate action increases and sustainability demands transform the agri-food landscape.

## 6 Precision livestock farming

A revolutionary development in animal agriculture, precision livestock farming (PLF) combines real-time biometric monitoring, artificial intelligence, and networked sensors to improve environmental stewardship, animal welfare, and production efficiency. PLF platforms make it possible for ongoing physiological, behavioral, and environmental data collection and analysis, transforming livestock operations into digitally governed systems that support well-informed individual and herd-level decision-making (Kumar et al., 2024; Ghavi Hossein-Zadeh, 2025).

PLF's emerging technologies cover a wide range of inventions. Millimeter-wave radar systems are being used more and more for non-invasive respiratory evaluations, while optical imaging methods like thermal and hyperspectral sensing are being used to identify early physiological abnormalities. Blockchain-enabled advanced traceability infrastructures make it possible to permanently record animal health, treatment records, and productivity benchmarks, guaranteeing supply chain transparency and regulatory compliance. In addition to the traditional use of identification tags, advanced sensor arrays now include decentralized edge computing units that process sensory inputs locally to enable quick reactions to events specific to a given animal, ingestible pH and temperature sensors, and 3D camera systems for posture and gait analysis (Sitaraman, 2024; Ghavi Hossein-Zadeh, 2025).

Policy initiatives that support improved biosecurity and decreased antimicrobial usage support the implementation of PLF. Sensor-based early detection systems can spot behavioral, vocalization, or mobility abnormalities that point to subclinical infections, allowing for treatments to start before clinical symptoms show up. Dynamic microclimate management is now possible thanks to predictive algorithms that use environmental factors like temperature, humidity, and airflow. This is crucial for reducing heat stress and improving air quality in confined animal housing. Additionally, machine learning models are used to regulate lighting and ventilation systems, coordinating environmental controls with resource efficiency and animal welfare standards (Himu & Raihan, 2024; Solona & Lisovyi, 2024).

Real-time spectral analysis of milk or feed is used by PLF systems in nutrition management to dynamically modify ration formulations in response to anticipated emission outputs and metabolic demands. In order to minimize environmental externalities like enteric methane and balance dietary inputs with desired production outcomes, generative AI tools are being used more and more. Automated behavioral interventions that decrease injury and enhance carcass quality can now be implemented in real time thanks to behavioral biometric systems that have progressed to the point where they can decode species-specific stress and social interactions (Akintan et al., 2025; Ghavi Hossein-Zadeh, 2025).

Digital twins, which mimic management tactics under various climatic or disease conditions, are being used by PLF to grow at the system level. These models support operations scaling while preserving system robustness. Improvements in connectivity, such as the growth of satellite-based internet services and the creation of self-powered sensors, are lowering adoption barriers in large-scale and remote operations. Consequently, a growing number of national policy frameworks acknowledge digital livestock data as legitimate documentation for environmental certification and welfare assurance. PLF metrics are starting to be incorporated into risk assessments and premium structures by sustainability initiatives and insurance companies (Arulmozhi et al., 2024; Panchadi et al., 2024).

**Fig. 5:**
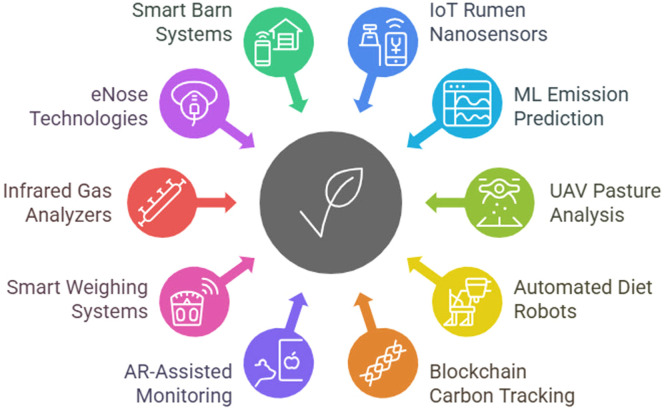
Precision livestock farming technologies for GHG mitigation in livestock.

Frontier technologies such as nanomaterial-based biosensors capable of detecting biomarkers like cortisol in sweat and autonomous robotic platforms for barn sanitation and disease control are extending the scope of PLF beyond traditional domains. These tools not only augment biosecurity but also support labor efficiency and environmental hygiene. The cumulative effect of such integration has demonstrated potential to enhance productivity while simultaneously reducing nitrogen excretion and greenhouse gas emissions, positioning PLF as a cornerstone of sustainable intensification. As the global livestock sector confronts the dual imperatives of increasing protein demand and mitigating ecological impacts, PLF offers a data-driven pathway to reconcile production goals with ethical and environmental responsibilities (Debnath et al., 2024; Ghavi Hossein-Zadeh, 2025). Precision livestock farming technologies for GHG mitigation in livestock are shown in Table 5 and Fig. 5.

**Table 5:** Precision livestock farming technologies for GHG mitigation in livestock.

Technology	Mechanism	GHG impact	GHG reduction (CO₂eq/ton)	Data requirements	Key advantages	Technical challenges	Recent references
IoT rumen nanosensors	Real-time monitoring of rumen fermentation and methane levels	Direct reduction via optimized feeding	5–20	Continuous sensor data from the rumen environment	Early detection of high emitters and it supports targeted interventions	Sensor durability, power supply, and animal welfare concerns	Ma et al. (2024b) Zhengtian et al. (2025)
ML emission prediction	Predicting methane output based on animal behavior and intake	Prevention through proactive management	10–30	High-frequency animal data (feed, behavior)	Supports precision diet formulation and emission reduction	Data quality, model generalization, and integration into systems	Basak et al. (2025) Pence et al. (2024)
UAV pasture analysis	Monitoring pasture biomass, quality, and grazing patterns	Indirect reduction through optimized grazing	5–15	Multispectral imaging, geolocation	Enhances pasture utilization and carbon sequestration	Weather dependency, regulatory constraints, and image processing	Ghavi Hossein-Zadeh (2025) Laplacette et al. (2025)
Automated diet robots	Deliver customized rations tailored to individual animal needs	Reduced enteric emissions via feed precision	15–40	Diet formulations, intake history	Minimizes feed waste, optimizes nutrient intake	Infrastructure cost, calibration complexity	Ghavi Hossein-Zadeh (2025) Kamau et al. (2025)
Blockchain carbon tracking	Immutable tracking of carbon credits and GHG metrics	Incentivizes emission reductions	Indirect/ enabling	Emission data, production records	Enhances transparency, traceability, and carbon accounting	Data verification, digital literacy gaps	Tripathy et al. (2025) Jenkins et al. (2025)
AR-assisted monitoring	Augmented reality interfaces for livestock environment control	Improved facility management reduces emissions	5–15	Sensor networks, environmental data	Enhances decision-making, real-time visualization	High-tech investment, user training, and hardware compatibility	Slimani et al. (2025) Sara et al. (2024)
Smart weighing systems	Continuous monitoring of animal weight to correlate with emissions	Indirect GHG estimation through growth metrics	3–10	Weight sensors, growth rate history	Non-invasive data collection, supports feeding decisions	Sensor accuracy, cost of implementation	Chen et al. (2025b) Hasan et al. (2024)
Infrared gas analyzers	Direct measurement of methane near animals during feeding	Accurate GHG quantification	Measurement tool (no direct reduction)	Methane concentration, feeding time data	Provides emission baselines, supports mitigation tracking	Maintenance-intensive, weather sensitivity	Ma et al. (2024) O’Connor et al. (2024)
eNose technologies	Detection of volatile organic compounds linked to fermentation	Potential methane detection proxy	3–12	Sensor arrays, pattern recognition algorithms	Early emission signal identification	Calibration requirements, data interpretation	Shi et al. (2024c) Giovinazzo et al. (2025)
Smart barn systems	Integrated environmental control systems (ventilation, lighting, …)	Reduced emissions from housing systems	10–25	Temperature, humidity, and CO₂ levels	Optimizes animal comfort, energy efficiency	High installation cost, energy backup needs	Khaowdang et al. (2025) Benni et al. (2025)

### 6.1 IoT-enabled emission monitoring systems

By allowing for the spatially and temporally resolved monitoring of GHG emissions, the incorporation of Internet of Things (IoT) technologies into livestock systems has completely transformed environmental management. These systems continuously measure the concentrations of gases like CH₄, NH₃, and H₂S, as well as particulates and microclimatic variables, using interconnected networks of low-power sensors embedded within barn infrastructures. Sensor arrays, which include optical spectrometers, electrochemical cells, and semiconductor detectors, are frequently connected to ventilation control units to enable responsive, real-time air exchange adjustments that balance emissions reduction with animal welfare (Binisha et al., 2024; Ma et al., 2024b).

Modern IoT architectures have developed into intelligent platforms that use machine learning and edge computing to accurately classify the sources of emissions. Algorithms, for example, can differentiate between methane produced by enteric fermentation and that produced by the storage of manure, improving emission inventories and guiding focused mitigation efforts. To map emission dynamics to particular production events, such as feeding cycles or waste management activities, these systems also integrate with advanced analytics, such as time-series cross-correlation. In certain applications, ground-based IoT devices are being integrated with satellite data and aerial sensors to produce geospatial emission maps for wider operational and regulatory purposes and validate emissions estimates (Guisiano et al., 2024; Tsui et al., 2024).

Systems enabled by the IoT provide closed-loop environmental control in contrast to static monitoring methods. Sensors identify changes in gas concentrations, which cause the climate control infrastructure to adapt. By doing this, pollutant spikes are lessened while ideal humidity and temperature levels are maintained. IoT platforms have grown to include unmanned aerial vehicles (UAVs) with spectroscopy equipment in feedlot or open-range environments. This allows for the high-resolution mapping of gaseous plumes and the correlation of these patterns with animal-level data, such as feeding habits or microbiome profiles (Bakti et al., 2024; Binisha et al., 2024; Saraswathi et al., 2024).

Advanced systems are now distinguished by sensor fusion, which connects pH/temperature feedback loops, reflectometry-based slurry condition assessments, and ground-level gas measurements with aerial data. When conditions surpass predetermined thresholds, these integrated systems facilitate precision interventions like automated slurry acidification. Significant potential for emission reductions exists when sensing technologies and mitigation systems are coupled, especially in the management of manure (Premalatha et al., 2024; Tsui et al., 2024).

IoT-based emission monitoring is increasingly needed in areas with climate targets, especially in areas with high climate targets, as policy developments require the use of IoT-based emission monitoring. These systems are being linked to centralized reporting platforms, often with blockchain infrastructure to ensure tamper-proof documentation. Smart contracts associated with these platforms automate compliance enforcement and allow for eligibility for environmental incentive programs. The verified outputs of IoT emission systems are being framed as economic support mechanisms, such as subsidies and carbon credit programs, that will provide direct financial incentives for data-driven mitigation, based on verified outputs of IoT emission systems (He et al., 2024; Jenkins et al., 2025).

Data integrity and security are important factors. Even under public verification frameworks, cloud-based AI platforms can securely process sensitive emissions data thanks to advancements in encryption, such as homomorphic protocols for wireless sensor networks. IoT sensor nodes are becoming more self-sufficient, running on solar arrays or microbial fuel cells, and are made to be deployed for extended periods of time in harsh conditions like open lots or slurry pits without requiring battery changes (Idris & Yassaanah, 2024).

Sensor drift and cross-interference from disinfectants or volatile organic compounds are two issues that are being addressed by next-generation sensing materials and techniques. Long-term deployments of nanostructured metal-oxide sensors with auto-calibration and selective coatings are preserving measurement accuracy. Furthermore, to reduce uncertainty in whole-farm emissions inventories, new international standards for GHG monitoring are integrating calibration procedures, data reconciliation strategies, and performance benchmarks specifically designed for livestock operations (Chakraborty et al., 2024; Tereshkov et al., 2024).

Complete decision-support systems are being created by combining digital platforms for farm planning and nutrient management with Internet of Things-based monitoring. To maximize efficiency and compliance, these systems combine emissions data with weather predictions, animal performance indicators, and waste application schedules. IoT technologies are positioned not only as data collection tools but also as crucial infrastructure in the operationalization of climate-smart livestock farming, as climate reporting regulations get more specific and enforced. Through ongoing, verifiable, and adaptive emissions management, they help farms move from reactive compliance to proactive environmental stewardship (Ilavenil & Senthilkumar, 2024; Mishra & Sonal, 2024).

### 6.2 AI-driven methane prediction models

Methane prediction in livestock systems using AI is revolutionizing the ability to track, predict, and control enteric emissions with previously unheard-of precision. Modern AI models can create predictive evaluations of methane output at the individual and herd level by utilizing multimodal data streams. These streams include remote sensing data for forage conditions, individual animal genetic and microbiome profiles, and real-time rumen telemetry. These systems provide continuous, non-invasive alternatives that are in line with operational and environmental objectives, and they are becoming more and more precise than traditional respiration chamber techniques (Rodrigues et al., 2024; Altshuler et al., 2025b).

Transformer-based neural networks and recurrent models that combine temporal, spatial, and physiological data are used in advanced AI architectures. These inputs include biometric signals like jaw movement entropy, which are obtained from muscle activity monitoring, as well as imaging and LiDAR-based systems that quantify the composition of milk and the surface characteristics of manure. Methods for machine learning, particularly those that use layers for uncertainty quantification and attention mechanisms (e.g., methane budgets for each animal over a range of periods can be generated using Bayesian dropout, which takes measurement uncertainty and biological variation into account (Hütt et al., 2024; McMeniman et al., 2025; Neethirajan, 2025).

To enable more reliable generalization, AI models are also being trained on diverse datasets derived from various breeds and environmental settings. Predictions are being refined using inputs like rumen fluid viscosity, gas diffusion rates through the dermis, and microbiome diversity obtained from 16S rRNA sequencing or swab-based diagnostics. Months of coordinated physiological and environmental data collection validate these multimodal approaches, and controlled trials compare the results to gold-standard measurements (Sabaria, 2024; Przymus et al., 2025).

Operational integration of AI-driven models into feeding systems allows for dynamic emission mitigation. For example, feed management systems now utilize predictive outputs to autonomously adjust the composition of total mixed rations (TMR), optimizing the use of methane-inhibiting additives based on anticipated emission trajectories and rumen health indicators such as protozoal motility. Reinforcement learning algorithms govern these adjustments, ensuring that productivity metrics such as milk solids or growth rates are maintained while emissions are curtailed (Akintan et al., 2024; Holmes, 2024; Parmar et al., 2024).

In order to enable verifiable carbon accounting, blockchain technology is being used more and more in conjunction with AI prediction models. Predicted emissions and genomic breeding values are now linked by smart contracts based on decentralized ledgers, offering an unchangeable and transparent foundation for the issuance of carbon credits. Emerging policy frameworks that require routine, AI-audited emission reporting and retraining of local models to account for seasonal variations in animal responses and forage composition are supported by these tools (Adigun et al., 2024; Baklaga, 2024; Shukla et al., 2025).

In modern architectures, temporal modeling is especially sophisticated. From real-time acoustic analysis of eructation events to medium-range feeding behavior tracking through automated weighing platforms to long-term indicators like body condition scoring using depth imaging, hierarchical learning systems process data at multiple temporal resolutions. The predictive power of these models is further enhanced by microbiome-based predictors, such as changes in the relative abundance of dominant bacterial taxa (Moutaouakil & Falih, 2024; Nolasco et al., 2024; Patharkar et al., 2024).

Innovation in sensors is pushing the boundaries of methane monitoring inputs. Transdermal methane flux can now be continuously measured using flexible epidermal patches made of gas-permeable nanomaterials. These patches also come with integrated thermoelectric systems that enable self-powered operation. With the least amount of disturbance to the animal, these biosensors offer high-frequency physiological emission data, adding a new dimension to AI-enabled prediction (Chakraborty et al., 2024; Domènech-Gil et al., 2024).

Agricultural communication protocols are being standardized to address interoperability issues. These days, toolkits allow AI models to easily communicate with a variety of farm equipment, such as environmental control systems, milking robots, and feed pushers. Comprehensive data integration across platforms is ensured by this harmonization. Concurrently, the safe implementation of federated learning is made possible by privacy-preserving technologies like homomorphic encryption, which permit cooperative model training on delicate genomic datasets without jeopardizing data sovereignty (Ingole & Padole, 2024; Cabrera et al., 2025). With transfer learning and domain adaptation techniques enabling model deployment in both dairy and beef systems, AI innovation is spreading across species and geographical boundaries. High predictive validity across heterogeneous production environments is now being attained by models trained on global datasets that incorporate a variety of microbiome sequences and management regimes (Han et al., 2024a; Ignesti et al., 2024).

AI and climate risk finance are bringing new resilience tools to producers, bringing new resilience tools to the table. Parametric insurance products are emerging that use differences between predicted and observed emissions as payout triggers, thereby monetizing prediction fidelity and providing economic buffers in volatile markets. These frameworks support the integration of AI models into more comprehensive sustainability finance ecosystems, linking digital methane forecasting with participation in the carbon market and environmental risk management, as well as integrating digital methane forecasting into the ecosystem of sustainability finance (Rahman et al., 2024; Xia, 2024).

AI-powered methane prediction models are an amalgam of environmental governance, data engineering, and animal science. They provide a means of achieving intelligent, adaptable livestock systems that, via proactive, data-driven management, can balance productivity with environmental constraints.

### 6.3 Automated feeding optimization

By combining cyber-physical systems, biosensor networks, and artificial intelligence, automated feeding optimization is a revolutionary development in PLF that improves animal health, environmental sustainability, and nutritional strategies. Based on real-time data from individual animals, such as physiological markers, environmental factors, and behavioral metrics, these systems dynamically modify the delivery of nutrients. Rations can be updated continuously based on feed intake behavior, digestibility metrics, and nutrient composition thanks to the combination of robotics and intelligent feed analyzers (Kaur et al., 2024; Kumar et al., 2024; Ghavi Hossein-Zadeh, 2025).

These systems' essential parts include wearable accelerometers for mastication profiling, bolus sensors for tracking rumen metabolites, and robotic feeding vehicles outfitted with near-infrared spectroscopy (NIRS) for quick feed evaluation. These datasets are interpreted by machine learning algorithms to minimize methane emissions and nitrogen excretion, predict digestive inefficiencies, and optimize amino acid balances. This process is further improved by model-predictive control mechanisms, which use metabolic flux models to guide the delivery of fermentable carbohydrates or bypass proteins based on the current health status of the rumen (Kaur, 2024; Ghavi Hossein-Zadeh, 2025).

Additional data sources are added to these architectures, including weather forecasting to prevent heat stress through proactive ration adjustments, automated body condition scoring from depth imaging, and spectral analysis of milk components for urea and protein levels. Using reinforcement learning protocols to reduce feed sorting behavior and maximize animal interaction with the bunk, leading platforms use federated learning to continuously improve feed delivery timing and frequency based on behavioral patterns identified by vision systems (King et al., 2024; Logeswaran et al., 2024; Akintan et al., 2025). Nowadays, self-regulating feed storage bins have environmental sensors that can identify levels of ammonia and carbon dioxide, two markers of spoilage risk, and release stabilizing agents like blends of organic acids when deterioration thresholds are reached. By minimizing microbial growth and preserving aerobic stability, embedded control systems increase feed shelf life and enhance the consistency of animal intake (Rani, 2024).

With international protocols being developed to standardize the validation of automated feeding systems against reference methods for crude protein and fiber digestibility, regulatory alignment is speeding up. By connecting feed formulation modifications to upstream ingredient life cycle assessments in compliance with environmental certification standards, blockchain technology guarantees the traceability of ration adjustments for carbon footprint reporting (Sheriff & Aravindhar, 2024).

Advanced encryption protocols and other cybersecurity measures safeguard the integrity of on-farm data and proprietary feed formulations. Modern platforms also incorporate predictive maintenance systems, which use failure mode analysis and digital twins to predict component wear and avoid mechanical downtime. These systems can now simulate operational scenarios to improve long-term ration planning, animal flow, and feed delivery scheduling (Bissadu et al., 2024).

In the direction of customized feeding plans, precision delivery techniques are progressing. Personalized nutrition is made possible by biosensor-guided analyzers and sorting gates based on magnetic resonance, which match feed ingredients to each animal's current metabolic state. In response to rumen telemetry, smart micro-dosing systems release encapsulated additives, improving nutrient absorption and facilitating focused health interventions during crucial physiological times, such as the transition phase in dairy cattle (Arora et al., 2024; Maigaard et al., 2024; Su et al., 2024).

Environmental feedback is being directly incorporated into ration formulation by emerging technologies. For instance, the addition of dietary lipids or methane inhibitors is controlled by emissions data obtained from barn exhaust systems, enabling the real-time reduction of greenhouse gas emissions related to the production of milk or meat. The implementation of environmentally optimized feeding strategies relies heavily on these closed-loop feedback systems (Akintan et al., 2024, 2025; Holmes, 2024).

From an economic perspective, sophisticated platforms combine market analytics and feed management, creating least-cost ration scenarios based on real-time commodity data. With the aid of these resources, producers can manage price fluctuations without sacrificing nutritional quality. Continuous innovations provide long-term dependability in dusty or humid environments by addressing issues like sensor fouling through self-cleaning mechanisms (Mijić et al., 2024).

Hyperautomated feeding systems are developing into self-sufficient nutrition centers at the forefront of this field. These platforms use unmanned aerial vehicles and customized feed packets labeled with animal-specific identifiers to sync robotic milking schedules with therapeutic feeding regimens. In a data-centric agricultural paradigm, these integrative solutions align livestock performance with sustainability, profitability, and welfare, and they represent the next generation of smart feeding systems (Vlaicu et al., 2024; Ghavi Hossein-Zadeh, 2025).

### 6.4 Drone-based pasture management

By providing high-resolution, real-time evaluation of forage systems via sophisticated remote sensing and autonomous navigation, drone-based technologies are revolutionizing pasture management. These aerial systems, which are outfitted with thermal fusion cameras, synthetic aperture LiDAR, and hyperspectral imaging, can monitor vegetation indices, produce accurate biomass estimates, and evaluate canopy structure while taking plant health and moisture content into consideration (Ogungbuyi et al., 2024; Santos et al., 2024).

To maximize pasture productivity and nutrient management, contemporary platforms combine artificial intelligence and machine learning models trained on various vegetation indices. These drones facilitate variable-rate applications of inputs like fertilizers or seed coatings by mapping spatial variability in soil fertility and forage composition. In order to create site-specific management zones and predictive maps for pasture rotation, reseeding, or supplement application, onboard edge computing modules frequently process real-time spectral data gathered by unmanned aerial vehicles (UAVs) (Arısoy & Açıkgözoğlu, 2024; Meshram et al., 2024).

Drone data and precision input deployment equipment can be automatically linked thanks to the further integration of advanced systems with decision-support platforms. This makes it possible to dynamically zone grazing areas based on animal distribution, forage availability, and anticipated dry matter intake. Protocols for paddock allocation enabled by blockchain are being developed to improve traceability and allow for real-time rotational grazing strategy adjustments (Guebsi et al., 2024).

In addition to mapping, autonomous UAVs aid in pasture restoration by using vision-guided delivery systems to precisely disperse seeds and inject soil amendments into degraded microsites. To enhance germination and nutrient efficiency, seed treatments—such as microbial inoculants or biochar coatings—are tailored to the site's characteristics. At the same time, drones that have thermal imaging capabilities can help monitor the health of animals by spotting early indicators of problems with movement or physiological stress through minute temperature variations (Castro et al., 2024; Ghavi Hossein-Zadeh, 2025).

A thorough understanding of animal-pasture interactions is now possible thanks to the growing integration of drone-based data streams with livestock wearable technologies like GPS collars and ingestible biosensors. Predictive models can forecast forage growth, evaluate pasture use efficiency, and assist with harvest planning in precision grazing frameworks thanks to these multisource data systems (Parsons et al., 2024; Qazi et al., 2024).

Beyond-visual-line-of-sight (BVLOS) drone operations are now allowed by regulatory changes, provided that specific safety measures are followed, such as using radar-based obstacle detection and encrypted communication standards to guarantee operational security. Lightweight, self-repairing composites and energy-dense battery technologies are two examples of concurrent material advancements in drone construction that are increasing flight duration and payload capacity, allowing for greater coverage and more frequent data collection (Baguer et al., 2024; Politi et al., 2024).

UAV systems use federated learning models and compression algorithms to manage the large amount of imagery and telemetry data generated, allowing forage prediction models to be calibrated regionally while maintaining privacy. These models support yield forecasting, carbon accounting, and benchmarking pasture conditions by utilizing site-specific features and historical data (Dwarampudi & Yogi, 2024; Santos et al., 2024).

By facilitating carbon sequestration modeling and methane flux estimation, integration with environmental monitoring tools improves drone-based pasture management. Custom gas sensors and LiDAR-based root biomass estimation enable spatially explicit carbon quantification, facilitating the creation of carbon credits that encourage participation in climate mitigation programs (Bazzo et al., 2024; McQuilkin et al., 2024).

Standardized communication protocols are being used to address the interoperability issues between drone platforms and legacy farm systems, allowing UAVs to communicate with soil monitoring infrastructure and irrigation controllers that are currently in place. In order to preserve system integrity and location accuracy during autonomous field operations, new encryption standards, such as quantum-resistant key management, also protect secure data transmission (Abdullin & Sungatullin, 2024; Peddibhotla et al., 2024).

Economic evaluations of drone-enabled pasture optimization show significant gains in metrics related to milk or meat production, nutrient management, and feed efficiency. These technologies are therefore becoming more popular in integrated livestock systems, and more developments in autonomous habitat structuring for biodiversity enhancement, real-time pollination support, and soil microbiome sensing are expected (Alanezi et al., 2022; Mahale et al., 2025). Leading platforms are now integrating solar-powered herbage meters and drone swarms in tandem with subsurface sensors into closed-loop pasture systems. These configurations are intended to support the delivery of ecosystem services, preserve ideal sward structure, and aid in the production of climate-resilient fodder (Ranjan et al., 2025).

### 6.5 Blockchain and immersive technology for emission monitoring and traceability

The idea of precision livestock farming in terms of emissions monitoring, traceability, and sustainability verification may be disrupted by the combination of blockchain technology and immersive digital technologies, especially augmented reality (AR), virtual reality (VR), and digital twins. These technologies will offer creative frameworks for developing transparent, accountable, and reflective livestock production systems in addition to measurement solutions as the livestock industry faces increasing pressure to comply with international climate agreements and market demands to reduce their environmental impact. In order to inform on-farm decisions and the larger climate governance framework, it is imperative that emissions reporting be moved from episodic, estimation-based reporting to continuous, real-time, and verifiable data feeds (Arulmozhi et al., 2024; Shulzhyk et al., 2024; Ghavi Hossein-Zadeh, 2025).

Along the livestock value chain, the blockchain provides a secure and transparent way to gather and exchange pertinent emissions-related data because it functions as a decentralized and unchangeable ledger system. When data on, for instance, methane emissions from enteric fermentation, nitrous oxide emissions from manure management, and energy use related to livestock housing is registered into an innovative layer, it is ultimately an enduring, immutable record, and the information will never cease to exist. This is how blockchain differs from traditional databases and systems, which are susceptible to manipulation and centralized control. Real-time traceability and auditability of the complete chronology of livestock production events are made possible by the time stamping and cryptographic linking of each record to the one before it. Since reliable carbon markets, certification programs, and sustainability labels account for a large portion of the reported information and data's credibility, the immutability component is crucial when making claims about climate mitigation. For instance, a blockchain system could store emissions data from sensors on cattle as well as the pertinent metadata, such as feed, health, and manure management procedures. This type of traceability serves as the foundation for both scientific support for climate-smart livestock management and adherence to environmental regulations (Assis & Batista, 2024; Kumari & Agarwal, 2024).

Additionally, smart contracts, completely autonomous protocols that automate predefined behavior based on specific conditions being met, can be created using blockchain technology. When it comes to livestock emissions, smart contracts could be developed to automatically give farmers carbon credits when their methane emissions surpass predetermined minimum levels or when they present proof of sustainable practices, like rotational grazing or dietary amendments (methane inhibitors) verified by sensor systems. Smart contracts speed up payments to farmers, reduce the possibility of disputes, and simplify the administrative costs of payment related to more traditional incentive programs. Furthermore, smart contracts can enhance decentralized governance structures in supply chains or cooperatives to encourage more equitable distribution of project benefits or to support group climate action. Thus, blockchain is more than just a database; it is a dynamic system that encourages behavior shifts toward low-emission agricultural systems and co-creates multisystem engagement (Jawalkar et al., 2024; Kumari & Agarwal, 2024).

Emissions monitoring is made more interactive and visually appealing by immersive technologies like AR and VR. When navigating the agricultural space, farmers can view emission sources, animal behavior indicators, or environmental conditions in real time thanks to AR devices like smart glasses or head-mounted displays that can place situational context in their field of vision. For instance, a livestock manager wearing an AR headset could see his real-time estimates of methane emissions from above each animal, get notifications when the barn's ammonia levels rise, or see the ventilation system's airflow characteristics. When data and the physical environment are combined, farmers can make decisions more quickly and intelligently and have better situational awareness, especially in complex or large-scale agricultural systems (Shulzhyk et al., 2024; Sara et al., 2024). Simultaneously, VR systems can offer immersive training environments that allow veterinarians, technicians, and producers to compare the economic and environmental trade-offs of various management interventions and simulate a variety of emission-reduction scenarios without interfering with real-world agricultural operations. By simulating particular farm layouts, configurations, and conditions, these virtual environments enable scenario modeling and hands-on learning related to the different interventions, such as adjusting housing ventilation, integrating biofiltration systems, or optimizing manure management timings. VR technology helps reduce emissions and build capacity with regional and local stakeholders, including those who are unfamiliar with climate-smart technology. It does this by making sustainability training more accessible and engaging (Amshavalli et al., 2024; Shulzhyk et al., 2024).

The development of digital twins, virtual copies of real assets or systems that are updated in real time with data, is one of the most exciting developments that will take place as this technology converges. A digital twin in livestock management could depict a single animal, a herd, or a whole business, reflecting interactions with compromises in emissions, growth, and health performance, and feed intake. Considering information from sophisticated IoT-enabled sensors (e.g., thermal cameras, motion detectors, gas analyzers, and rumen boluses), the digital twin could simulate the effects of various procedures (e.g., feeding, genetic, and facility) on emissions levels in a variety of production mixes and environmental changes. Instead of testing mitigation activities in a real-world setting, these simulations would enable proactive action through decision-making that validates them in a digital setting. When paired with blockchain technology, a digital twin can also serve as a permanent, fully validated record of system behavior, providing a reliable closed loop of accountability, validation, and measurement (Arulmozhi et al., 2024; Galar & Kumar, 2024).

Increased consumer demand for sustainability and transparency across food systems is being met in part by blockchain and immersive technologies, in addition to gains in operational efficiency and regulatory compliance. More consumers are interested in the production process of their food, including whether it was ethical, environmentally sustainable, and climate-friendly. Blockchain makes this possible by using digital traceability to connect emissions data and indicators of animal welfare to the final product. By scanning the QR code on a milk carton, for instance, you can view the product's journey, including the herd from which it originated, the feeding schedule, the housing conditions, and the emissions per liter of milk, all supported by sensor readings that are stored on a blockchain ledger. Transparency increases consumer confidence and trust, gives consumers more options in the market, and gives producers who invest in sustainable endeavors a competitive edge (Chinnasamy et al., 2024; Gomes et al., 2024; Vignesh et al., 2024). Furthermore, the application of immersive technologies and blockchain has important ramifications for national and international climate governance. National emission estimates submitted to international organizations such as the United Nations Framework Convention on Climate Change (UNFCCC) can be made more accurate and detailed by applying blockchain-verified data to tiered GHG inventory systems. These technologies link individual producer actions to collective climate pledges by coordinating on-farm practices with global climate reporting systems. Additionally, these technologies facilitate cross-border data exchange and align sustainability certifications in international trade by supporting standardized and interoperable digital platforms (Jenkins et al., 2025; Jiang et al., 2024b).

Nonetheless, the path to widespread adoption presents several hurdles. The needed technological leverage for systems based on blockchain and other immersive systems, a reliable internet connection to the edge (computing), and storage capabilities on the cloud, is often a disparate resource in many low- and middle-income countries. Issues surrounding data ownership, privacy, and cybersecurity should also be addressed through a clear governance structure and on equitable terms across stakeholders. Current blockchain models consume excessive amounts of energy (for example, by relying on a proof-of-work model), which raises concerns about environmental impact, but new consensus algorithms (e.g., proof-of-stake and hybrid models) are under development to reduce the energy footprint. Financial restrictions, including initial investment costs and the provision of skills training, may inhibit uptake among smallholder or resource-poor farmers, demonstrating the need for inclusive policies and appropriate support structures (Al Meslamani, 2024; Andreoulaki et al., 2024).

The integration of blockchain and immersive technologies in emissions monitoring and traceability represents a watershed moment in precision livestock farming. The potential to collect and apply emissions data in real-time, transparently, and verifiably provides an unparalleled level of integrity and responsiveness in climate change mitigation efforts. The value of the technologies is their ability to connect on-farm activities not just with market opportunities but also with regulatory frameworks and consumer demand, which makes them significant partners in the evolution of sustainable, low-carbon livestock systems. Moreover, with ongoing technological development and existing barriers to adoption, these innovations will become core features of the future of climate-smart agriculture, leading to new ways to measure, manage, and value emissions in the world's livestock production.

## 7 Renewable energy integration

From being supplemental power sources, the incorporation of renewable energy technologies into agricultural systems has advanced to become an essential element of contemporary farm operations. Intelligent hybrid energy systems that dynamically balance distributed generation with on-farm energy demands throughout the phases of cultivation, processing, and storage have aided in this evolution. In modern implementations, advanced battery storage systems that use repurposed electric vehicle batteries, anaerobic digestion units that process agricultural residues, and microgrid controllers enhanced with machine learning algorithms optimize energy flows between solar photovoltaic installations. While adjusting to variations in water consumption for irrigation and energy-intensive processes like precision fermentation or controlled environment agriculture, these systems preserve energy efficiency and dependability (Karanjikar et al., 2024).

Agrivoltaic systems, which permit simultaneous crop production under semi-transparent solar modules, are one example of how renewable energy technologies can be seamlessly integrated with agricultural infrastructure thanks to modern standards for distributed energy resource interoperability. In order to improve participation in carbon offset markets, these integrations frequently work in conjunction with linked irrigation networks and use blockchain technology to validate renewable energy certificates. Agricultural processing facilities' energy needs are predicted by predictive analytics, which is aided by cloud and edge computing architectures. This allows for scheduling that is coordinated with demand response programs and aligned with renewable generation profiles, which enhances grid stability (Bell et al., 2024; Karanjikar et al., 2024). This progress is aided by cutting-edge advances in material science, such as nanogenerator technologies integrated into greenhouse structures that capture kinetic energy from environmental elements like rainfall, boosting microgrid resilience and supplying additional renewable energy. The resilience of decentralized farm-based energy systems has been highlighted by these developments, which have shown excellent performance even in the face of severe weather (Kanchana et al., 2025).

With revised guidelines and standards that establish challenging goals for agricultural businesses to become self-sufficient in renewable energy, policy frameworks are hastening the adoption of on-farm renewable energy. Verification of renewable energy contributions is facilitated by satellite-based monitoring technologies, which offer transparent and auditable data for regulatory compliance and emissions reduction program participation. Economic studies now show promising investment prospects for integrated renewable systems in agricultural settings, with revenue streams diversified through ancillary grid services and participation in virtual power plants (Karanjikar et al., 2024; Nazarov et al., 2024).

There is great potential in the implementation of renewable energy technologies into livestock systems, as they can help in reducing carbon footprints; they are more feasible and efficient, but these are context-specific. The performance and suitability depend on the size of the farm, the geographic location, climatic conditions, and the availability of resources such as solar irradiance, wind regimes, and the nature of manures. Economies of scale and readiness of infrastructures might be advantageous to massive, geographically concentrated operations, but space, technical, and logistical restrictions may be imposed on smallholder or mixed farming systems. Such variability should be identified to prevent the overgeneralization of results and to ensure that the renewable energy solutions can be properly adapted to local production conditions (Everaert et al., 2023; García Alvaro et al., 2023; Costantini et al., 2024).

The adoption of renewable energy in livestock operations is largely determined by an overwhelming economic factor. The requirements of high investments of capital, continuous maintenance expenses, and uncertainty about the payback periods may constrain implementation, especially in resource-limited environments. There might be significant savings in their long-term operation and the emission rate, but the initial financial risk and access to credit can be impediments to uptake. These obstacles underscore the role of policy incentives, collaborative designs, and scalable systems in enhancing the economic sustainability of various production systems (Costantini et al., 2024; Karanjikar et al., 2024).

The success of renewable energy integration will highly rely on its integration with the current farm systems, such as manure handling systems, feed storage areas, housing, and operational processes on a daily basis. Adapting energy technologies to the existing systems might be technically difficult and may need significant redesign or more work. Having functional congruence between energy generation, energy storage, and energy demand at the farm is crucial in ensuring efficiency and less disruption in the production processes (Karanjikar et al., 2024; Benni et al., 2025).

In addition to the mitigation of emissions, the integration of renewable energy has a number of co-benefits that can improve the resilience and sustainability of livestock systems. These include enhanced on-farm energy security, less reliance on fossil fuels, and more operational power amidst fluctuating energy markets. Biogas systems, specifically, can be used to boost nutrient recycling by utilizing digestates, which can help to connect energy generation and nutrient management and strengthen the idea of the circular economy. These co-benefits make renewable energy strong in its value proposition (Karanjikar et al., 2024; Taye et al., 2025).

Regardless of their merits, renewable energy systems are connected with technical and environmental constraints that should be mentioned. The intermittency of solar and wind energy may make energy difficult to plan and necessitate some provision of backup capacity or grid connection. The technical complexity, land-use needs, and maintenance needs can also further limit adoption. In other instances, trade-offs in energy production and agricultural use may be realized in light of competition over land or resources, and thus the importance of proper system design and impact assessment (Hassan et al., 2023; Obuseh et al., 2025).

The latest trends in energy storage, microgrid design, automation, and smart energy management systems can provide valuable opportunities to address most of these limitations. The energy flows can be optimized by using integrated energy management platforms, the supply and demand curves can be balanced, and the reliability of the systems can be increased. This is especially applicable to decentralized livestock systems, whose efficacy and durability can be enhanced with the help of flexible and adaptable energy solutions (Al Smadi et al., 2025).

Integration of renewable energy should be considered as part of a complementary aspect of the large multi-pronged mitigation efforts, rather than a solution on its own. Combined with technologies in manure management, various dietary interventions, and genetic optimization, it can enhance total emission abatement and enhance the efficiency of resources. The systematic application in these areas will provide system-level optimization and prevent the transfer of environmental loads between the production processes (Gupta, 2025; Symeon et al., 2025).

Operational effectiveness and indirect impact on product quality can be achieved by means of the renewable energy systems through better environmental regulation, feed conservation, and a processing environment. A stable energy supply, along with the best housing conditions, cooling systems, and feed management, can contribute to the welfare and productivity of animals. Viewing these operational and quality-related impacts can guarantee that the integration of renewable energy should be assessed as an environmental intervention, in addition to being one of the factors affecting the performance and integrity of the whole farm (Karanjikar et al., 2024; Taye et al., 2025).

In the context of larger decarbonization initiatives, emerging cyber-physical infrastructures position farms as active energy contributors, turning them from passive consumers into prosumers. Pilot projects have shown that agricultural facilities can supply the grid with fast-response energy resources, highlighting agriculture's crucial role in creating resilient, climate-smart energy systems (Salaria & Rakhra, 2024). Renewable energy integration strategies to mitigate GHG emissions in livestock are presented in Table 6.

**Table 6:** Renewable energy integration strategies to mitigate GHG emissions in livestock.

Technology	Mechanism	GHG reduction (ton CO₂e/yr)	Energy output	Key advantages	Integration challenges	Co-benefits	Implementation timeline	Policy dependency	Recent references
Biogas digesters	Anaerobic decomposition for methane capture	30–120	Heat, electricity	Waste utilization	Feedstock consistency	Nutrient recovery	Short-term (<3yrs)	Low	N’guessan et al. (2025) Akash et al. (2025)
Solar photovoltaic integration	PV panels for barn power	10–40	Electricity	Low maintenance	Intermittency issues	Reduced heat stress	Short-term	Medium	Pal et al. (2025) Murali et al. (2024)
Manure-to-biofuel conversion	Thermochemical fuel production	20–80	Liquid fuel	High energy density	Pre-treatment needs	Waste volume reduction	Medium-term (3–7yrs)	High	Munir et al. (2023)
Wind turbine hybrids	On-farm wind energy generation	15–60	Electricity	Land multifunctionality	Site-specific winds	Rural electrification	Medium-term	Medium	Bouregba et al. (2024) Everaert et al. (2026)
Algal photobioreactors	Algae-based CO₂ capture	15–50	Biomass	Nutrient recycling	Water/energy intensive	Animal feed production	Long-term (>7yrs)	High	Akbar et al. (2026) Li and Yao (2024)
Biomass gasification	Thermal syngas production	30–90	Syngas, electricity	High efficiency	Tar management	Ash byproduct utilization	Medium-term	Medium	Chuayboon and Abanades (2024) Qian et al. (2024)
Solar water heating	Thermal water heating	5–15	Thermal energy	Simple technology	Weather dependence	Reduced propane use	Short-term	Low	Hernandez-Robles (2025) Eze et al. (2024)
Agrivoltaics	Dual land-use PV systems	10–35	Electricity	Enhanced land productivity	Shading management	Microclimate regulation	Medium-term	High	Krexner et al. (2024) Mouhib et al. (2024)
Microbial fuel cells	Bioelectrochemical conversion	5–25	Electricity	Simultaneous wastewater treatment	Low power density	Pathogen reduction	Long-term	High	Niu et al. (2024) Cui et al. (2025)
Geothermal heat pumps	Ground-source climate control	15–45	Thermal energy	Year-round operation	Drilling costs	Reduced ventilation needs	Medium-term	Medium	Lee et al. (2025) Han et al. (2024b)
Hydrogen electrolysis	Water splitting for H₂ fuel	10–25	Hydrogen	Zero operational emissions	Storage challenges	Fertilizer production potential	Long-term	High	Elegbeleye et al. (2025) Hoshino (2025)
Biochar production	Pyrolytic carbon sequestration	15–50	Heat	Soil improvement	Feedstock variability	Water retention enhancement	Medium-term	Medium	Kim et al. (2025) Nazim et al. (2025)
Hydrothermal liquefaction	Wet waste to bio-crude	30–90	Bio-crude oil	High moisture tolerance	High-pressure requirements	Pathogen elimination	Long-term	High	Mkilima et al. (2025) Chaudhary et al. (2024)

### 7.1 Biogas-to-energy conversion systems

In order to process various agricultural waste streams efficiently, modern biogas-to-energy systems have developed into integrated biorefinery platforms. These systems maximize methane production from a variety of substrates, including liquid dairy manure, solid poultry litter, and lignocellulosic residues like vine prunings, by utilizing sophisticated anaerobic digestion configurations, such as multi-stage thermophilic and mesophilic digester designs. Maximizing organic matter degradation and biogas yields is made possible by advancements in real-time process monitoring that use mid-infrared spectroscopy and machine learning to precisely control important operational parameters like hydraulic retention times and organic loading rates (Bumharter, 2024; Akshaya and Selvasembian, 2024).

Dual-phase digestion strategies are becoming more and more integrated into modern systems. In these systems, methanogenic phases use enriched methanogen consortia, while acidogenic stages speed up hydrolysis, frequently through targeted bioaugmentation with specialized microbial strains. These setups are enhanced by *in-situ* biogas upgrading technologies that use membrane-based separation to create highly pure biomethane that complies with international standards and can be injected into the grid (Chatterjee & Mazumder, 2024; Alipoursarbani et al., 2025).

Modern facilities use fuel cell technologies that can convert biogas into electricity with remarkable efficiency, providing significant energy outputs concerning feedstock volumes, in addition to conventional combined heat and power applications. These systems' integrated nutrient recovery processes help treat wastewater and convert digestate into slow-release fertilizers, improving environmental and economic sustainability overall (Madheswaran et al., 2024).

The performance and dependability of biogas systems have been greatly enhanced by recent developments in materials science. Examples include self-healing concrete structures that increase digester lifespan by self-repairing microcracks and membranes made with cutting-edge nanocomposite materials to achieve highly effective hydrogen sulfide removal. The efficiency of the system is further increased by smart gas storage solutions that use predictive control algorithms to dynamically modify gas storage and pressure to match grid demand (Karlsen & Dinamarca, 2024).

New technologies like microbial electrosynthesis open up new avenues for digestate valorization by generating organic acids and biohydrogen, and integrated carbon capture processes enhance the climate benefits of biogas systems by converting leftover carbon dioxide into stable mineral forms. Engineered methanogens and other improved microbial consortia are increasing the variety of feasible feedstocks by making it possible to convert lignocellulosic materials that were previously underutilized (Thakur et al., 2024).

Several revenue streams from energy generation, fertilizer co-products, waste management services, and involvement in renewable energy markets enhance the economic viability of contemporary biogas-to-energy systems. Secure automation systems for operational supervision support comprehensive monitoring technologies that use elemental analysis and hyperspectral imaging to guarantee feedstock quality control and adherence to environmental regulations (Nnadikwe et al., 2024).

As these technologies mature, biogas facilities are increasingly recognized as valuable grid resources, capable of providing ancillary services such as frequency regulation. Life cycle assessments confirm the potential of these systems to deliver net-negative carbon outcomes through effective carbon sequestration in digestate applications, underscoring their strategic role in advancing agriculture toward a sustainable, post-fossil energy future (Zhang et al., 2024).

### 7.2 Solar-powered barn technologies

Solar technology integration into barn infrastructure has progressed to include multipurpose systems that maximize animal welfare, increase operational sustainability, and produce renewable energy all at once. Building-integrated photovoltaics (BIPV), which are laminated within structural insulated panels and function as efficient solar energy harvesters and thermal insulation, are now a common feature of modern barn designs. Bifacial solar arrays, which are positioned above livestock areas and use surface reflectance to improve energy capture, complement these innovations. Additionally, these installations' adjustable shading systems help reduce heat stress in animals during times of high solar radiation (Frontini, 2024).

Phase change material composites in conjunction with solar thermal systems are being used more and more to stabilize barn temperatures indoors. These systems reduce the need for energy-intensive mechanical cooling by maintaining a stable thermal environment by storing thermal energy during the day and releasing it during cooler times. Barn energy efficiency is further improved by advanced coatings on external panels that are made for high mid-infrared emissivity. These coatings allow for efficient nocturnal radiative cooling (Liang et al., 2024).

The implementation of intelligent control platforms that synchronize photovoltaic generation with the fluctuating power demands of barn equipment, including feed mixers, ventilation systems, and milking robots, has advanced energy management in solar-powered barns. These platforms, which are backed by highly efficient solid-state transformers that control power flows to maximize energy use, use sophisticated algorithms for real-time demand response. Hybrid energy storage systems buffer solar energy for constant availability even during times of low sunlight by combining long-duration flow batteries with fast-response lithium-based batteries (Falope et al., 2024; Mangate et al., 2024).

The resilience of solar barns is greatly enhanced by advancements in material science. Flexible solar fabrics can adapt to non-linear roof geometries, increasing deployment options across a variety of barn architectures, and self-cleaning photovoltaic coatings reduce dust and debris to maintain high light transmittance. Sensor networks for real-time structural load monitoring can be powered autonomously by embedded energy harvesters in structural elements, improving safety during situations like severe snowfall (Alqarni et al., 2024; Xavier & Malar, 2024).

By collecting runoff that can be filtered and used again on the farm, rainwater harvesting systems combined with solar panel arrays further enhance barn sustainability. These systems use cutting-edge filtration media that can eliminate microbiological pollutants, decreasing the need for outside water sources and operating expenses (Salleh et al., 2024).

Regulatory frameworks are increasingly recognizing the need for safe and efficient solar barn systems, including electrical safety, microgrid integration, and equipment certification. Advanced analytics platforms are now combining high-resolution weather forecasts with real-time monitoring of animal thermal signatures to predict ventilation and environmental conditions, and indirectly helping reduce methane emissions by optimizing manure management to reduce methane emissions (Ajayi et al., 2024).

Economic studies show that solar-powered barn investments are feasible, especially when paired with cost-sharing plans, accelerated depreciation schedules, and renewable energy incentives. The multipurpose potential of these installations is further highlighted by emerging solar-powered robotic technologies, such as precision feeding systems and autonomous disinfectant sprayers. Leading-edge research is investigating multipurpose energy-glass technologies that can produce solar energy and emit light that deters pathogens, enhancing barn biosecurity and paving the way for climate-smart, carbon-neutral livestock housing solutions (Verma & Goswami, 2024).

### 7.3 Manure-to-biofuel pathways

From conventional anaerobic digestion, manure-to-biofuel technologies have developed into integrated biorefinery systems that can transform livestock waste streams into a wide range of valuable energy products. Cascade thermochemical and biochemical treatments are used in these sophisticated processes to optimize resource recovery and energy density. In order to break down fibrous structures, systems usually start with thermochemical pretreatments like steam explosion. Next, complex carbohydrates are hydrolyzed using specially designed enzyme cocktails to produce fermentable sugars. Cellulosic ethanol is produced from manure-derived hydrolysates through the efficient simultaneous saccharification and fermentation made possible by yeast strains that have been engineered to assimilate a variety of sugar monomers. The leftover lignin fractions are sent to pyrolysis reactors to produce bio-oil, which can subsequently be refined through catalytic processes to produce renewable fuels that satisfy global fuel regulations (Chuenchart et al., 2024; Pandey & Sharma, 2024).

Other conversion pathways include hydrothermal liquefaction, in which wet manure is subjected to high temperatures and pressures to convert organic matter directly into biocrude for conversion to renewable diesel or aviation fuels. These systems integrate nitrogen as ammonium-based fertilizers and high-purity mineral precipitates as nutrient recovery loops, allowing circular nutrient management, and capturing nitrogen as ammonium-based fertilizers and recovering phosphorus as high-purity mineral precipitates. Regulations have become more lenient on manure-based biofuels as advanced renewables, and they can be used to contribute to climate and energy targets under national and international directives. These systems are supplemented by blockchain-based mass balance tracking platforms, which ensure robust carbon accounting and compliance with sustainability certification schemes (Feng et al., 2024; Gronwald & Wang, 2024).

New developments are also being made in gasification pathways, which use fluidized bed technologies to turn digestate from manure into syngas that is high in carbon monoxide and hydrogen and can be used to power high-efficiency solid oxide fuel cells. The production of lipid-rich biomass for biodiesel can be facilitated by rerouting the carbon dioxide produced during gasification to algal photobioreactors. The oxidation of volatile fatty acids from manure fermentation and the electrochemical reduction of carbon dioxide can be coupled to produce higher-value liquid biofuels like butanol through the use of bioelectrochemical systems with specialized microbial communities, which further expands the possibilities (Abouemara et al., 2024; Chanthakett et al., 2024).

In order to improve system performance and economic viability, material innovations are essential. Selective permeability in nanostructured membranes lowers operating costs by enabling solvent recovery and reuse in pretreatment procedures. Long-term high throughput is maintained by self-cleaning reactor designs that incorporate ultrasonic systems to prevent fouling. Real-time process optimization is made possible by digital twin models calibrated with spectroscopic data, which enhances biofuel yields and maintains product quality (Rastogi et al., 2025).

The regulatory environment is becoming more supportive of closed-loop manure-to-biofuel systems and provides incentives for methods that lower methane emissions or sequester carbon through the application of biochar. The possibility of multi-product valorization, such as carbon offsets, recovered nutrient sales, and renewable fuel credits, boosts economic viability. To ensure that the production of biofuel satisfies safety and environmental regulations, enhanced security protocols use elemental analysis and hyperspectral imaging to identify and eliminate contaminated feedstocks (Borthakur, 2025).

The scalability of these technologies is demonstrated by large-scale deployment demonstrations; integrated facilities are anticipated to transform significant amounts of manure into liquid biofuels and renewable natural gas. In order to further reduce lifecycle greenhouse gas emissions, agreements between research institutions and industry partners are fostering innovation in the direction of net-negative carbon energy systems, which incorporate direct air capture technologies. Manure valorization is positioned to make a substantial contribution to sustainable aviation fuel supplies as certification frameworks for aviation biofuels made from manure gain international recognition, turning livestock operations from net emitters into hubs for the production of renewable energy (Magnolo et al., 2024).

### 7.4 Emerging and hybrid renewable energy systems

Recent advances in renewable energy technologies are changing the prospects for sustainable forms of energy included in livestock systems, and are especially innovative and versatile in combination with hybrid technologies, beyond just solar or wind power. The unique combinations create multiple simultaneous and interdependent opportunities to reduce GHG emissions as well as increase energy independence and efficiency from a farm perspective. Of these waste-based energy opportunities, microbial fuel cells (MFCs) represent a novel and state-of-the-art technology for the treatment of animal waste while producing energy through electrogenic bacteria. Acting as a sponge as it converts the chemical energy found in organic substrates into electrical energy, MFC can be considered a new low-carbon process for waste management and energy generation; however, its scalability and ability to last over time under field conditions are areas of ongoing research (Alwahab et al., 2024; Nakayasu et al., 2024).

Hydrogen electrolysis, using on-farm renewables (solar or wind) to provide power, has become an increasingly popular method of producing green hydrogen. Because hydrogen can be an energy carrier or be used in fuel cells to create energy to power farm machinery or provide back-up energy, and potentially used in livestock systems for seasonal energy storage and decarbonizing auxiliary energy uses, hydrogen electrolysis is a technology that warrants examination. The energy losses, as well as the high capital cost of electrolyzers, create barriers to adopting hydrogen electrolysis; therefore, innovations and policy support are needed to develop and improve cost-competitiveness to support adoption at the farm scale (Saravanan et al., 2024).

Geothermal heat pumps are another feasible option for renewable thermal energy in livestock production, and they are especially viable in temperate and cold climate regions. Geothermal heat pumps take advantage of the generally stable ground temperature so that energy or heat can be added to the livestock housing, or the environment can be cooled (when required). The geothermal heat pump reduces reliance on fossil fuels for climate control, which has benefits to reduce carbon emissions as well as improve animal welfare and productivity. The feasibility of geothermal systems is site-specific and requires unique geological conditions and professional installation, which may be a barrier for smallholders in terms of logistics and economics (Han et al., 2024b; Salhein et al., 2024).

Hybrid systems combining two or more renewable technologies are emerging with great potential for optimized energy generation and reliability. For example, pilot hybrids with solar and wind equipped with battery storage are being used on livestock farms to supply a smooth, self-sufficient, and reliable source of power to coincide with the variability of renewable energy generation. Hybrid solar and wind systems, in particular, could have a widespread impact in off-grid or remote livestock operations where energy sources and infrastructure are limited, and when accompanied by AI-powered energy management software, these systems can optimally and responsively combine energy methods (Bensalmi et al., 2024; Seidu et al., 2024).

Another emerging hybrid technology includes biochar-enhanced biogas systems. In biochar-enhanced systems, biochar (developed by the pyrolysis of agricultural residues or manure) is added to anaerobic digesters to accelerate microbial metabolic activity, improve methane generation, and to once again, facilitate carbon sequestration when applied to soils or used as animal bedding. The dual functioning has both a renewable energy component and provides for long-term carbon sequestration. A different and related strategy to biochar addition to anaerobic digestion is thermal gasification, which converts dry biomass or dry manure solids to syngas through thermochemical means. Syngas can be used to generate electricity and/or heat. While gasification is considerably more energy-intensive than anaerobic digestion, it does provide for better throughput and can utilize a wide range of feedstocks, making it possible for larger CFUs to apply these processes (Delpitiya & Ariyarathna, 2024; Ngo et al., 2024).

Systems of algae-based bioenergy (e.g., algal photobioreactors) are investigated to convert nutrient-rich effluent from animals into biofuel biomass, which can not only reduce the amount of nutrients entering waterways and lakes but could also become carbon-neutral or even carbon-negative energy production systems if CO₂ capture and utilization technologies are developed and integrated into the process. Despite the potential ecological and environmental benefits of algae-based energy systems, these systems are still in the early stage of development and require considerable infrastructure, controlled algae cultivation, and harvesting technology investments (Li & Yao, 2024; Ugwuanyi et al., 2024).

Another innovative development includes solar thermal desalination technologies that use the available solar thermal energy to treat brackish water or wastewater for reuse in integrated systems for cleaning animal facilities or irrigation. In turn, this supports the improved water-use efficiency while indirectly reducing emissions related to water procurement and pumping. These technologies will particularly suit livestock production systems compared to locations in dry and semi-arid regions where water scarcity limits animal production outputs (Aryanfar et al., 2024).

Waste-to-hydrothermal liquefaction (HTL) systems provide a highly efficient way to transform wet organic waste, like manure or slaughterhouse waste effluent, into biocrude oil under high pressure and moderate temperature. HTL can replicate natural geological processes of fossil formations on a cosmological time-scale, and it is especially well suited to high moisture feedstocks that are problematic for other thermochemical pathways. Hopefully, this emerging technology will provide opportunities for livestock farms to become net energy producers while decreasing methane and nitrous oxide emissions from unmanaged waste (Feng et al., 2024; Jagadale et al., 2024).

Dual-use agrivoltaic systems- installing solar panels above grazing land- are an additional opportunity that has co-benefits of energy production and land-use efficiency. Agrivoltaic systems with livestock reduce heat stress on animals, maintain the pasture quality of grazing land, and develop additional sources of revenue for the farmer. Agrivoltaics and livestock complement one another and could have application in regenerative agriculture and climate-smart agriculture, but require a good deal of planning to not interrupt animal movement or access to forage (Zheng et al., 2024).

When taken together, these emerging and hybrid renewable energy systems signify an actual shift in the energy-waste-emissions interface within livestock production. These systems are not yet in a phase where they can be implemented at the commercial scale we typically associate with livestock production, given the technical, financial, and regulatory barriers to deployment that we discussed in this i-statement. The technologies and processes being developed to support livestock production principles of resilience and self-sufficiency in energy support the research, collaboration across sectors, and policy environment for climate action. Future details of these systems integrations will likely be based on a series of designs to address specific production systems, environmental settings, and economic environments.

## 8 Circular economy models

Through resource cascading and closed-loop designs, modern circular economy models go beyond traditional waste management techniques to create regenerative systems that match industrial activity with ecological thresholds. In order to track substance flows both inside and outside of supply chains, these models increasingly rely on industrial symbiosis networks and material flow analysis platforms. This allows for high rates of material recirculation and promotes eco-industrial systems that integrate various resource streams. Modern circular systems use digital product passports with nano-tagged identifiers to enable intelligent, autonomous sorting at material recovery facilities, frequently aided by hyperspectral imaging techniques. They also integrate cradle-to-cradle design principles with digital technologies like blockchain to confirm the recycled content of materials (Perillo, 2024).

By employing solvolysis techniques that use supercritical fluids to recover fibers and chemical feedstocks appropriate for additive manufacturing, technological advancements enable the recovery and reuse of materials that were previously unrecyclable, such as carbon-fiber-reinforced plastics. By combining biomimetic membrane technologies with sophisticated desalination units driven by waste heat, water reuse systems are now achieving near-zero liquid discharge targets, exhibiting notable reductions in ecosystem impacts when compared to conventional linear production-consumption systems. The environmental performance of these integrated circular systems is increasingly being validated by international standards, such as ISO certifications (Yuan et al., 2024; Zhang, 2024).

Additionally, market mechanisms are changing from static extended producer responsibility schemes to dynamic deposit-refund systems driven by AI-driven sorting technologies. These systems modify incentives in real-time according to market conditions and resource scarcity data. Sustainable resource use is promoted by new business models like chemical leasing, which link profits to material performance rather than sales volume. By using bioleaching and sophisticated separation techniques to recover essential metals from electronic waste with purities higher than those of primary sources, urban mining initiatives have become important contributors to resource circularity (Kandpal et al., 2025).

With digital twins of circular systems running on federated learning platforms that compile data from millions of IoT devices across international manufacturing networks, digitalization is speeding up the shift to a circular economy. Digital product passports allow for real-time tracking of sustainability indicators, ensuring transparency throughout the lifecycle of materials and products, while advanced optimization algorithms, including quantum computing techniques, are applied to remanufacturing schedules to increase efficiency. Strong circular value chains are further supported by energy-efficient blockchain technologies, which enable safe transactions and sustainability parameter certification (Ababio et al., 2025).

Tax incentives encourage investments in modular, disassembly-ready equipment that prolongs product lifespans and aids in resource recovery, and frameworks such as the Circular Transition Indicators inform green finance instruments. Economic instruments are becoming more and more connected to circularity metrics. In order to improve equity in the shift to circular economies, social considerations are being incorporated through inclusive policies that formally include informal waste collectors in established collection networks (Ануфријев & Dašić, 2024; Mehrotra, 2024).

New developments include plasma gasification systems that turn non-recyclable residues into syngas for carbon-negative building materials and synthetic biology platforms that engineer microbial strains to upcycle mixed plastic waste into biopolymers. Circular economy models are redefining value creation and sustainability strategies, supporting a regenerative economic paradigm in line with planetary boundaries, as global standards for circularity reporting are improved and AI-driven material marketplaces enhance traceability and matchmaking between industrial byproducts and downstream applications (Osman et al., 2024). Circular economy models in livestock systems to mitigate GHG emissions and optimize resources are presented in Table 7.

**Table 7:** Circular economy models in livestock systems to mitigate GHG emissions and optimize resources.

Model	Primary inputs	Conversion process	Outputs/Byproducts	GHG mitigation mechanism	Key implementation challenges
Integrated livestock-crop	Manure, crop residues	Anaerobic digestion/composting	Biogas, organic fertilizer	Reduces CH₄ from manure; replaces synthetic N	Land-use coordination, nutrient balancing
Insect-based feed	Organic waste, agri-byproducts	Insect bioconversion (BSFL*)	Protein meal, frass fertilizer	Avoids deforestation for feed crops; 30–50% lower CO₂ vs soybean	Regulatory approvals, consumer acceptance
Algal bioremediation	CO₂, nutrient-rich wastewater	Photosynthetic biomass growth	Algal protein, biofertilizers	Captures 1.8–2.5 kg CO₂/kg biomass; reduces N₂O from wastewater	Energy-intensive harvesting, strain optimization
Agroforestry/Silvopasture	Lignocellulosic biomass	Integrated tree-livestock systems	Fodder, timber, carbon credits	Sequesters 3–8 ton CO₂/ha/yr; reduces enteric CH₄ via tannin-rich foliage	Long establishment period (5–15 yrs)
Grassland restoration	Degraded pastures	Rotational grazing management	Improved soil organic carbon	Increases SOC⁎⁎ by 0.5–1.2%/yr; reduces CH₄ through optimized forage	Requires adaptive herd management

⁎ BSFL = Black soldier fly larvae;

⁎⁎ SOC = Soil organic carbon

### 8.1 Integrated livestock-crop systems

In order to close nutrient loops and improve agroecosystem resilience, integrated livestock-crop systems have developed into complex bioregenerative models that carefully coordinate animal production with precise crop management. These systems optimize mineralization and ensure effective nutrient cycling that supports soil fertility while minimizing environmental losses by utilizing artificial intelligence and sensor-based technologies to dynamically match manure nutrient availability with crop demand. For example, automated vermifiltration systems are being used more and more to convert livestock slurry into nutrient-rich vermicompost and pathogen-reduced liquid biofertilizers. This helps to improve biochemical soil health parameters in rotational cropping systems and enhance soil organic matter (Kazembe & Mkandawire, 2024; Vlaicu et al., 2024).

Methane-inhibited anaerobic digestion technologies are being incorporated into dairy operations through the development of complex integrated configurations. By solid-state fermentation of fiber fractions, these systems capture substantial amounts of biogas potential while stabilizing slurry pH and maintaining nitrogen content for crop uptake. Such methods directly incorporate circular principles into farm management by combining the processes of manure treatment and renewable energy generation (Nagarajan et al., 2024).

In order to guide grazing patterns, maximize forage utilization, and more evenly distribute manure nutrients across fields, sensor-assisted rotational grazing strategies that use unmanned aerial vehicles and remote sensing have made it possible to precisely assess pasture biomass. In order to improve water quality and stop nutrient leaching, these systems usually incorporate phytoremediation buffers planted with hyperaccumulator crops. Insect meal raised on manure solids and algae grown in nutrient recovery systems are examples of on-farm-produced ingredients that have been incorporated into circular feed strategies, which have also evolved to reduce the carbon intensity of livestock diets and support feed autonomy at the farm level (Ogungbuyi et al., 2024; Pan et al., 2024).

Advanced subsurface drip irrigation systems that optimize nutrient delivery and eliminate volatilization losses by delivering treated digestate directly to crop root zones maximize the synergies of water resources. IoT-enabled sensors for real-time soil moisture monitoring guarantee that irrigation events are precisely timed to preserve soil water balance, increasing water-use efficiency and promoting sustainable crop production (Thote et al., 2024).

Integrated livestock-crop operations are now encouraged by supportive policy frameworks, such as those found in developing agricultural policies, through eco-scheme payments connected to thorough sustainability monitoring. These initiatives monitor a wide range of social and environmental metrics, such as the effects of biodiversity and nutrient use efficiency, and they promote data-driven strategies that link cropping and livestock systems into robust, integrated wholes (Bhati et al., 2024).

New developments in these integrated systems include vertical farming modules that use biogas production's nutrient-rich effluent streams to grow hydroponic fodder on-site while absorbing and using carbon dioxide emissions to boost plant growth. Localized feed and grain exchanges between farms are made possible by digital platforms and blockchain-enabled networks, which lower transportation-related emissions and separate the production of proteins from international commodity chains (Munim & Islam, 2024). These systems are also being reshaped by materials innovation, such as biodegradable housing components made from agricultural residues that support soil health at the end of life and robust silage films that are made to withstand degradation while preventing the formation of mycotoxin. By modifying farm infrastructure, such as turning barns into stormwater retention facilities during periods of intense rainfall, climate resilience strategies are further integrated, and water management is in line with the circular objective (Bhattarai & Janaswamy, 2024).

A paradigm shift in livestock-crop synergies is represented by these integrated systems taken together, making animal agriculture a key component of circular bio-economy strategies. They make it possible to simultaneously increase soil carbon storage, nutrient efficiency, and emission reductions, turning livestock production from a source of environmental harm into a key element of regenerative, climate-smart agriculture.

### 8.2 Insect-based feed production

By using highly effective bioconversion systems to convert organic waste streams into high-value protein sources, the industrial-scale production of insect-based feeds has become a key component of circular bioeconomy strategies. These systems transform a wide range of low-value organic substrates, such as agricultural residues and food industry byproducts, into nutrient-rich insect meals fit for animal nutrition by using species like mealworms and larvae of black soldier flies. Modern vertical insect farms use controlled-environment bioreactors, which carefully regulate variables like temperature, humidity, and aeration to maximize larval growth and metabolic efficiency. Productivity is further advanced by genetic innovations, such as strains that have been selectively bred or genetically edited for faster development and improved feed conversion (Nielsen et al., 2025; Odongo et al., 2024).

All components of insect biomass can be fully utilized thanks to integrated processing technologies. Insect production produces high-protein meals for animal feed, as well as valuable byproducts like chitin, which is converted into chitosan for use in water treatment and biodegradable materials, and frass, an organic fertilizer rich in nutrients. Developments in lipid extraction methods also make it easier to produce insect oils that are high in essential fatty acids, providing aquaculture feeds with sustainable substitutes for conventional marine-derived ingredients (Psarianos et al., 2024).

Innovations in processing, like the enzymatic hydrolysis of insect biomass, produce highly digestible protein fractions and bioactive peptides that have been shown to improve livestock and aquaculture growth performance. By significantly lowering the amount of land, water, and energy used, these methods help to reduce the environmental impact of feed production when compared to traditional protein sources (Fu et al., 2024).

In order to maximize substrate utilization and conversion efficiencies, feedstock composition can now be dynamically adjusted thanks to sophisticated sensor systems that track important larval biomarkers in real time. These monitoring systems are connected to cutting-edge control platforms that use AI to adjust environmental parameters, enabling reliable and expandable production (Huang & Khabusi, 2024).

The use of heat recovery techniques, which redirect the metabolic heat produced by larval growth to maintain temperatures in nearby bioreactors or greenhouse facilities, is an example of circularity in insect-based feed systems and improves overall energy efficiency. Integrated water management solutions also make it possible to recycle process water, which lowers the need for freshwater and lessens the environmental effect of operations (Valverde-Orozco et al., 2024).

Insect meal has recently been added to the list of approved feed ingredients for a variety of livestock species by regulatory frameworks in a number of regions, which has encouraged investments in large-scale production facilities and wider adoption. In addition to offering transparent records of feedstock sources, processing parameters, and quality control results, certifications and blockchain-based traceability systems guarantee adherence to safety and hygienic standards (Andhale, 2024).

Emerging applications extend beyond feed production, with insect-derived compounds increasingly explored for use as natural antimicrobial agents in silage preservation and as biodegradable chelating agents in organic hydroponics. Moreover, insect frass is increasingly recognized as an effective biofertilizer, contributing to soil health and reducing dependence on synthetic fertilizers.

By creating symbiotic microbial consortia to pre-digest lignocellulosic residues, innovative biofoundries are increasing the suitability of substrates for larval growth and boosting the effectiveness of nutrient cycling. Insect farming can be in line with climate mitigation objectives by combining insect frass with other carbon-rich amendments, like biochar, to create fertilizers that are carbon-negative (Ashworth et al., 2025).

As standards for insect-derived products continue to evolve internationally, insect-based feed production is positioned to expand through global supply chains, facilitating transcontinental exchanges of raw materials and processed insect proteins. This rapidly advancing sector exemplifies how circular economy principles can transform waste into a valuable resource, contributing to food security, climate resilience, and sustainable agricultural systems.

### 8.3 Algal bioremediation systems

As dynamic metabolizers that can convert wastewater streams into useful biomass while addressing environmental pollution, algae bioremediation systems have developed into cutting-edge ecological technologies. Modern systems now incorporate multi-stage photobioreactors made to treat a variety of wastewaters, from acid mine drainage to municipal and agricultural effluents, while concurrently recovering high-value compounds like pigments and lipids for use in feed or nutraceutical applications. These systems use extremophile and engineered strains of microalgae (Reehana et al., 2024).

Modern installations use creative reactor designs that maximize hydraulic conditions and improve light utilization, such as photobioreactor cascades and serpentine raceways. To increase photosynthetic efficiency, advanced materials like surfaces enhanced by nanoparticles are added. Meanwhile, semi-permeable membranes allow for the controlled release of carbon dioxide, which is frequently obtained from industrial flue gases and further encourages algal growth and carbon sequestration (Salim, 2024; Sumathi et al., 2024). These systems are based on circular strategies, whereby harvested algal biomass is processed using hydrothermal liquefaction to produce nutrient-rich biochar that can be used as slow-release fertilizers and renewable bio-oils that work with the refinery's current infrastructure. Algal systems contribute to regenerative agricultural practices by closing nutrient cycles and reducing the need for synthetic fertilizers through these pathways (Samoraj et al., 2024).

Sophisticated control frameworks now integrate real-time monitoring of water quality parameters, enabling dynamic adjustments of hydraulic retention times and algal community compositions. Machine learning models trained on comprehensive water datasets support these adaptive operations, allowing systems to respond to fluctuating pollutant loads and optimize contaminant removal and biomass productivity. These systems can now reach contaminated areas like industrial discharge streams and agricultural runoff zones thanks to the use of engineered algal strains that can bind and remove heavy metals. Algal-bacterial consortia are being investigated for simultaneous nitrification-denitrification, which goes beyond conventional phycoremediation and offers sophisticated solutions for comprehensive nitrogen management in a single integrated unit (Bhalerao & Mohan, 2024).

With industries like breweries and dairy providing organic-rich effluents to algal farms, where algae recover nutrients and purify the water, industrial symbiosis networks are becoming important facilitators of algal circularity. The resultant biomass creates resource loops that benefit both sectors by being used as a feedstock for aquaculture feeds, bioplastics, or bioenergy (Rengarajan et al., 2024).

Novel applications include coastal and marine settings, where harvested biomass can be used to replace petrochemical-based packaging materials with biodegradable ones, and algal turf scrubbers are placed beneath offshore structures to absorb excess nutrients. Algal systems also show promise in desalination technologies, utilizing algal cultivation in conjunction with forward osmosis to recover freshwater from salty sources and generate biomass rich in lipids for renewable energy (Kaleeckal et al., 2025).

Algal systems' benefits to the environment and climate are becoming more widely acknowledged by policy and certification frameworks, which encourage their incorporation into regulatory regimes through mechanisms like water stewardship certifications and verified carbon sequestration credits. Blockchain-based platforms for reporting, monitoring, and verification also guarantee the transparency and traceability of sustainability results (Yadav et al., 2024).

In the future, studies on synthetic biology and bacterial co-culture systems seek to improve algal performance so that biopolymers, bioactive compounds, and wastewater treatment processes can all be produced at the same time. New photobioreactor designs continue to increase scalability, from decentralized village-level installations to industrial-scale operations, while sophisticated control algorithms utilizing artificial intelligence speed up the identification of the best algal strains for particular remediation challenges (Nayak et al., 2024).

As these systems develop, they offer a potent means of turning liquid waste streams into useful resources. Algal technologies are integrated into circular economy frameworks, which meet the demands of sustainable food production, industrial symbiosis, and environmental stewardship.

### 8.4 Agroforestry and silvopasture practices

Trees, pasture species, and livestock are all combined into ecologically beneficial arrangements in agroforestry and silvopasture systems, which are integrated, multipurpose land-use strategies. By increasing carbon sequestration, reducing greenhouse gas emissions, and boosting climate resilience, these systems are intended to maximize ecosystem services. Agroforestry systems, which support long-term soil carbon stabilization and mitigate climate change, achieve higher rates of photosynthetic carbon fixation than traditional monoculture pastures thanks to layered vegetation structures that include tree canopies, shrubs, and herbaceous layers (VijayKumar et al., 2024; Worku, 2024).

Improving methane mitigation pathways in ruminant digestion is one of the main functions of silvopastoral systems. By strategically adding tannin-rich tree foliage, methanogenic archaea populations are decreased, and propionate-producing bacteria are increased in the rumen microbial communities. Enteric methane emissions are reduced by this biochemical change, which restricts the amount of hydrogen available for methane production. In order to promote nutrient cycling and stabilize organic matter in the rhizosphere through extensive mycorrhizal networks, deep-rooted tree species work in tandem with herbaceous plants to redistribute nutrients within the soil profile (Khejornsart et al., 2024).

Agroforestry and silvopasture management have been revolutionized by technological advancements. Real-time mapping of root zone carbon dynamics and nutrient flows is now possible thanks to precision agriculture tools like distributed fiber optic sensing and hyperspectral imaging. In order to optimize productivity and ecological advantages, adaptive management of tree-livestock arrangements is supported by this degree of spatial-temporal resolution (Bonannella, 2024).

Woody biomass from tree pruning can now be converted into biochar, which serves as a rumen additive and soil amendment thanks to advancements in pyrolysis technology. By improving soil water retention, increasing soil fertility, and lowering enteric methane emissions, biochar establishes self-contained material loops that support the ideas of the circular economy. Additionally, by strategically placing tree belts, microclimate engineering lowers maintenance energy requirements and improves animal welfare and feed conversion efficiency by reducing heat stress in grazing livestock (Supraja et al., 2023).

Agroforestry systems establish interdependent material cycles that incorporate hydrological cycles, where improved evapotranspiration and water infiltration increase drought resistance and water retention; nutrient cycles, where nitrogen-fixing tree species restore soil fertility and lessen dependency on synthetic fertilizers; and biomass cascades, which convert woody residues into fodder, energy, and soil enhancers. New financial tools are being developed to help put these systems into place, such as carbon markets with real-time blockchain verification that integrate subsurface carbon accounting with satellite-derived biomass measurements and neutron probes. The temporal aspect of carbon sequestration is being acknowledged by policy frameworks in progressive regions, which are introducing tools like agroforestry bonds that pre-finance future benefits of carbon storage (Balasubramanian et al., 2024; Jhariya et al., 2024).

Additionally, breeding programs are progressing, creating new tree cultivars using contemporary biotechnologies, such as gene editing, to hasten canopy establishment and reach carbon neutrality earlier than with conventional varieties. By connecting dynamic stocking density models with canopy cover data, augmented reality platforms are improving livestock management by allowing for precise grazing intensity adjustments (Cao et al., 2024b). Beyond carbon, silvopastoral systems promote greater biodiversity, such as improved functional bird diversity, and natural pest control, which lessens dependency on chemical inputs. By increasing water infiltration and decreasing surface runoff, these systems also enhance landscape hydrology and help prevent flooding (VijayKumar et al., 2024).

Agroforestry and silvopasture systems have a lot of potential to improve the sustainability of livestock production by sequestering carbon, conserving biodiversity, and controlling microclimate. The acceptance and success of such practices, however, are very context-specific based on the size of the farm, land tenure systems, availability of labor, and climatic conditions of the area. Multifunctional land use can be helpful in smallholder and pastoral systems, but these systems may be constrained by a lack of legal ownership of land or access to planting material, whereas large operations may have difficulties with the logistical and managerial aspects of incorporating trees into grazing systems. Climate variability also influences the results, as it affects the activity of trees that survive, their growth, and their relationships with forage plants.

Agroforestry and silvopasture systems deliver benefits that usually pay off in the medium- and long-term periods, and substantial carbon sequestration and ecosystem service benefits tend to accumulate in several years to decades. The establishment of trees, growth of canopy, and building of carbon in the soil is a slow process that requires long-term planning and management commitment. This timescale highlights the significance of linking agroforestry interventions to long-term mitigation goals as opposed to short-term emission reduction goals and of making feasible expectations on the rate of environmental gains (Jhariya et al., 2024; VijayKumar et al., 2024).

An important determinant is economic feasibility. Initial expenses such as the cost of planting material, fencing, and labor, as well as the maintenance costs that are required on the farm, may be high investments. Agroforestry systems can be associated with trade-offs in certain cases, with forage availability or crop production, especially at the initial stages of establishment when resources are most competitive. However, there are, on the other hand, benefits that are long-term (diversified streams of income, less heat stress, better land productivity, etc.) that can counterbalance initial costs. It is essential to assess these trade-offs so as to make sure that agroforestry practices are economically viable in various production systems.

Agroforestry and silvopasture systems will be able to affect the productivity and welfare of livestock by providing better thermal comfort, protection against adverse weather, and supporting better grazing conditions. Reduced heat stress can have a positive impact on milk production, growth performance, breeding efficiency, and behavior, whereas alterations in forage composition and supply can affect meat and milk composition. These are the effects at the production level that are at the center of determining the overall sustainability of agroforestry systems, because mitigation strategies should favor product quality and farm profitability without detracting from them.

Intense dependence on the selection of tree species, the density of planting, and the management system determines the performance of agroforestry systems. A variety of species are able to have different growth rates, depths of roots, and nutrient requirements, affecting their competition with pasture species. Ineffectively developed systems can contribute to competition for light, water, and nutrients, and lower forage productivity and efficiency of the system. It is thus essential to compare the planting configurations and management strategies to establish the best practices that can balance the activities in carbon sequestration, biodiversity improvement, and livestock productivity.

Innovations allow the optimization of agroforestry and silvopasture systems. Precision planting strategies, multi-species planting, and integrated monitoring tools can help to use resources in a more efficient way and make management adaptive. Monitoring of tree growth, pasture condition, and animal behavior may be assisted by using digital technologies and remote sensing, which would help in making data-driven decisions. The traditional limitations can be overcome by having such innovations to enhance the resilience of the system in changing environmental conditions (VijayKumar et al., 2024).

Agroforestry and silvopasture should not be applied as a stand-alone intervention, but they should be used as part of a comprehensive mitigation measure. Climate benefits can be increased with the help of synergies with dietary management, genetic improvement, manure management, and renewable energy integration, and overall system efficiency will increase. The agroforestry designs should be region-specific, with various ecological and socio-economic conditions influencing adaptation, so that the impact and generalizability are maximized. The contextualization of these practices takes into consideration holistic changes of the livestock systems that contribute to enhancing climate vulnerability and multi-function agricultural landscapes (Goracci & Camilli, 2024; VijayKumar et al., 2024).

Future research avenues include the creation of self-assembling biochar-based nanocomposites that can alter soil pH and structure to maximize plant nutrition, as well as the use of quantum computing to model intricate plant-animal-soil interactions at previously unheard-of resolution. Collectively, these developments highlight the agroforestry and silvopasture systems' scalability and transformative potential as essential elements of sustainable, climate-resilient agricultural landscapes (Alagendran et al., 2024).

### 8.5 Grassland restoration and rotational grazing

Rotational grazing and grassland restoration are key tactics in circular livestock systems, providing coordinated avenues for improving nutrient cycling, carbon sequestration, and greenhouse gas emission reductions. These systems create dynamic plant-soil-animal interactions through the thoughtful arrangement of grazed landscapes. These interactions function as self-regulating ecological processes that maximize photosynthetic efficiency while reducing methane release. Advanced biotechnologies, like precision-bred grass-endophyte symbioses, are being used more and more in restorative grazing techniques to increase root exudation and promote the development of stable soil aggregates. By increasing the synthesis of soil-binding substances like glomalin, these aggregates enhance the resilience and carbon storage of the soil (Jordon et al., 2024; Rousset et al., 2024).

With the help of digital tools like IoT-enabled collars that offer real-time biomass mapping, livestock are now used as ecological engineering agents in precision rotational grazing systems. In order to maximize forage utilization and reduce enteric methane intensity, machine learning algorithms can then match grazing schedules with the best vegetative regrowth stages. By using creative feeding systems that reroute fermentation pathways from methane production to more effective absorption of volatile fatty acids, emerging bioelectrochemical grazing techniques further modify the rumen microbiota, thereby lowering emissions and improving ruminant energy conversion efficiency (Akintuyi, 2024; Vlaicu et al., 2024). These systems are completed by circular nutrient management techniques, which turn manure into biofertilizers with nitrification inhibitors to increase the efficiency of nitrogen use and reduce nutrient losses. Deep-rooted perennial species in restored grasslands serve as multipurpose landscapes that improve fertility, stabilize erosion-prone soils, and lessen dependency on artificial inputs. Blockchain-based carbon credit platforms are being used more and more to validate soil carbon gains, providing farmers with new financial incentives. In order to monitor soil carbon with high spatial resolution and guarantee transparency and traceability in carbon accounting, these platforms incorporate technologies like distributed ground-penetrating radar scans (Davis et al., 2024).

Another innovation is the use of microbial fuel cells in grazing corridors, which use the oxidation of root exudate to produce electricity for electric fencing systems while simultaneously gathering data on the condition of the soil in real time. Methane oxidation biofilters, which use methanotrophic bacteria on biochar substrates, passively capture leftover methane emissions along the edges of rotationally grazed pastures, further reducing greenhouse gas emissions without the need for external energy inputs (Rani et al., 2024).

With deep-rooted polycultures that allow for sustained forage productivity under climatic stresses like protracted droughts, restored grasslands created under these systems demonstrate improved ecological resilience. Through hydraulic lift mechanisms, these systems enable improved soil hydrology, significantly raising infiltration rates and lowering the risk of flooding by improving the soil's ability to absorb water. These practices are supported by additional technological advancements. For example, gene editing in forage species has prolonged grazing periods by postponing plant senescence, and quantum-tagged biofertilizers allow precise nutrient tracking through hyperspectral imaging. Non-toxic ligninolytic fungi and advances in co-cultivated mycorrhizal inoculants speed up the breakdown of fibrous plant matter, lowering the production of methane precursors and improving forage digestibility (Ning et al., 2024; Saidova et al., 2024).

Because integrated manure processing centers use anaerobic digestion in conjunction with cutting-edge nitrogen recovery technologies to turn livestock waste into renewable biogas while also removing emissions of reactive nitrogen compounds, circularity in these systems extends to the production of energy. Rotational grazing systems are made more sustainable and economically viable by these facilities, which generate net energy gains that can balance farm energy requirements (Bumharter, 2024; Nagarajan et al., 2024).

In the future, integrating these components using digital twin simulations has the potential to be revolutionary. Managers can evaluate trade-offs between productivity, greenhouse gas mitigation, and ecosystem health by modeling the effects of various practices over decades by building virtual versions of grazing ecosystems. It is anticipated that these tools will direct strategic choices that maintain livestock systems' productivity while guaranteeing that agricultural methods stay in line with the climate and the limits of the planet (Arulmozhi et al., 2024).

## 9 Environmental impact assessment tools

Tools for environmental impact assessment have come to be an indispensable tool in future livestock systems to monitor GHG emissions and support evidence-based decision-making. These emissions grow with the increasing livestock production as we continue to feed the growing global demand for animal protein with a growing supply of animals, with a greater animal protein footprint on GHG emissions, land degradation, water use, and nutrient pollution, etc. This footprint has been spotlighted and is under intense scrutiny. The scientific community, policy makers, producers, and supply chain participants are looking towards thorough, standardized evaluation processes to examine, evaluate, report, and improve the environmental performance of livestock systems. These environmental assessment tools are designed to employ a systematic identification and measurement of the environmental impacts of livestock systems through the entirety of production from feed establishment, animal husbandry, manure management, and processing and distribution. These tools are beneficial in providing a broader understanding of the source and level of emissions and analyses in order to develop targeted mitigation strategies that are technically feasible and economically viable (Nugrahaeningtyas et al., 2024; Symeon et al., 2025).

These tools are fundamentally about achieving transparency and consistency in impact assessments while enabling product comparisons related to different production systems, regions, species, and management practices. This is especially important to consider, as the diversity of livestock production systems can range from intensive, high-input production systems that seek to maximize animal performance to extensive, pasture-based systems in low-income countries. Furthermore, environmental impact assessment tools can help to develop consistency in measuring sustainability, as countries and industries can show alignment with international climate commitments, such as the Paris Agreement and national GHG inventories. These tools will also serve as a platform for users of various sustainability-related corporate reports, product eco-labels, and supply chain assessments that are being driven by the increased understanding of the environmental impact of livestock-derived products from consumers and retailers (Recktenwald & Ehrhardt, 2024; Tipan-Torres, 2024).

Improvements in data analytics, remote sensing, and digital agriculture have gained additional traction by increasing the precision and scalability of these assessment tools to assess emissions and resource use on farms. Domestic and global organizations increasingly support developing real-time and near-real-time assessments of emissions and resource use. As life cycle inventories, emissions factors, geography, location, and production parameters converge within the tool, the tools adopt a systems-based approach to evaluating sustainability. Moreover, the tools allow for scenario modeling so that stakeholders can estimate the potential impacts of numerous mitigating strategies (e.g., altering diets, manure treatment technologies, or genetics) on the overall carbon footprint, resource use efficiency, and other environmental factors of livestock systems. The predictive attributes of the tools are an essential component of targeting investment, formulating policy, and prioritizing research (Romantseva & Bodur, 2024; Sirilakshmi et al., 2024).

In recent years, the emergence and extension of regionally relevant, needs-based tools have made environmental impact assessments more accessible to farmers and advisors, around which bridges the research and practice. When integrated as part of decision support platforms, these tools provide specific advice to producers on how they can reduce emissions without negatively impacting productivity or profitability. However, there are still challenges regarding data availability, methodological harmonization, uncertainty, and the potential trade-offs across environmental, economic, and social aspects of sustainability. As the livestock sector adapts to climate change and resource scarcity, environmental impact assessment tools will continue to provide essential pathways towards transitioning towards climate-smart, resilient, and low-emission livestock production systems (Iakovidis et al., 2024). Environmental impact assessment tools for livestock GHG management are shown in Table 8.

**Table 8:** Environmental impact assessment tools for livestock GHG management.

Tool category	Representative tools	Spatial resolution	Temporal scope	Key GHG metrics	Data requirements	Strengths	Limitations	Integration potential
LCA frameworks	FAO GLEAM, IPCC Tier 2/3, AgBalance®	Farm to global	Cradle-to-grave	CO₂eq (CH₄, N₂O, CO₂), land use change	Life cycle inventory databases	Holistic system boundaries	High data intensity (300–500 parameters)	Links to economic models
Carbon calculators	COMET-Farm, Cool Farm Tool, CAP'2ER®	Field/farm level	Annual cycles	Enteric CH₄, manure N₂O, feed emissions	Farm management records	User-friendly interfaces	Narrow scope (excludes upstream inputs)	Real-time decision support
Benchmarking databases	AgFoot, CLIMAT-FOOD, Livestock EIA Database	Regional/global	Decadal trends	Emission factors, intensity (kg CO₂eq/kg product)	Peer-reviewed studies	Cross-system comparability	Update latency (2–5 yrs)	Policy scenario modeling

### 9.1 Life cycle assessment (LCA) frameworks

LCA methodologies are among the most rigorous and scientifically credible approaches to measuring social responsibility in livestock product systems from the farm gate through the entire value chain. Assessing livestock production uses a cradle-to-grave or cradle-to-gate framework, systematically evaluating the emissions and resource use from feed production inputs, animal rearing, and manure management aspects of the production cycle, to downstream functions including processing, transport, retail, as well as loss and disposal through consumption. The value of the systems approach is that it will enable a better understanding of the total environmental liabilities of livestock systems, where solutions could decidedly have trade-offs, help with emission hotspots, and being able to develop goals towards mitigation strategies. In the context of greenhouse gas emissions, an LCA considers within the livestock production systems space, carbon dioxide (CO₂), methane (CH₄), and nitrous oxide (N₂O), converting the emissions into carbon dioxide equivalents (CO₂e), based on global warming potential (GWP) values measured over 20-year or 100-year periods (Horrillo et al., 2024; Recktenwald & Ehrhardt, 2024).

The strength of LCA is derived from its standardized methodological framework, with the ISO 14040 and 14044 standards mostly dictating the phases of LCA: goal and scope definition; life cycle inventory (LCI); life cycle impact assessment (LCIA); and interpretation. In livestock systems, the goal and scope usually define the system boundaries (e.g., farm gate, processor gate, or consumption of the product), the functional unit (e.g., per kg of liveweight, milk, meat, or protein), and how to allocate emission burdens on co-products like meat, milk, wool, or manure. The inventory stage is the most data-laden. Data must be assembled to describe the resources used for livestock production, like the type and origin of feeds, energy consumed (electricity and fuel), water consumed, the performance of the animal, how manure is stored, processes for land-use change, etc. These inputs would then parlay into existing databases and emission models from the agricultural sector (i.e., IPCC Tier 2 or Tier 3 methodologies to assess direct and indirect emissions at a greater resolution and accuracy than emission inventory methods) (Aviso, 2024; Kang et al., 2024).

Life Cycle Impact Assessment utilizes characterization factors to translate inventory data into environmental impacts. The most commonly used characterization factor for GHG is the Global Warming Potential (GWP), although it is possible to include other environmental categories (e.g., acidification, eutrophication, land use, water use) for added perspective on environmental sustainability. The interpretation of LCA results allows for scenario modeling to test how changes in management practices (e.g., change feed composition; use of methane inhibitors; amended manure treatment; improved animal productivity) can affect the GHG emissions profile. The interpretation of results is an important step to provide actionable recommendations for management changes to better align production systems with climate mitigation goals (Noble et al., 2024; Xue et al., 2024).

While LCAs have significant positives when applied to livestock systems, they can still encounter challenges. Data availability and especially data quality can be substantial factors in some regions, particularly in low-to-medium income countries, where record-keeping may not be standard practice. Variability in facts and figures from varying production systems, agro-ecological conditions, and assumptions and methods can lead to inconsistency in results and limit the comparability of LCAs across studies or regions. Allocation of emissions among co-products is also problematic in LCAs, emphasizing how a decision in allocation, either on a mass basis, economic basis, or through system expansion, will influence and change the overall results. With these shortcomings in mind, efforts are occurring globally to bring similar methodologies around LCAs for livestock, including the FAO's Livestock Environmental Assessment and Performance (LEAP) Partnership, which provides guidance and emission factors for livestock-sector specific LCAs (Cruz-Martinez et al., 2024).

In addition, LCA frameworks are finding their way into digital platforms and decision support tools that allow farmers, processors, and policymakers to complete a customized assessment without the need to have a sophisticated technical understanding. Usually, these opportunities are available with user-friendly templates and cloud-based databases, to be able to complete quasi-real-time scenario assessments and benchmarking. LCA is also becoming a dominant factor in sustainability certifications, product labeling, and environmental product declarations (EPDs), which have a direct role in market access and consumer acceptance. As climate change and sustainability are increasingly considered in how the livestock sector is governed, LCA will also be a valuable analytical foundation to identify significant mitigation opportunities, the basis to direct research investments, and develop environmentally responsible supply chains (Ramakrishna & Brindha, 2024).

### 9.2 Livestock carbon footprint calculators

Livestock carbon footprint calculators are handy tools to quantify the GHG emissions associated with many of the components of livestock production using a calculator format that is easily understandable and accessible. These calculators are simplified, often farm-specific implementations of advanced methodologies for environmental assessment, such as LCA, turning scientific emission factors and modeling concepts into simpler formats that end-user farmers, extension agents, researchers, and members of the supply chains can work with. The main purpose of these tools is to chart the emissions, mainly CH₄, N₂O, and carbon dioxide, into several emitting sources (enteric fermentation, manure handling, and land uses or feed production related fuel and energy use), often reported as CO₂e. They play a key role in enabling producers be able to measure their environmental footprint, identify emission hotspots, and evaluate cost-effective and context-specific mitigation opportunities (Pelton et al., 2024; Recktenwald & Ehrhardt, 2024).

They are usually built with empirical datasets, Tier 1 to Tier 3 emission factors from the IPCC, and/or livestock species, feed intake levels (regions), systems, and production systems, whereas real-world variability exists. Using basic farm-level data readily available, animal numbers, ADG, feed intake, waste management practices, manure handling, grazing strategies, fertilizer use, and energy consumption, producers can use the framework to model a comprehensive emissions profile per component of their farm. Most calculators also incorporate settings by management intervention, and therefore, users are able to model the implementation and adoption of mitigation practices like feed additives, new genetics, anaerobic digesters, or desirable grazing systems. It is useful when communicating with producers on climate-smart livestock management and highlighting a less reactive approach to emissions reduction (Nugrahaeningtyas et al., 2024).

The convenience of carbon footprint calculators is what makes them so effective and beneficial. As opposed to full LCA models (often involving expertise as well as inaccessible datasets), these tools are aimed at quick assessments and can provide real-time feedback with a low level of technical training. They are mostly built within web-based tools and mobile applications with dashboards that enable a connected interaction and visual outputs, hence well-suited for on-farm decision-making and extension services. Some calculators are also linked to carbon accounting or certification architectures, which means producers can create reports that comply with carbon offset programs, environmental labelling, and so forth, according to guidelines on the way. At some stage, for example, some farm assurance schemes now include carbon calculators or are linked to incentives that reward emissions reductions via market-based mechanisms and, secondly government subsidies (Michla et al., 2024).

A variety of national and international livestock carbon calculators have been developed to fit particular production contexts. Well-known examples include Cool Farm Tool, the Teagasc Carbon Audit Ireland, U.S. Dairy Greenhouse Gas Model (DairyGHG), and Agri-Footprint (applicable across the EU). They vary in scope and sophistication, some of which are single-species (e.g., dairy cows) while others will quantify multi-species assessments of cattle/sheep/pigs/and/or poultry. Other calculators are starting to include the co-benefits of mitigation strategies, e.g., improved productivity or nutrient use efficiency, to broaden the spectrum of sustainable trade-offs. Nevertheless, carbon calculators are far from perfect. When models are too simplified they might not fully represent the variability and complexity of real production systems or results can be influenced by how well data distance the user entered information for example while underlying assumptions are correct and they may be insensitive to spatial and temporal factors like soil type, climate seasonality or feed availability with seasonal performance differences in animals (Díaz de Otálora et al., 2024; Sikiru et al., 2024).

With climate policies and market-based incentives leading towards quantifiable emission reductions from the livestock sector, carbon footprint calculators will serve as invaluable transparency, accountability, and strategic planning tools. Their adoption in farm-level farm management software, carbon trading platforms, and environmental reporting systems is one such promising pathway to arrive at the mainstreaming of climate action within the livestock value chain (Sirilakshmi et al., 2024).

### 9.3 Benchmarking databases

Databases for benchmarking represent a powerful approach to quantify, benchmark, and hopefully improve the environmental performance of livestock systems. The Global Livestock Environmental Assessment Model (GLEAM), developed by the Food and Agriculture Organization (FAO), is a standard reference in livestock GHG emissions modelling per species, production systems, and geographical world regions. GLEAM uses detailed geospatial datasets and biological/ agronomic parameters to derive emissions as well as emission intensities, resulting in valuable information for recognizing environmental hot spots and setting targeted mitigation strategies. The life-cycle approach of not only direct emissions (methane produced from enteric fermentation) and stuff emitted linked with feed and feed production, land-use change, and energy consumption. It provides a tractable way to test various interventions across their potential impact on stakeholders (policy makers to agribusiness leaders, etc) (Bashar et al., 2024; Samad et al., 2025).

Although GLEAM is extensively used for international assessments and policy frameworks, other benchmark databases enhance these capabilities with regionally distinct, sector-specific, or application-driven insights. An example of an international platform is Agri Benchmark, a coordinated network by the Thünen Institute in Germany that offers benchmarking data on livestock and crop production systems across countries. While Agri Benchmark serves predominantly economic benchmarking, it integrates an increasing number of environmental indicators, enabling a two-weighted perspective for enterprises in the livestock sector to determine their profit and loss and sustainability. The dual orientation promises increased pragmatic decision-making, particularly on how best to reconcile mitigation strategies with economic viability (Chibanda et al., 2024; Verhaagh, 2024).

A significant other tool is the Cool Farm Tool, produced by the Cool Farm Alliance, a partnership of major agribusiness, food companies, and stakeholders. Cool Farm Tool (CFT) is a farm-scale decision support tool, and it estimates GHG emissions, water use, and biodiversity impacts. However, it is especially useful to producers and agrifood companies that wish to act on making their supply chains low-carbon. Cool Farm Tool, as opposed to GLEAM, which has been conventionally adopted for national or even smaller scale assessments, focuses on quick returns on farms in the form of actionable results readily based on real-time data input allowed producers to assess various management options (changes in feed, manure handling approaches, or grazing practices). It is generally well-suited to commercial supply chains, especially in dairying and beef sectors, with its easy-to-use interface and private-sector sustainability goals cohesion (Arivukkumar et al., 2024).

Likewise, carbon audit tools created in countries such as Ireland (e.g., Teagasc) and the UK (e.g., SAC Carbon Calculator by SRUC) offer farming condition-specific benchmarking on a regional level. These are frequently embedded in national climate strategies that enable producers to calibrate their GHG emissions reduction with increasing production efficiency. For example, the carbon footprinting service at Teagasc supports Irish livestock farmers in determining mitigation options appropriate for cost relevant to national commitments on the part of the country. This category of tools frequently taps into local databases and country-specific emission factors to improve the accuracy of assessment over global models (Watson et al., 2024).

Also, the Global Feed LCA Institute (GFLI) created a database to benchmark and evaluate the environmental implications of feed raw material. As feed production can make up a large proportion of overall emissions in livestock systems, with especially mono-stomach animals (pigs and poultry) having high feed-related emissions, feed companies and integrators must have feed-specific databases. Supply chain actors are now able to make data-driven feed and formulation decisions that drive towards sustainability goals thanks to high-level-of-detail sourcing data that warps GFLI's methodology in a harmonized and transparent manner, enabling comparisons across feed types, sourcing regions (Martin et al., 2017). Additionally, the harmonized guidelines and databases put forward by the LEAP Partnership, which is also hosted by the FAO, will boost the interoperability of multiple benchmarking tools. It encourages the common methodologies for environmental assessment of livestock systems, helping countries and value chains compare their results. The most important thing for carbon labeling schemes, sustainability certification programs, and national GHG inventories lies in consistency (Boulay et al., 2021).

New developments have led to the development of a platform that pulls farm-level performance data and provides benchmarking dashboards in real time using big data, too. These encompass digital livestock platforms utilizing a combination of satellite monitoring and IoT sensors that leverage machine learning based models to benchmark environmental metrics alongside animal welfare, health, and productivity. Combining emissions benchmarking with automated milking systems data, precise feeding technologies, and manure treatment sensors opens up these next-generation platforms and transforms how sustainability is monitored and managed over livestock farms (Vlaicu et al., 2024).

Though benchmarking databases are used quite widely, they have several limitations, data gaps, regional variability, and methodological inconsistencies, among others. Standardization efforts, through LEAP and LEAP-Standards, with the ISO standards being an example of this, are key in overcoming these hurdles. Moreover, advancing greater levels of transparency, outreach to broaden user training, and fostering accessibility (particularly for smallholder and resource-limited systems) are the highest priorities to increase adoption and impact (Jeon et al., 2024).

The Benchmark databases, such as GLEAM, AgriBenchmark, Cool Farm Tool, GFLI, and regional carbon calculators, are an emerging ecosystem of instruments that work together to improve livestock emissions measurement, reporting, and mitigation. An integrated set of relevant but complementary scales and scopes, a global assessment to a farm-level decision support framework, is appropriate to guide the transition of climate-resilient, resource-efficient, and economically sustainable livestock production systems. Through advances in new technologies, high-resolution data, and sectoral collaboration, as tools listen to these shifts, so will be essential in both the design and the implementation of mitigation strategies that allow for environmental and socio-economic objectives.

## 10 Policy, socioeconomic, and adoption considerations

The shift towards clean technology systems and circular bioeconomies necessitates a fundamental realignment of market structures, financial instruments, and regulatory frameworks. This evolution is intrinsically complicated because recent examples show how supply chains are changing and how the valuation of material flows is being redefined by carbon pricing mechanisms and new bioasset securitization techniques. New regulatory tools, like updated extended producer responsibility standards requiring significant amounts of post-consumer recycled content, and cutting-edge economic tools, like nutrient trading platforms and biodiversity impact bonds, which offer fresh incentives for ecological restoration, are having an increasing impact on policy landscapes at the local, national, and international levels (Chenavaz & Dimitrov, 2024; Zysman et al., 2024). Additionally, urban planning is changing as cities use blockchain-based material passports to track embedded carbon across several lifecycle stages and integrate industrial symbiosis zones close to organic waste sources. City-level policies can greatly advance material circularity goals, as demonstrated by regulatory approaches that permit adaptive reuse of construction materials (Munonye & Ajonye, 2024).

At the same time, sustainable finance tools are quickly growing outside of conventional frameworks. New risk management tools, such as catastrophe-linked derivatives, are being developed to hedge against potential disruptions in circular systems, and the financial sector is witnessing the rise of biomass-backed securities, where future outputs from bio-based industries like insect farming or algal cultivation can serve as collateral. Producers in areas that prioritize regenerative agriculture stand to gain significantly from the growing recognition of agricultural carbon capture techniques, such as soil-based biochar, as valid tradable offsets within global carbon accounting mechanisms by international monetary policy (Swinfield et al., 2024).

Using cutting-edge instruments that assess exposure to material inefficiencies, central banks and financial regulators are implementing forward-looking evaluations of portfolio resilience through circularity stress-testing. These actions encourage closed-loop production models and internalize the full economic costs of linear material use. They also support radical transparency initiatives (Roy, 2024).

At the same time, new approaches to governance are being developed, with AI-powered policy sandboxes enabling the simulation of circular economy tactics across a wide range of socioeconomic metrics. Large-scale citizen panels serve as an example of participatory foresight mechanisms, which are increasingly essential to inclusive and democratic regulatory processes, especially in fields where cutting-edge technologies like synthetic biology or nanomaterials interact with public and environmental health concerns (Ahern, 2025).

Development finance organizations are looking into pay-for-success agreements that incentivize local governments to invest in circular system-supporting infrastructure, like anaerobic digesters that produce co-benefits like lower medical expenses due to better waste management. Institutional investors are also starting funds specifically for the circular economy, which incorporate sophisticated material flow analysis into investment strategies and directly tie capital allocation to the potential for resource efficiency and industrial symbiosis. A systemic shift toward frameworks that value each avoided unit of waste as an economic asset, acknowledge regulations as crucial market indicators, and support governance structures that facilitate regenerative resource cycles is signaled by these developing policy, economic, and governance innovations taken together. It is anticipated that as these innovations advance, they will spark significant shifts in the ways that societies manage, produce, and consume materials, establishing the groundwork for robust, just, and sustainable economic systems (Huang, 2024; Komyshev et al., 2024). Policy, socioeconomic, and adoption considerations for GHG mitigation in livestock systems are presented in Table 9.

**Table 9:** Policy, socioeconomic, and adoption considerations for GHG mitigation in livestock systems.

Consideration category	Description	Role in GHG mitigation	Key stakeholders involved	Implementation barriers	Examples/Programs/Policies	Suggested interventions
Carbon pricing mechanisms	Market-based policies that put a price on GHG emissions (e.g., carbon tax, cap-and-trade).	Encourages low-emission practices and internalizes environmental costs.	Governments, industry, and producers	Political resistance, price volatility, and fairness concerns	EU Emissions Trading System; Canada’s carbon pricing model	Tiered tax exemptions, reinvestment in green tech
Certification and labeling	Standards for low-carbon livestock products (e.g., eco-labels, carbon-neutral meat).	Incentivizes consumers and producers to support sustainable practices.	Retailers, producers, and NGOs	Consumer awareness, standard harmonization, and cost of certification	USDA Climate-Smart Commodities Initiative	Subsidized certification, public campaigns
Financial incentives/Subsidies	Grants, low-interest loans, and tax breaks for GHG-reducing innovations.	Offsets costs for producers adopting sustainable tech.	Ministries of agriculture, banks, and cooperatives	Funding continuity, awareness, and application complexity	EQIP (USDA); Green Climate Fund	Streamlined application, bundling with insurance
Education and capacity building	Training, extension services, and knowledge-sharing platforms.	Empowers farmers and advisors to adopt and manage mitigation technologies.	Extension agents, universities, and NGOs	Low literacy, access in remote areas, and language barriers	FAO Farmer Field Schools; Climate Smart Agriculture programs	Tailored training modules, ICT-based delivery
Behavioral change strategies	Interventions targeting social norms, perceptions, and risk attitudes.	Addresses non-economic adoption barriers and enhances long-term sustainability	Psychologists, extension services, and local influencers	Cultural norms, resistance to change, and misinformation	Social Marketing Campaigns; Nudge Theory Applications	Peer-to-peer learning, behavioral nudges
Equity and inclusivity	Ensuring smallholders, women, and marginalized groups benefit from mitigation opportunities.	Promotes fairness and broad participation in mitigation efforts.	NGOs, local governments, and advocacy groups	Access inequality, gender bias, and land tenure issues	Gender in Climate-Smart Agriculture (CGIAR); GCF Inclusive Projects	Gender mainstreaming, community participatory planning
Regulatory mandates	Legal requirements for manure management, feed practices, or emissions reporting.	Sets minimum sustainability standards and enforces compliance.	Governments and inspection bodies	Enforcement capacity, industry lobbying, and non-compliance costs	EU Nitrates Directive; US Clean Water Act	Flexible timelines, tiered penalties
Market access and trade incentives	Trade preferences and premium pricing for sustainable livestock products.	Rewards environmentally friendly practices through enhanced marketability.	Exporters, trade ministries, and buyers	Certification complexity, trade protectionism, and pricing pressure	Fair Trade Livestock; WTO Green Box Support	Bilateral agreements, green export branding
Public-private partnerships (PPPs)	Collaborations for R&D, scaling technologies, and outreach.	Facilitates resource pooling and shared risks for innovative mitigation.	Companies, researchers, and governments	IP concerns, trust issues, and differing objectives	Africa Livestock Innovation Platform; AGRA	Co-design of technologies, benefit-sharing frameworks
Technology adoption readiness index	Composite measure assessing willingness and preparedness to adopt innovations.	Supports policy design by identifying gaps in adoption capacity.	Policymakers and development agencies	Lack of standardization, dynamic socio-cultural factors	Readiness Assessment Tools (e.g., IFAD, World Bank)	National readiness scorecards, targeted support programs

## 11 Research gaps and future perspectives

Beyond first-order technology deployment, complex, systemic challenges are becoming more and more prevalent in the evolution of global decarbonization strategies. The adoption of clean energy is progressing, but there are still significant gaps in the resilience of critical material supply chains and the construction of dependable negative emissions infrastructure, underscoring the urgent need for institutional innovation and research. Radical cross-sector interoperability will be necessary to close these gaps. For instance, the use of cutting-edge bioengineered materials, like insulation made of mycelium, necessitates extensive revisions to building codes, and next-generation grid controllers that use quantum optimization must work with changing hydrogen infrastructure in a variety of regulatory contexts (González & Zegarra, 2024).

The way forward necessitates changing institutional frameworks and market practices and goes far beyond technological advancements. AI-driven carbon accounting systems are quickly replacing conventional consultancy-based verification models, and innovative financial mechanisms, such as blockchain-enabled green bonds, are already upending traditional project finance timelines. These modifications show how quickly business and governance paradigms need to change to meet the increasing demands of the climate and resources (Eyo-Udo et al., 2024).

With markets and technologies coming together in unexpected ways, cross-sectoral integration is becoming more intense. While bioengineering approaches to ocean carbon sequestration face multi-layered regulatory complexities spanning both national food safety agencies and international maritime law, advancements like satellite-based detection of greenhouse gas emissions now directly interface with automated trading systems in carbon markets. These instances highlight how the research and regulatory environment is becoming more and more interdisciplinary (Adigun et al., 2024; Ling-Chin et al., 2024).

Preparing the workforce for a fair shift away from fossil fuel-based industries is another major challenge. Although there is still limited widespread adoption, emerging educational models, such as immersive and holographic training systems, show promise for quick reskilling. Tensions over patents for recycling rare earth elements and the competition for supremacy in solar waste processing technologies are two examples of how geopolitical dynamics continue to influence the availability of vital technologies and materials. These factors have the potential to reshape power dynamics in the developing circular economy (Vakulchuk & Overland, 2024).

Governance frameworks that are not only strong but also flexible enough to handle situations with a lot of uncertainty are desperately needed. Real-time treaty negotiation systems and dynamic contracts in energy markets that take climate-related changes in hydrological systems into account are early examples of this type of adaptive governance. These developments highlight the need to translate technological progress into cohesive sociotechnical systems that can adapt to escalating social, economic, and environmental stresses (Herizal et al., 2024).

Future studies must focus on converting laboratory-scale innovations, like highly efficient solar technologies, into practical and reasonably priced solutions that can be implemented in a variety of social settings, such as smallholder farming communities and informal urban settlements. The feasibility of achieving global mitigation and adaptation objectives will depend on the ability to align these innovations with land tenure systems, local governance frameworks, and socioeconomic realities (Makinde & Obikoya, 2024).

The ability of humanity to balance the pressing need to stabilize the climate with the exponential pace of technological advancement will ultimately be tested in the upcoming years. Technological advancements run the risk of falling behind accelerating planetary feedbacks without systemic integration and equitable deployment, endangering both global economic stability and climate goals. Research gaps, future perspectives, and technology horizons in GHG mitigation for livestock systems are presented in Table 10.

**Table 10:** Research gaps, future perspectives, and technology horizons in GHG mitigation for livestock systems.

Research/Technology area	Gap/Innovation focus	Emerging approaches/Technical thresholds	Potential/Projected impact	Feasibility/Scalability	Priority level/Partnerships
Enteric methane mitigation	Limited long-term efficacy data for natural feed additives	Precision microbiome engineering; CRISPR-based microbial editing	High potential for methane reduction at source	Medium to High	High
Manure management	Low adoption of advanced manure-to-energy technologies	Hydrothermal liquefaction; microbial electrolysis cells	Significant reduction in manure-related emissions	Medium	High
Precision livestock farming	Insufficient integration of multi-sensor platforms	IoT-integrated nanobiosensors; AI-driven decision tools	Improved GHG monitoring and targeted mitigation	High	High
Renewable energy integration	Economic and infrastructural barriers to hybrid systems	Modular hybrid microgrids; AI-optimized energy management	Transition to net-zero livestock farms	Medium	Medium
Climate modeling	Lack of downscaled, livestock-specific climate projections	Livestock-climate interaction simulators; multi-scenario modeling	Enhanced climate resilience in system design	Low to medium	High
Behavioral change and policy	Limited farmer adoption and policy enforcement	Behavioral nudges, gamified training platforms, and blockchain traceability	Improved compliance and voluntary emission reductions	High	Medium to high
CRISPR-engineered rumen microbiomes (2030)	Genetic manipulation of rumen microbes to reduce methane	Stable gene edits, animal health safety	Significant reduction in enteric methane	High in intensive systems	Geneticists, microbiologists, and regulatory agencies
Quantum-fed emission modeling	Use of quantum computing to simulate complex emission pathways	Quantum algorithm integration with livestock models	Improved accuracy in emission forecasting	Moderate due to computational demands	Tech companies and climate modelers
Self-powered barn sensors	Energy-harvesting sensors for real-time monitoring	Efficient energy conversion, network reliability	Enhanced monitoring and emission optimization	High with IoT frameworks	Engineers and agritech firms
Atmospheric methane oxidation	*In-situ* methane capture and oxidation systems	Catalytic efficiency and deployment infrastructure	Atmospheric methane reduction contribution	Low currently; potential for scale-up	Chemists and environmental tech developers
4D-printed feed additives	Smart feed delivery that adapts to the digestion process	Material innovation and bio-compatibility	Dynamic feed-based mitigation	Moderate to high	Material scientists and feed companies
Blockchain-enabled carbon markets	Decentralized, transparent carbon tracking	Data integration and system validation	Wider participation in carbon credits	High if standards align	Policy makers and blockchain developers

## 12 Conclusions

Global food security, climate change, and environmental stewardship are all intertwined with livestock systems. The increasing urgency of lowering greenhouse gas emissions from this industry necessitates not just small adjustments but revolutionary change propelled by innovation and science. Numerous next-generation strategies that focus on important emission sources throughout the livestock production cycle have been compiled in this review. The range and impact of technology are rapidly growing, ranging from genetic advancements and feed-based mitigation to digital precision tools and renewable energy solutions. The analysis emphasizes that no one strategy is adequate on its own. For significant and long-lasting GHG emission reductions, these technologies must be integrated at the systems level, adapted to particular production contexts, and backed by strong data infrastructures. Furthermore, it is still crucial to address obstacles pertaining to infrastructure, policy, cost, and adoption. This entails encouraging multidisciplinary cooperation, improving farmer involvement, and coordinating policy tools with carbon reduction incentives. Future developments in livestock GHG mitigation will depend on how well innovation, regulation, and behavior modification work together. It will be crucial to keep funding cutting-edge areas like smart sensors, AI-driven decision support, microbiome engineering, and hybrid energy platforms. In the end, developing climate-resilient livestock systems is globally necessary to achieve sustainable agriculture and planetary health, not just a scientific imperative.

## Funding information

This research received no funding.

## Data availability

Not applicable.

## Ethics statement

Not applicable: This manuscript does not include human or animal research.

## CRediT authorship contribution statement

**Navid Ghavi Hossein-Zadeh:** Writing – review & editing, Writing – original draft, Visualization, Validation, Supervision, Project administration, Investigation, Conceptualization.

## Declaration of competing interest

The authors declare that they have no known competing financial interests or personal relationships that could have appeared to influence the work reported in this paper.
